# The Effectiveness of Psychological Interventions for Families of Children With Type 1 Diabetes on Caregiver and Child Functioning: A Systematic Review and Meta‐Analysis

**DOI:** 10.1111/1753-0407.70112

**Published:** 2025-06-17

**Authors:** Katherine E. Wakelin, Rebecca K. Read, Ashely Y. Williams, Rachel R. Francois‐Walcott, Nicola O'Donnell, Rose‐Marie Satherley, Megan P. Harrington, Mary John, Christina J. Jones

**Affiliations:** ^1^ School of Psychology, Faculty of Health and Medical Science University of Surrey Guildford UK

**Keywords:** caregiver function, child function, family intervention, psychological intervention, Type 1 Diabetes

## Abstract

**Background:**

Research suggests that the wellbeing of caregivers of children with Type 1 Diabetes can influence child health outcomes. Therefore, the aim was to conduct a systematic review and meta‐analysis to estimate the effect of psychological interventions for families of children with Type 1 Diabetes on caregiver and child functioning.

**Methods:**

A systematic search of the literature identified 58 randomized controlled trials (RCTs) that met inclusion. Study quality was assessed using the Cochrane Risk‐of‐Bias Tool.

**Results:**

Fifty‐one trials had sufficient data to be included in the meta‐analysis, analyzing nine variables (caregiver and child psychological distress, diabetes distress, family conflict and child quality of life (QoL), diabetes QoL and blood glucose) over three timepoints (post‐intervention, short‐term and long‐term follow‐up). Results from 10 (*n* = 550), three (*n* = 347) and 16 RCTs (*n* = 1631) respectively indicated that psychological interventions significantly reduced caregiver psychological distress post‐intervention (SMD = −0.64, 95% CI = −1.15, −0.12), child psychological distress post‐intervention (SMD = −0.34, 95% CI = −0.55, −0.31) and child blood glucose at short‐term follow‐up (SMD = −0.11, 95% CI = −0.21, −0.01), relative to controls.

**Conclusions:**

Participants allocated to controls showed greater reductions in caregiver diabetes family conflict at short‐and long‐term follow−up than those assigned to psychological interventions. This was explained by significant baseline differences influencing a small number of studies. Studies were highly heterogenous regarding outcome measures, follow‐ups, and interventions, with high concerns of bias often observed, reflecting the complexity of real−world clinical practice. Findings are promising. Appropriately powered RCTs with robust randomization are recommended to investigate the significance of effects, whilst considering dose response.

## Graphical abstract

The systematic review and meta‐analysis of psychological interventions for families of children with Type 1 Diabetes on caregiver and child functioning found psychological interventions significantly reduced parent and child psychological distress post‐intervention and improved child blood glucose at short‐term follow‐up, relative to controls.

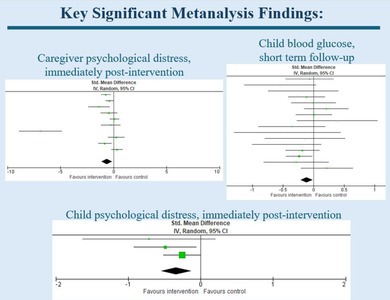

**Summary**

- A systematic review (*k* = 58) and meta‐analysis (*k* = 51) was conducted on randomized controlled trials (RCTs) to investigate the effectiveness of psychological interventions for families of children with Type 1 Diabetes on caregiver and child functioning.
- Psychological interventions showed a small–medium reduction in parent and child psychological distress post‐intervention and a small improvement in child blood glucose at short‐term follow‐up.
- Appropriately powered RCTs with robust randomization are recommended to investigate the magnitude of effects, whilst considering dose response.

## 1 Introduction

Caregivers of children with Type 1 Diabetes play a significant role in the management of their child's condition, typically overseeing responsibilities, such as blood glucose monitoring, insulin administration, meal planning, and appointment attendance [1]. Parents describe a balancing act of remaining vigilant in preventing hypoglycaemia and long‐term diabetes‐related complications, whilst ensuring their child's needs around independence and ownership of their diabetes care are met [2]. Parents can be vulnerable to exhaustion and feelings of powerlessness in dealing with their child's care, fuelling ‘diabetes burnout’ [3], and are at risk of experiencing clinically significant levels of anxiety and depression [4, 5]. The psychosocial adjustment process to their child's diagnosis can be marked by feelings of loss around their previous freedom, their child's health, and sense of safety in the world [6]. Research has suggested that around 15% of mothers of a child with Type 1 Diabetes will experience Post‐Traumatic Stress Disorder in relation to distressing events, such as severe hypoglycaemic attacks or hospital admissions [7].

Due to the important role that caregivers play in their child's Type 1 Diabetes management, it is not surprising that caregiver mental health difficulties can be associated with poorer health outcomes for children. Children whose mothers present with more severe symptoms of depression have higher utilization of diabetes‐related healthcare and are twice as likely to attend the emergency room [8, 9]. Depressive symptoms in caregivers have also been associated with less beneficial parental involvement in diabetes care, and such symptoms may undermine parents' ability to effectively help manage diabetes during their child's adolescence [10]. Similarly, higher levels of parental anxiety are associated with higher glycaemic levels in young adolescents, and lower autonomous motivations for diabetes care and positive affect in older adolescents [11]. Type 1 Diabetes tends to be associated with poorer family communication, affect management, and overall functioning [12]. Diabetes‐related conflict can arise due to disagreements between parent and child, and ineffective communication stemming from parental worries about the long‐term consequences of poor management [13, 14, 15]. This can lead to a vicious cycle, with family conflict being associated with poorer adherence and glycaemic control [16]. In contrast, cohesive family context can facilitate the achievement of Type 1 Diabetes individual's glycaemic goals [17]. Therefore, there is a clear rationale for understanding and supporting caregivers of children with Type 1 Diabetes, as this may contribute to improved outcomes for both caregivers and children.

The current literature on psychological interventions for caregivers and families of young people with Type 1 Diabetes is limited, with interventions drawing on a range of theories and models. Some are designed specifically for parents with a focus on their wellbeing, others target both children and parents, whilst others try to address family dynamics through family therapy approaches [18]. Previous systematic reviews of family‐cent red or parenting interventions for children with Type 1 Diabetes have neglected parent psychological outcomes [18, 19] and struggled with small samples [18, 20]. To the authors' knowledge, the only meta‐analysis to date investigating parenting interventions in this population did not consider child outcomes and completed searches in 2018 [21]. Therefore, the aim was to conduct an up‐to‐date systematic review and meta‐analysis to estimate the effect of psychological interventions for families of children with Type 1 Diabetes on both caregiver and child functioning. A meta‐analysis enables a systematic synthesis of the available evidence, even when heterogeneity exists, providing an overview to guide future research and clinical practice. High heterogeneity reflects the complexity of real‐world clinical practices. Therefore, a review that explores these variations can help inform the design of more standardized approaches.

## 2 Methods

A protocol for this systematic review was registered on the PROSPERO database (registration number: CRD42021247435). Since then, the scope of the review evolved to include child functioning, as well as parent functioning to assess the impact of psychological interventions more accurately on the whole family. To ensure the broadening of review scope remained manageable, the inclusion criteria were narrowed to only randomized control trials, as these are the gold standard design of research. As these revisions were made whilst the review was underway, they were unable to be prospectively logged in PROSPERO. The review is reported according to Preferred Reporting Items for Systematic Reviews and Meta‐Analyses (PRISMA) guidelines (Table S1).

### 2.1 Eligibility Criteria

Studies (*k*) were included if they (1) were designed as randomized controlled trials (RCTs) and published in English, (2) investigated the use of interventions based on psychological theory or evidence, (3) participants (*n*) were primary caregivers (not limited to biological parents) of children (below the age of 18) diagnosed with Type 1 Diabetes who were significantly involved in participating in the intervention, and (4) reported quantitative outcome measures of at least one of the following variables regarded as functioning: caregiver or child psychological distress, caregiver or child diabetes‐related psychological distress, caregiver or child family conflict, caregiver or child diabetes‐related family conflict, child quality of life, child diabetes quality of life, or child blood glucose levels. Like previous reviews [22], the broad definition of psychological intervention included a range of interventions, such as counseling therapy, family systems therapy, Cognitive Behavioural Therapy (CBT) and approaches which used techniques from CBT such as relaxation, goal‐setting, or problem‐solving. If the study involved caregivers or families of children with different physical health conditions, the study was only included if Type 1 Diabetes specific data could be extracted.

### 2.2 Search Strategy

Keyword, searches (Figure S1) were carried out on the November 22, 2022 and on January 1, 2025 to update the review. Searches were carried out on PsychINFO, PsychARTICLES, CINAHL, Medline, Child Development and Adolescent Studies, Web of Science and the Cochrane library. Studies published in English during any time period of any publication type were searched. Members of the research team RR NOD KW and SW screened the articles. To ensure consistency, 10% of studies were checked by two authors (RR and NOD) at the title and abstract screening stage and the full text screening stage, showing there was 83% and 84% agreement respectively, which increased to 100% after discussion.

### 2.3 Data Extraction

Data from each study was entered into a summary table to enable comparison of study characteristics. For the meta‐analyses, outcome data was extracted from each article for intervention and control groups (means, standard deviations, sample sizes). A non‐superiority approach was taken for three‐armed trials as no gold standard of psychological care for Type 1 Diabetes currently exists. Therefore, treatment as usual (TAU) controls were chosen instead of active controls when applicable. To be consistent, when a study had two active controls, the attention control was chosen over the competing intervention control, as this was the closest to a TAU condition [23]. Authors KW and AW extracted data. To ensure consistency, 10% of studies were checked by AW, showing 100% agreement after clarification. Author RF‐W also checked the accuracy of the summary table. When studies were eligible, but not all relevant data could be extracted from the publication, authors were contacted (*k* = 31). Studies were included in extraction even if they were unable to be included in the meta‐analysis. This was to give a comprehensive overview of study and sample characteristics and risk of bias in the field.

### 2.4 Quality Assessment of Studies

Each study included in the systematic review was assessed for bias using the Cochrane Risk‐of‐Bias Tool for Randomized Trials (RoB 2; 24). Ten percent of studies were checked for agreement on the RoB rating by author AW. The risk of bias in each subcategory was classified as high, low, or some concerns.

### 2.5 Data Analysis

All outcomes were measured as continuous data. Meta‐analyses were conducted using RevMan 5.4.1 software for each outcome immediately post‐intervention, at short‐term follow‐up (≤ 6 months) and long‐term follow‐up (> 6 months) when there were three or more RCTs for an outcome. As there were fewer than three RCTs measuring caregiver and child family conflict not specific to diabetes, these outcomes were unable to be included in the meta‐analysis. End point scores were expressed in standardized mean differences (SMDs) with 95% confidence intervals (CIs). Heterogeneity of studies was assessed by visually inspecting the forest plots and calculation of the *I* ^2^ statistic. However, in line with current guidance, a random‐effects model was used for all analyses regardless of *I* ^2^, as it was not possible to assume the different psychological interventions would produce identical effects [24]. Therefore, the model chosen was specifically designed to account for between‐study variability. When a study used a scale with a different scoring direction from the other scales used in an outcome category, the mean was multiplied by −1 to ensure that all the scales pointed in the same direction [24]. Funnel plots were visually inspected by KW to assess publishing bias.

## 3 Results

### 3.1 Study Selection

**FIGURE 1:**
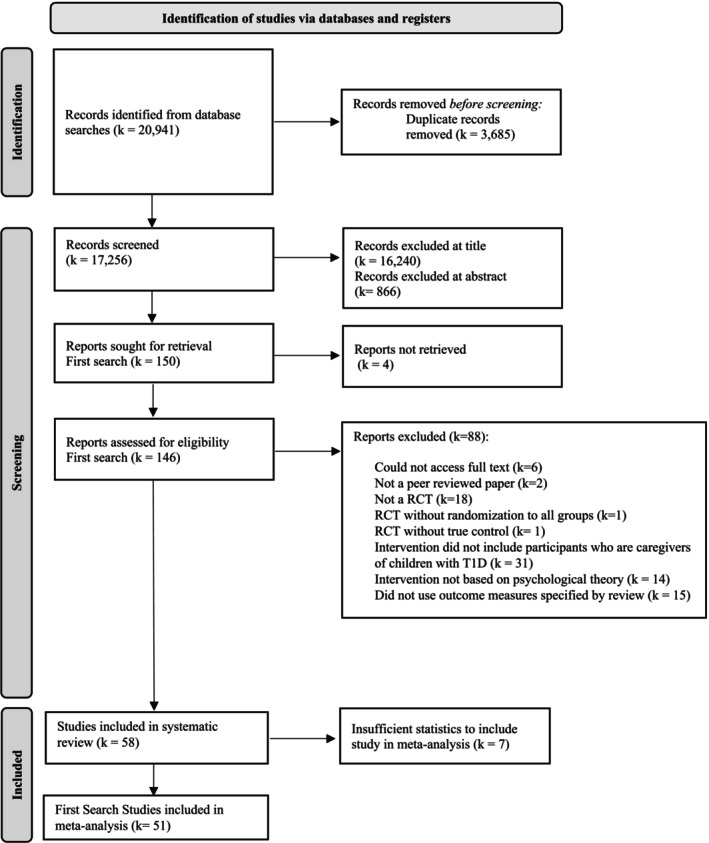
PRISMA flow diagram illustrating process of study selection.

The search strategy identified 20 941 studies. However, 3685 were excluded as duplicates and 17 106 excluded at title or abstract. The full texts of 146 were assessed for eligibility, and 58 met the criteria to include in the systematic review. However, only 51 studies were able to be included in the meta‐analyses. This was due to insufficient statistical reporting in seven studies that could not be resolved by contacting authors (Figure 1).

### 3.2 Sample and Study Characteristics

Table 1 highlights that the research was conducted in eight countries, the majority being the United States (76%). Only one study was conducted in a non‐Western country, Iran, meaning most samples included White Caucasian Americans from outpatient clinics. All studies were published between 1999 and 2024, and follow‐up periods varied from immediate post‐intervention to 24 months. Most studies were two‐arm trials with a TAU control (*k* = 36), rather than an active control (*k* = 9). However, 13 studies were three‐arm trials, with either an active and TAU control (*k* = 9), two active controls (*k* = 1) or an active control and combined condition (*k* = 3). Table S2 illustrates the range of measures used for the difference outcomes, highlighting caregiver psychological distress to have the greatest measure variety (*k* = 10) and child quality of life to have the least (*k* = 1). Table S3 provides the references for the review articles.

**TABLE 1:** Sample and study characteristics.

Study; country; setting; *N* post int and FU (Int/TAU/Active Con)	Intervention content	Intervention duration	Control/s	Sample mean age and T1D duration (SD)	Gender (% female) and ethnicity	Caregiver outcome measures	Child outcome measures
Ambrosino et al., 2008; USA; Outpatients; 1 month FU: 81 (51/30)^a^; 3 month FU: 79 (49/30)	Description: Coping skills training; Theory: Forman (1993); Facilitator: Mental health professional; Goal: To increase sense of competence by teaching more positive styles and patterns of behavior; Topics: Communication, social problem solving, associations between thoughts, feelings, and actions, stress management, conflict resolution.	Length of time: Not stated; Amount: Six 1.5 h sessions; Child and Parent: Separately; Delivery: F2f groups.	Control: Active Description: Group education. Facilitator: Nurse practitioner. Goal: Provide specific information on what to do in multiple diabetes‐related situations. Topics: Intensive insulin regimens, carbohydrate counting, sports and sick days.	Age: Caregiver Int: 39.3 (4.9); Caregiver Con: 41.2 (6.0); Child Int: 9.9 (1.5); Child Con: 9.9 (1.4) T1D duration: Int: 3.7 (2.8) Con:3.8 (3.2)	Female: Caregiver Int: 93%; Caregiver Con: 97%; Child Int: 56%; Child Con: 70% Ethnicity: Int White: 82%; Active Con White: 91% (Child and Caregiver not stated)	Psych Distress: CESD; Diabetes Psych Distress: Issues Coping IDDM‐Upset^b^; Diabetes Family Conflict: DFCS‐Conflict^b^	Diabetes Distress: IDDM‐Upset; Diabetes QoL: DQOL‐Y‐Satisfaction; Blood Glucose: HbA1c (%; Clinic reported)^b^
Anderson et al., 1999; USA; Outpatients; Post‐int: 85 (28/27/30); 12 month FU HbA1c only: Assumed 85 (28/57)	Description: Parent‐teen teamwork training; Theory: Not stated; Facilitator: Research assistant trained in Diabetes care using scripted protocol; Goal: Increase parent‐teen responsibility sharing for diabetes tasks to reduce conflict; Topics: Causes of high and low blood glucose, realistic expectations for blood glucose and behaviors, parental involvement without shaming the child.	Length of time: 12 months; Amount: Four 20–30 min sessions every 3–4 months; Child and Parent: Together; Delivery: F2f with individual family.	Control 1: TAU Control 2^c^: Active; Description: Diabetes education, with no focus on parental involvement; Facilitator: Research assistant trained in Diabetes care. Goal: Improve understanding of T1D. Topics: Telling others about T1D, effects of stress and exercise and healthy meal choices.	Age: Child Int: 12.7 (1.4); Child Active Con: 12.7 (1.4); Child TAU: 12.5 (1.4); Caregiver: Unknown. T1D duration: Int: 5.3 (2.6); Active Con: 6.1 (2.8); TAU: 5.2 (2.2)	Gender: Child Int: 50%; Child Active Con: 50%; Child TAU: 48%; Caregiver: Unknown. Ethnicity: Not stated.	Diabetes Family Conflict: DFCS	Blood Glucose: HbA1c (%; Clinic reported)
Christie et al., 2014; UK; Outpatients; 8 month FU^a^: 298 (143/155); 20 month FU: 284 (135/149)	Description: Structured educational group programme incorporating psychological approaches; Theory: Motivational interviewing and Solution Focused Brief Therapy. Facilitator: Pediatric diabetes nurse with another team member. Goal: Improve long‐term glycaemic control, quality of life and psychosocial functioning in young people; Topics: Relationship between food, insulin and blood glucose, communication skills, identifying strengths, focusing on the future, scaling and pros and cons techniques.	Length of time: 4 months approx; Amount: Four 2‐h sessions; Child and Parent: Together; Delivery: F2f groups.	Control: TAU	Age: Child Int: 13.1 (2.1); Child Con: 13.2 (2.1); Caregiver: Not stated. T1D duration: Int: 5.7 (3.2); Con: 6.1 (3.3).	Gender: Child Int: 57%; Child Con: 54%; Caregiver Int: 87%; Caregiver Con: 91%; Ethnicity: Child Int White British: 84%; Child Con White British: 77%; Caregiver Int White British: 87%; Caregiver Con White British: 78%	N/A	Diabetes Distress: PedsQL‐Diabetes‐Worry; Quality of Life: PedsQL‐Generic; Blood Glucose: HbA1c (%).
Commissariat et al., 2023 USA; Outpatient; Post‐int: 137 (48/46/43) 6 month FU: (45/44/42)	Description: Continuous glucose monitoring (CGM) with a family behavioral intervention (FBI); Theory: Not stated; Facilitator: Trained research assistant; Goal: To improve glycaemic outcomes and parent quality of life; Topics: Family feelings, attitudes, and behaviors that can be barriers to CGM; skills to manage behavioral barriers	Length of time: 6 months; Amount: nine sessions, four were 30 min of CGM training and five were 30 min of FBI training; Child and Parent: Together; Delivery: Individual family, F2F or telephone depending on participant preference.	Control 1: TAU for 6 months, then Active Control of Standard care alongside CGM for 6 months; Control 2^d^: Active Con; Description: Standard care alongside CGM.	Age: Child Int: 5.7 (1.6); Child TAU then Active Con: 6.1 (1.7); Child Active Con: 5.1 (1.8); Caregiver: Not stated. T1D duration: Child Int: 2.4 (1.9); Child TAU then Active Con: 2.7 (2.0); Child Active Con: 1.8 (1.7)	Gender: Child Int: 58%; Child TAU then Active Con: 50%; Child Active Con: 40%; Caregiver: Not stated. Ethnicity: Child Int Non‐Hispanic White: 68%; Child Int Non‐Hispanic Black: 16%; Child Int Hispanic or Latino: 11%; Child Int other: 5%; Child TAU then Active Con Non‐Hispanic White: 63%; Child TAU Then Active Con Non‐Hispanic Black: 14%; Child TAU then Active Con Hispanic or Latino: 12%; Child TAU then Active Con Asian: 2%; Child TAU then Active Con other: 9% Child Active Con Non‐Hispanic White: 76%; Child Active Con Non‐Hispanic Black: 10%; Child Active Con Hispanic or Latino: 12%; Child Active Con other: 1% Caregiver: Not Stated.	Diabetes Distress: PAID‐PR^b^ (Post‐int excluded as reported in Laffel et al., 2021) Psych distress: WHO‐5	N/A
Coyne et al., 2024; Ireland; Community; 6–8 month FU: 94 (46/48)	Description: Video and questions to promote adolescents' communication in clinic; Theory: Intervention informed by Bandura's social cognitive theory and self‐efficacy; Facilitator: Self‐directed; Goal: To improve adolescents' question asking and provider education during pediatric clinic visits. Topics: Empowerment, managing your diabetes so you can get on with the fun stuff in life, independence, becoming comfortable to speak and ask questions at clinic visits, advice on how to ask questions	Length of time: One clinic visit; Amount: 9 min video and reading through question prompt list within 1.5 h; Child and Parent: Together; Delivery: Remote, self‐directed.	Control: TAU	Age: Child Int: 13.00 (1.5); Child Con: 13.08 (1.8); Caregiver: Not stated. T1D duration: Int: 5.97 (3.3); Con: 5.40 (3.0).	Gender: Child Int: 57%; Child Con: 50%; Caregiver Int: 80%; Caregiver Con: 67% Ethnicity: Child Int Caucasian: 96%; Child Con Caucasian: 96%; Child Int Non‐White: 4%; Child Con Non‐White: 4%	N/A	Blood Glucose: HbA1c (%; Clinic reported)
Doherty et al., 2014; UK; Community; Post‐int: 54 (22/32)	Description: Self‐directed behavioral social learning; Theory: Behavioral family intervention, based on social learning principles; Facilitator: Self‐directed; Goal: To help parents build on their existing skills and information to practice positive parenting; Topics: Positive parent–teenager relations, increasing desirable behavior, new behaviors and skills and management of problem behavior.	Length of time: 10 weeks approx; Amount: 1 h a week; Child and Parent: Parent only; Delivery: Remote, self‐directed worksheets, individual.	Control: W/L with TAU; Description: After 10‐week study period received the self‐directed intervention resources.	Age: Child Int: 13; Child Con: 13; Caregiver Int: 43; Caregiver Con: 44 (Only Md available, No SDs stated). T1D duration: Int: 4.3 (3.8); Con: 6.0 (2.8).	Gender: Child Int: 36%; Child Con: 51%; Caregiver Int: 98%; Caregiver Con: 100% Ethnicity: Not stated.	Diabetes Distress: PIP; Diabetes Family Conflict: DFCS‐R	N/A
Ellis et al., 2005; USA; Outpatient; 1 month FU: 110 (57/53)	Description: Multisystemic therapy; Theory: CBT, parent training, and behavioral family systems therapy; Facilitator: Therapists and Supervisors; Goal: To decrease diabetes‐related stress by targeting adherence‐related problems; Topics: Multisystemic assessment of the family's strengths and weaknesses to develop tailored treatment goals and interventions	Length of time: 6 months approx; Amount: 2–3 sessions per week; Child and Parent: Together; Delivery: F2F, with individual family.	Control: TAU	Age: Child Int: 13.4 (1.9); Child Con: 13.1 (2.0); Caregiver Int: 39.7 (7.7); Caregiver Con: 37.9 (5.9) T1D duration: Int: 5.3 (3.9); 5.2 (4.8)	Gender: Child Int: 41%; Child Con: 62%; Caregiver: Not stated. Ethnicity: Child Int African American: 69%; Child Con African American: 57%; Child Int White: 20%; Child Con White: 30%; Caregiver: not stated	N/A	Diabetes Distress: DSQ
Ellis et al., 2007 USA; Outpatient; 1 month FU: 118 (59/58) 6 month FU: 101 (49/52)	Description: Multisystemic therapy; Theory: CBT, parent training, and behavioral family systems therapy; Facilitator: Therapists and Supervisors; Goal: To decrease diabetes‐related stress by targeting adherence‐related problems; Topics: Multisystemic assessment of the family's strengths and weaknesses to develop tailored treatment goals and interventions	Length of time: 6 months approx; Amount: 2–3 sessions per week; Child and Parent: Together; Delivery: F2F, with individual family.	Control: TAU	Age: Child Int: 13.4 (1.9); Child Con: 13.1 (2.0); Caregiver Int: 39.7 (7.7); Caregiver Con: 37.9 (5.9) T1D duration: Not stated.	Gender: Child Int: 41%; Child Con: 62%; Caregiver: Not stated. Ethnicity: Child Int African American: 69%; Child Con African American: 57%; Child Int White: 20%; Child Con White: 30%; Caregiver: not stated	N/A	Blood Glucose: HbA1c (%; Clinic reported)
Ellis et al., 2017; USA; Outpatient; 1 month FU: 64 (24/23/17)	Description: Computer‐delivered motivational intervention; Theory: Information‐motivation‐behavioral skills model of health behavior change; Facilitator: Self‐directed with animated narrator; Goal: Motivate family to complete diabetes management; Topics: Psychoeducation, parenting skills, parental monitoring, motivational elements, pros and cons of behavior change, and goal setting.	Length of time: 6 months; Amount: 3 sessions, each delivered at 2‐month intervals; Child and Parent: Separately; Delivery: Individual, remote.	Control 1: Active; Description: Attention control for parent and child; Facilitator: Animated narrator on online software; Goal: Increase understanding of T1D; Topics: risks associated with smoking, traveling, and emergency preparedness for persons with T1D. Control 2^c^: Active; Description: Computer‐delivered motivational intervention for parent and attention control for child; Facilitator: Animated narrator on online software; Goal: Increase understanding of T1D; Topics: risks associated with smoking, traveling, and emergency preparedness for persons with T1D.	Age: Child: 12.1 (1.3); Caregiver: 38.3 (6.6). T1D duration: 4.6 (3.1). (Condition breakdown not stated).	Gender: Child: 56%.; Caregiver: 88%. Ethnicity: Caregiver African American: 83% (Condition breakdown not stated).	N/A	Family Conflict: PARQ‐Global Distress^e^; Blood Glucose: HbA1c (%; Clinic reported)
Ellis et al., 2019; USA; Outpatient; 1 month FU: 47 (23/24)	Description: Multisystemic therapy; Theory: Multisystemic family therapy, using the family‐focused treatment approach; Facilitator: Two trained community health workers; Goal: Improve health status and quality of life in high‐risk, low‐income families; Topics: Relationship building, relapse prevention, and cognitive behavioral skill training involving problem solving, behavioral contracting, and improving family communication.	Length of time: 6 months; Amount: 1 or 2 sessions a week, for 30–90 min; Child and Parent: Together; Delivery: F2F with individual family.	Control: TAU	Age: Child Int: 13.5 (2.2); Child Con: 15.0 (2.3); Caregiver: Not stated T1D duration: Int: 5.9 (3.3); Con: 7.4 (3.9)	Gender: Child Int: 74%; Child Con: 50%; Caregiver: Not stated. Ethnicity: Child Int African American: 83%; Child Con African American: 75%; Caregiver: Not stated	N/A	Diabetes QoL: DQOL‐Y; Blood Glucose: HbA1c (%; Clinic reported)
Fiallo‐Scharer et al., 2019; USA; Outpatient; 3 Month FU^a^: PedsQL only: 187 (93/94); 3 Month FU HbA1c only: 174 (82/92); 6 Month FU PedsQL only: 158 (80/78); 9 Month FU PedsQL only: 191 (93/98); 12 Month FU HbA1c only: 187 (91/96)	Description: Family tailored self‐management resources; Theory: Behavioral Family Systems Therapy and motivational interviewing; Facilitator: Social workers, nurses, psychologists, and diabetes educators; Goal: To reduce T1D self‐management barriers in families; Topics: Group materials on barriers including motivation, understanding and organizing care, and family interactions.	Length of time: 12 months; Amount: Four 75‐min sessions; Child and Parent: Together; Delivery: F2F groups of families.	Control: TAU	Age: Child Int: 13.2 (2.6); Child Con: 13.2 (2.2); Caregiver: Not stated T1D duration: Site 1 Youth Int: 4.1 (2.4); Site 1 Youth Con: 3.9 (2.3); Site 1 Teen Int: 6.6 (3.7); Site 1 Teen Con: 7.0 (3.9); Site 2 Youth Int: 4.6 (2.7); Site 2 Youth Con: 5.3 (3.1); Site 2 Teen Int: 5.7 (3.1); Site 2 Teen Con: 5.7 (3.9) (Data collapsed across sites and ages not stated).	Gender: Child Int: 43%; Child Con: 55%; Caregiver Int: 79%; Caregiver Con: 90%. Ethnicity: Child Int White: 82%; Child Con White: 85%; Caregiver Int White: 85%; Caregiver Con White: 91%.	N/A	Diabetes Distress: PedsQL‐Diabetes‐R‐Worry; Diabetes QoL: PedsQL‐Diabetes‐R^f^; Blood Glucose: HbA1c (% and mmol/mol; Clinic reported)
Forsander et al., 2000; Sweden; Outpatient; 2 Year FU^a^: 38 (19/19); 5 Year FU: 38 (19/19)	Description: Individualized, family‐therapeutic care system; Theory: Intervention based on a system theory; Facilitator: Members of the diabetes team including a family therapist; Goal: To support positive coping strategies and decrease family stress by offering active crisis support, buffering social weakness, and stimulating the learning procedure; Topics: Problem‐based education and promoting social support by inviting extended family and friends to information and informal meetings with the diabetes team.	Length of time: 3 weeks; Amount: Not stated; Child and Parent: Together; Delivery: F2F, with individual family.	Control: TAU	Age: Child Int: 9.8; Child Con: 9.6; Caregiver: Not stated (Only Md available, No SDs stated). T1D duration: Not stated.	Gender: Child Int: 63%; Child Con: 74%; Caregiver: Not stated. Ethnicity: Not stated	N/A	Blood Glucose: HbA1c (%)
Gregory et al., 2011; UK; Outpatient; Post‐int: 677 (354/323)	Description: Healthcare appointments conducted by a healthcare professional who completed a healthcare communication training programme; Theory: Constructive behavior change conversations; Facilitator: Healthcare professional; Goal: To support children and their families to cope better with restrictions associated with diabetes management; Topics: Agenda setting, flexible styles of communication and a menu of strategies to be used in consultations with families.	Length of time: 12 months; Amount: An average of 4 appointments a year; Child and Parent: Together; Delivery: F2F with individual family.	Control: TAU.	Age: Child Int: 10.4 (2.8); Child Con: 10.7 (2.8); Caregiver: Not stated. T1D duration: Child Int: 5.2 (2.8); Child Con: 5.0 (2.7)	Gender: Child Int: 48%; Child Con: 54%; Caregiver Int: 93%; Caregiver Con: 93%. Ethnicity: Child Int White: 91%; Child Con White: 91%; Caregiver: Not stated.	Diabetes Distress: PAID‐Modified	Diabetes Distress: PedsQL‐Diabetes‐Worry; Blood Glucose: HbA1c (%; Clinic reported)
Gray et al., 2011; USA; Outpatient; Post‐int^g^: 129 (79/50); 3‐month FU^a^: 121 (75/46); 6‐month FU: 120 (72/48); 12‐month FU: 112 (69/43)	Description: Coping skills parent training groups; Theory: Social Cognitive Theory (Bandura, 1986); Facilitator: Healthcare professionals; Goal: To create positive and adaptive coping behaviors which increase children's and parents' sense of competence and mastery; Topics: Communication, social problem‐solving, cognitive restructuring, stress management, and conflict resolution.	Length of time: Unknown; Amount: Six 1.5 h sessions; Child and Parent: Parent only; Delivery: F2F with groups of parents.	Control: Active; Description: Group education; Facilitator: Nurse practitioner; Goal: Provide educational understanding of T1D; Topics: Intensive insulin regimens, carbohydrate counting, sports and sick days.	Age: Child Int: 8.1 (2.9); Child Con: 7.9 (2.8); Caregiver: Not stated. T1D duration: Not stated.	Gender: Child Int: 54%; Child Con: 35%; Caregiver: Not stated. Ethnicity: Not stated.	Psych Distress: CESD^f^; Diabetes Family Conflict: DFCS‐conflict; Diabetes Distress: IDDM	Blood Glucose: HbA1c (%; Clinic reported)
Hannon et al., 2019; USA; Outpatient; Post‐int: 90 (26/33/31)	Description: Family‐centred goal setting; Theory: Motivational interviewing and a goal setting tool; Facilitator: Health educator, diabetes educator, nurse practitioner and pediatric endocrinologist; Goal: To improve self‐monitoring of blood glucose and diabetes self‐care; Topics: Frequency of self‐monitoring of blood glucose, giving bolus insulin according to recommendations, self‐adjustment of insulin, parental nagging and oversight, frequency of contact with diabetes care team.	Length of time: 6 months; Amount: Two 30‐min sessions; Child and Parent: Together; Delivery: F2F with individual family.	Control 1: Active; Description: Technology‐enhanced blood glucose meter to share data with patients, parent, and care providers; Facilitator: Health technology; Goal: Improve self‐monitoring of blood glucose and diabetes self‐care; Topics: n/a. Control 2^c^: Combined; Description: Both family‐centred goal setting intervention and enhanced blood glucose meter control.	Age: Child Int: 14.7 (1.5); Child Active Con: 14.5 (1.7); Child Combined Con: 14.7 (2.0); Caregiver: Not stated T1D duration: Child Int: 5.0 (3.5); Child Active Con: 5.8 (3.8); Child Combined Con: 6.1 (4.2)	Gender: Child Int: 49%; Child Active Con: 52%; Child Combined Con: 48%; Caregiver: Not stated. Ethnicity: Child Int White: 85%; Child Active Con White: 88%; Child Combined Con White: 74%; Caregiver: Not stated	N/A	Blood Glucose: HbA1c (%; Clinic reported)
Harris et al., 2001; USA; Outpatient; Post‐int:119 (39/40/40)	Description: Address parent‐adolescent conflict from both behavioral and family system perspectives; Theory: Behavioral Family Systems Therapy (Robin and Foster, 1989); Facilitator: Doctoral level psychologists; Goal: To reduce diabetes‐related conflict for families; Topics: Targeted problem solving, communication skills, irrational beliefs, and structural family problems.	Length of time: 3 months; Amount: 10 sessions; Child and Parent: Together; Delivery: F2F with individual family.	Control 1: TAU. Control 2^c^: Active; Description: 10 sessions of a multi‐family educational support group.	Age: Child Int: 14.5; (1.2) Child Active Con: 14.1 (1.4); Child TAU: 14.3 (1.3); Caregiver: Not stated. T1D duration: Child Int: 5.4 (3.8); Child Active Con: 4.5 (3.7); Child TAU: 5.5 (3.3)	Gender: Child Int: 56%; Child Active Con: 50%; Child TAU: 63%; Caregiver: Not stated. Ethnicity: Child Int White: 79%; Child Active Con White: 80%; Child Con TAU White: 80%; Caregiver: Not stated.	Diabetes Family Conflict: DFCS	Diabetes Family Conflict: DFCS
Hillard et al., 2020; USA; Outpatient; Post‐int: 78 (54/24)	Description: Strengths‐based web app intervention installed on parents' phones; Theory: Diabetes resilience Model; Facilitator: Online app; Goal: Promote supportive family diabetes management by helping parents to recognize and reinforce teen's positive diabetes‐related behaviors; Topics: Psychoeducation, rating and synthesis of child's strengths, diabetes strengths, and delivering praise.	Length of time: 3–4 months; Amount: App could be used daily between two diabetes appointments spaced 3 months apart; Child and Parent: Parent only; Delivery: Remote, Individual.	Control: TAU	Age: Child: 15.3 (1.5); Caregiver: Not stated. T1D duration: 5.7 (3.4) (Condition breakdown not stated).	Gender: Child: 59%; Caregiver: 80% (Condition breakdown not stated). Ethnicity: Child Non‐Hispanic White: 61%; Child Hispanic: 19%; Child Non‐Hispanic Black: 13%; Child other: 7%; Caregiver: Not stated (Condition breakdown not stated).	Diabetes Psych Distress: PAID‐PR; Diabetes Family Conflict: DFCS‐R	Diabetes Distress: PAID‐T; Diabetes Family Conflict: DFCS‐R; Diabetes QOL: MY‐Q; Blood Glucose: HbA1c (%)
Hillard et al., 2022; USA; Outpatient; Post‐int: 156 (116/41); 6 month FU: (115/41)	Description: Step 1—Parent peer support coaching. Step 2—Individual and group psychoeducation. Step 3—Professional consultation; Theory: Social Cognitive Theory; Facilitator: Step 1 was delivered by a parent coach who was a parent of a child with T1D. Step 2 was delivered by a master's level study interventionist. Step 3 was delivered by a diabetes educator and a licensed psychologist; Goal: To support parents' psychosocial functioning and promote children's glycaemic outcomes; Topics: Step 1—Parent's shared expertise and emotional support. Step 2—Psychoeducation for managing depressive symptoms, parenting young children, problem‐solving, self‐care, and gratitude. Step 3—Support and strategies for parents for their child's psychosocial functioning and diabetes and behavior management.	Length of time: 9 months; Amount: Step 1—Weekly; Step 2—Five sessions within 3 months; Step 3—Two sessions; Child and Parent: Parent only; Delivery: F2F, Individual with 1 group session.	Control: TAU	Age: Child: 4.5 (1.6); Caregiver: 34.9 (7.0). (Condition breakdown not stated). T1D duration: Not stated.	Gender: Child: 55%; Caregiver: 92% (Condition breakdown not stated). Ethnicity: Child non‐Hispanic White: 60%; Child Hispanic: 15%; Child non‐Hispanic Black: 15%; Child other: 7%; Caregiver Non‐Hispanic White: 62%; Caregiver Hispanic: 12%; Caregiver Non‐Hispanic Black: 14%; (Condition breakdown not stated).	Psych Distress: CESD	Blood Glucose: HbA1c (%; Clinic reported)
Hoff et al., 2005; USA; Outpatient; 1 Month FU: 29 (15/14); 6 Month FU: 24 (11/13)	Description: Manualised Parent group to teach skills to manage uncertainties; Theory: Socioecological model and cognitive theory of uncertainty; Facilitator: Advanced Clinical psychology graduate students; Goal: To decrease parental uncertainty and distress; Topics: Defining and exploring illness uncertainty. Management techniques of illness uncertainty including information resources, problem‐solving and communication skills, and role clarification.	Length of time: 1 week; Amount: Two 2.5‐h sessions delivered over two consecutive weekends; Child and Parent: Parent only; Delivery: F2F parent groups.	Control: WL with TAU; Description: After study period invited to participate in the intervention.	Age: Child Int: 9.3 (4.7); Child Con: 9.4 (3.4); Mother Int: 39.3 (7.6); Mother Con: 37.5 (5.0). T1D duration: Not stated.	Gender: Child Int: 53%; Child TAU: 41%; Caregiver Int: 55%; Caregiver TAU: 60% Ethnicity: Int Caucasian: 82%; Con Caucasian: 100% (Caregiver and child breakdown not stated).	Psych Distress: SCL‐90‐R‐GSI	N/A
Holmes et al., 2014; USA; Outpatient; Post‐int: 214 (125/89); 6 Month FU: 182 (111/71); 12 Month FU^a^: 149 (91/58); 18 Month FU^a^: 130 (81/49); 24 Month FU: 136 (80/56)	Description: Coping skills education; Theory: Family teamwork model drawing on behavior change and cognitive behavior therapy; Facilitator: Graduate level interventionists; Goal: To facilitate diabetes management and promote effective family interventions; Topics: Discussion of developmental challenges of diabetes regimen. Coping skills such as attitude and behavior change. Problem solving of blood glucose monitoring, conflict resolution surrounding dietary issues, parental support and cognitive reframing to promote exercise.	Length of time: 1 week; Amount: Four 30–45‐min sessions, each delivered at 3‐month intervals; Child and Parent: Together; Delivery: F2F, with individual family.	Control 1: Active; Description: Non‐individualized diabetes education in four 15–20‐min sessions; Facilitator: Graduate level facilitators; Goal: Improve understanding of T1D; Topics: Communication about diabetes, extracurricular activities, travel, school issues, 504 education plans, and diabetes rights. Control 2^d^: TAU	Age: Child Int: 13.0 (1.2); Child Active Con: 12.7 (1.2); Child TAU: Not stated; Caregiver: Not stated. T1D duration: Not stated.	Gender: Child Int: 56%; Child Active Con: 46%; Child TAU: Not stated; Caregiver: 92% (Caregiver condition breakdown not stated) Ethnicity: Child Int Non‐White: 32%; Child Active Con Non‐White: 25%; Child TAU: Not stated; Caregiver: Not stated.	Diabetes Family Conflict: DFCS‐R; Family Conflict: FES‐Conflict^e^	Diabetes Distress: PedQL‐Diabetes‐Worry (but not collected at 6 and 18 month FUs); Diabetes Family Conflict: DFCS‐R; Family Conflict: FES‐Conflict^e^; Blood Glucose: HbA1c (% and mmol/mol; Clinic reported)
Holtz et al., 2024; USA; Outpatient; Post‐int: 33 (23/10)	Description: App based family communication; Theory: Social cognitive theory and self‐determination theory; Facilitator: Self‐facilitated via app; Goal: To promote effective diabetes communication between adolescents and parents; Topics: Blood glucose schedule set via app, reminder prompts, links to videos of other adolescents with T1D, and affirming messages.	Length of time: 12 Weeks; Amount: At least four times a day; Child and Parent: Together; Delivery: Remote, with individual family.	Control: TAU	Age: Child Int: 12.5 (1.5); Child Con: 12 (1.9); Caregiver: Not stated. T1D duration: Child Int T1D 1–5 years: 52%; Child Con T1D 1–5 years: 60% (Mean and SD not stated).	Gender: Child Int: 65%; Child Con: 50%; Caregiver Int: 86%; Caregiver Con: 80% Ethnicity: Child Int White: 96%; Child Con White: 80%; Caregiver: Not stated.	N/A	Quality of Life: PedsQL‐Generic; Blood Glucose: HbA1c (%; Clinic reported)
Husted et al., 2014^h^; Denmark; Outpatient; Post‐int: 57 (26/31); 6 Month: 53 (23/30)	Description: Guided self‐determination youth intervention; Theory: Life skills approach; Facilitator: Pediatric physicians, pediatric diabetes nurses and dieticians; Goal: To aid families to systematically explore and express their individual and shared difficulties; Topics: Semi‐structured reflection sheets for adolescents and parents. Health care professionals reflected on the sheets using mirroring, active listening, and values‐clarifying responses	Length of time: 8–12 months; Amount: Eight 1‐h sessions; Child and Parent: Together; Delivery: F2F, with individual family.	Control: TAU	Age: Child Int: 14.9 (1.5); Child Con: 14.6 (1.3); Caregiver: Not stated. T1D duration: Child Int: 6.1 (3.0); Child Con: 9.2 (3.7)	Gender: Child Int: 62%; Child Con: 60%; Caregiver: Not stated. Ethnicity: Child Int Danish: 84%; Child Con Danish: 74%; Caregiver: Not stated.	N/A	Psych Distress: WHO‐5^f^; Diabetes Distress: PAID^f^; Blood Glucose: HbA1c (% and mmol/mol; Clinic reported)^f^
Jaser et al., 2014; USA; Outpatient; 1 Month FU^a^: 37 (19/18); 4 Month FU: 36 (18/18)	Description: A gift was mailed to the child every 2 weeks. They were instructed to notice and remember things that made them happy and proud when it was time to check their blood sugar; Theory: Positive psychology exercises; Facilitator: Not stated; Goal: Increase positive affect through gratitude, self‐affirmation, small gifts, and parental affirmations; Topics: Positive psychology exercises including gratitude and self‐affirmation; small gifts; and parent affirmations.	Length of time: 8 weeks; Amount: One phone call, every 2 weeks; Child and Parent: Together; Delivery: Remote telephone call, with individual families.	Control: Active Control; Description: Mailed diabetes educational materials every 2 weeks for 8 weeks; Facilitator: Self‐directed; Goal: Increase understanding of T1D; Topics: Hypoglycaemia; hyperglycaemia; driving and diabetes; and exercise.	Age: Child Int: 15.3 (1.4); Child Con: 15.0 (1.6); Caregiver: Not stated. T1D duration: Child Int: 7.3 (4.3); Child Con: 6.5 (3.5)	Gender: Child Int: 65%; Child Con: 50%; Caregiver Int: 86%; Caregiver Con: 80% Ethnicity: Child Int White: 96%; Child Con White: 80%; Caregiver: Not stated.	Diabetes Family Conflict: DFCS	Psych Distress: CDI; Diabetes Family Conflict: DFCS; Diabetes QoL: PedsQL‐Diabetes; Blood Glucose: HbA1c (%)
Jaser et al., 2018; USA; Outpatient; Post‐int psychometrics only: 20 (9/11); Post‐int HbA1c only: 30 (15/15)	Description: Telephone CBT intervention for mothers; Theory: CBT and Social learning principles using in the Positive Parenting Program; Facilitator: A master's level interventionist; Goal: To reduce maternal distress, improve parenting practices, and reduce family conflict; Topics: Coping strategies such as cognitive reappraisal positive thinking, acceptance and distraction to reduce maternal depressive symptoms. Principles of positive parenting such as positive parent–child relationships and reinforcing and managing behaviors.	Length of time: 13 weeks; Amount: Five weekly sessions followed by two monthly booster sessions; Child and Parent: Parent only; Delivery: One F2F session, followed by remote sessions, Individual.	Control: TAU	Age: Child: 13.4 (1.8); Caregiver: 42.5 (5.1). (Condition breakdown not stated). T1D duration: Not stated.	Gender: Child: 38%; Caregiver: 100% (Condition breakdown not stated) Ethnicity: Child White/non‐Hispanic: 93%; Caregiver White/non‐Hispanic: 100%. (Condition breakdown not stated).	Psych Distress: PHQ‐9; Diabetes Distress: DDS‐P; Diabetes Family Conflict: DFCS‐R	Psych Distress: CDI Diabetes Family Conflict: DFCS‐R; Diabetes QoL: PedsQL‐Diabetes; Blood Glucose: HbA1c (%; Clinic reported)
Jones et al., 2024; UK; Outpatient; 1 Month FU^a^: 55 (27/28); 3 Month FU: 55 (29/26)	Description: Parent Psychoeducation group; Theory: Information motivation behavioral skills model alongside cognitive and emotional aspects; Facilitator: Psychology researchers and a website with downloadable resources; Goal: To prevent the development of disorder eating in children and young people and improve parental wellbeing; Topics: Appropriate communication around food and weight for early recognition of disordered eating; encouraging identity formation and independence of T1D manage in children and young people; and integrating strategies into daily life.	Length of time: 2 weeks; Amount: Two‐hour sessions delivered twice weekly; Child and Parent: Parent only; Delivery: Remote, group.	Control: W/L with TAU; Description: After 3‐months received the intervention materials.	Age: Child: 12.3 (1.1); Caregiver: 47.8 (5.2) T1D duration: Child mean age of T1D onset: 8.20 (3.26) (mean duration of T1D not stated) (Condition breakdown not stated).	Gender: Child: 47%; Caregiver: 89% (Condition breakdown not stated). Ethnicity: Child White: 78%; Child Other Ethnic Background: 10%; Caregiver White: 94%; Caregiver other ethnic background: 5.6% (Condition breakdown not stated).	Psych Distress: WEMWBS; Diabetes Distress: PAID‐PR	Blood Glucose: HbA1c (mmol/mol; Parent‐reported)
Katz et al., 2014; USA; Outpatient; Post‐int: 147 (condition breakdown not stated)	Description: Outreach and family‐focused psychoeducational intervention; Theory: Interdependence where adolescents and family members renegotiate and redistribute responsibilities; Facilitator: Care ambassador who was a research assistant Goal: To improve adherence to diabetes management; Topics: Family teamwork and communication; avoiding perfectionism and family conflict related to diabetes and weight gain; setting realistic goals; decreasing feelings of burnout; care coordination to enhance communication between families and clinic staff.	Length of time: 2 years; Amount: 30‐min session every 3 months and a monthly phone call; Child and Parent: Together; Delivery: Individual family, F2F and telephone.	Control 1: TAU; Control 2^c^: Active Control; Description: Monthly telephone outreach by a care ambassador in addition to quarterly visit‐based care coordination.	Age: Child Int: 12.7 (2.2); Child TAU: 12.5 (2.3); Child Active Con: 13.4 (2.4); Caregiver: Not stated. T1D duration: Child Int: 6.5 (3.8); Child TAU: 5.7 (3.5); Child Active Con: 6.8 (3.2)	Gender: Child Int: 58%; Child TAU: 45%; Child Active Con: 65%; Caregiver: Not stated Ethnicity: Child Int White: 90%; Child TAU White: 98%; Child Active Con White: 85%; Caregiver: Not stated.	Diabetes Family Conflict: DFCS‐R	Diabetes Family Conflict: DFCS‐R; Diabetes QoL: PedsQL‐Diabetes; Quality of Life: PedsQL‐Generic; Blood Glucose: HbA1c (%; Clinic reported)
Kichler et al., 2013; USA; Outpatient; Post‐int: 26 (13/13)	Description: Diabetes adjustment and coping group; Theory: Cognitive restructuring, behavioral change, motivation, and interpersonal skills; Facilitator: Licensed psychologist and a psychology student trainee; Goal: To improve psychosocial adjustment and diabetes management among adolescents and parents; Topics: Adolescent developmental aspects to diabetes management; Parent involvement and communication; goal setting and problem solving’ behavioral contingency and contracting; and school and peer issues.	Length of time: 6 weeks; Amount: 30‐45–min separate parent and child group followed by 20–30‐min sessions together, weekly; Child and Parent: Separately and then together; Delivery: Separate child and parent groups, followed by individual family sessions, F2F.	Control: WL with TAU; Description: Intervention offered 6 weeks after the intervention group.	Age: Child: 15.2 (1.3); Caregiver: Not stated (Condition breakdown not stated). T1D duration: Child mean age of T1D onset: 9.5 (3.2) (mean duration of T1D not stated) (Condition breakdown not stated).	Gender: Child: 53%; Caregiver: Not stated (Condition breakdown not stated). Ethnicity: Child White: 77%; Child African American: 20% (Condition breakdown not stated).	Psych Distress: BSI‐18	Diabetes QoL: PedsQL‐Diabetes; Quality of Life: PedsQL‐Generic; Blood Glucose: HbA1c (%; Clinic reported)
Laffel et al., 2003; USA; Outpatient; Post‐int: 100 (50/50)	Description: Family‐focussed teamwork intervention; Theory: Not stated; Facilitator: Trained research assistant; Goal: Minimized diabetes‐related family conflict, maintain quality of life, and increase adherence to glycaemic control; Topics: Communication around blood glucose results; meaning of ALC and parent–child teamwork; response to blood sugars; avoiding ‘blaming and shaming cycle’; using logbook to problem solve; and sharing burden of diabetes tasks with family members	Length of time: 12 months; Amount: 4 Sessions, every 3 months; Child and Parent: Together; Delivery: Individual family, F2F.	Control: WL with TAU; Description: Standard care, After the 1‐year study they were provided with teamwork intervention materials.	Age: Child Int: 11.9 (2.4); Child Con: 12.2 (2.2); Caregiver: Not stated. T1D duration: Child Int: 2.7 (1.6); Child Con: 2.7 (1.6)	Gender: Child Int: 56%; Child Con: 48%; Caregiver: Not stated. Ethnicity: Not stated.	Diabetes Family Conflict: DFCS	Diabetes Family Conflict: DFCS; Quality of Life: PedsQL‐Generic; Blood Glucose: HbA1c (%; Clinic reported)
Laffel et al., 2021; USA; Outpatient; Post‐int: 137 (48/46/43)	Description: Continuous glucose monitoring (CGM) with a family behavioral intervention (FBI); Theory: Not stated; Facilitator: Trained research assistant; Goal: To improve glycaemic outcomes and parent quality of life; Topics: Family feelings, attitudes, and behaviors that can be barriers to CGM; skills to manage behavioral barriers	Length of time: 6 months; Amount: nine sessions, four were 30 min of CGM training and five were 30 min of FBI training; Child and Parent: Together; Delivery: Individual family, F2F or telephone depending on participant preference.	Control 1: TAU; Control 2^c^: Active Con; Description: Standard care alongside CGM.	Age: Child Int: 5.7 (1.7); Child TAU: 6.2 (1.7); Child Active Con: 5.2 (1.8); Caregiver: Not stated. T1D duration: Child Int: 2.4 (1.9); Child Active Con: 1.9 (1.7); Child TAU: 2.6 (1.9)	Gender: Child Int: 58%; Child TAU: 53%; Child Active Con: 39%; Caregiver: Not stated. Ethnicity: Child Int Non‐Hispanic White: 65%; Child Int Non‐Hispanic Black: 20%; Child Int Hispanic or Latino: 10%; Child Int other: 4%; Child TAU Non‐Hispanic White: 63%; Child TAU Non‐Hispanic Black: 15%; Child TAU Hispanic or Latino: 13%; Child TAU Asian: 2%; Child TAU other: 8% Child Active Con Non‐Hispanic White: 77%; Child Active Con Non‐Hispanic Black: 9%; Child Active Con Hispanic or Latino: 12%; Child Active Con other: 2% Caregiver: Not Stated.	Diabetes Distress: PAID‐PR	Blood Glucose: HbA1c (% and mmol/mol; Clinic reported)
Lehmkuhl et al., 2010^h^; USA; Outpatient; Post‐int: 32 (18/14)	Description: Telehealth behavioral therapy; Theory: Not stated; Facilitator: Not stated; Goal: To increase adherence, decrease family discord, and decrease HBAIC. management; Topics: Problem solving, behavioral contracting, communication skills, cognitive restructuring, and family structure.	Length of time: 12 weeks; Amount: 15‐20–min sessions every 3 weeks; Child and Parent: Together; Delivery: Individual family, remote, telephone delivery.	Control 1: WL with TAU; Description: Standard care whilst on 1‐month W/L for intervention.	Age: Child Int: 13.7 (2.67); Child Con: 13.4 (2.2); Caregiver Int: 40.1 (8.3); Caregiver Con: 43.4 (7.9) T1D duration: Not stated.	Gender: Child Int: 61%; Child Con: 86%; Caregiver: 84.4% (Caregiver condition breakdown not stated) Ethnicity: Child Caucasian: 81.2%; Child African Amercian: 12%; Child Hispanic: 3.1%; Child Other: 3.7% Caregiver: Not stated (Condition breakdown not stated).	N/A	Blood Glucose: HbA1c (%; Clinic reported)^f^
Mackey et al., 2016; USA; Outpatient; 1 month FU^a^: 23 (12/11); 6 month FU: 21 (10/11); 12 month FU: 21 (9/12)	Description: Phone‐based intervention for parents; Theory: Not stated; Facilitator: Not stated; Goal: To improve glycaemic control and parental and child wellbeing; Topics: Positive thinking, problem solving, behavioral parenting strategies, social support, and parental selfcare.	Length of time: Not stated; Amount: 30‐min session every 3 months and a monthly phone call; Child and Parent: Parent only; Delivery: Individual, remote telephone delivery.	Control: Active; Description: 5 phone calls on physical activity and general child safety; Facilitator: BA level phone counselor; Topics covered: Diabetes and exercise safety, injury prevention, bicycle helmet use, and supervision on playgrounds.	Age: Child: 4.5 (1.7); Caregiver: 33.6 (6.7) (Condition breakdown not stated) T1D duration: 0.2 (0.1) (Condition breakdown not stated)	Gender: Child: 50%; Caregiver: 100% (Condition breakdown not stated) Ethnicity: Caregiver Caucasian: 70%; Child: Not stated (Condition breakdown not stated).	Psych Distress: CESD; Diabetes Distress: PIP	Quality of Life: PedsQL‐Generic; Blood Glucose: HbA1c (%; Clinic reported)
Mackey et al., 2022; USA; Outpatient; 3 Month FU^a^: Not reported; 6 Month FU Psych Distress only: 35 (18/17); 6 Month FU HbA1c only: 31.4 (14.4/17)	Description: Phone based intervention for parents providing behavioral strategies for glycaemic management, eating and activity; Theory: Not stated; Facilitator: Masters level clinical social worker, counselor, and certified diabetes care and education specialist; Goal: To increase protein and decrease carbohydrate intake at breakfast and increase physical activity; Topics: Managing child behavior, self‐monitoring, mealtime strategies, and developing consistent routines and habits.	Length of time: 9–12 weeks; Amount: Five, 40–60‐min phone sessions and one in person session for 90 min; Child and Parent: Parent only; Delivery: Individual, telephone delivery and F2F	Control: TAU	Age: Child: 4.2 (0.9); Caregiver: 36.3 (6.2) (Condition breakdown not stated) T1D duration: Not stated.	Gender: Child: 36%; Caregiver: Not stated (Condition breakdown not stated) Ethnicity: Child Caucasian: 74%; Child Black or African American: 22%; Child Asian: 6%; Caregiver: Not stated (Condition breakdown not stated).	Psych Distress: CESD	Blood Glucose: HbA1c (%; Clinic reported)
Majidi et al., 2021; USA; Outpatient; Post‐int: Assumed 86 (44/42)	Description: A shared medical appointment intervention for adolescents and caregivers. Adolescent groups included structured learning activities and group discussion. Parent groups involved group discussion. Adolescent and their parent session involved goal planning; Theory: Not stated; Facilitator: Nurses, dieticians, and social workers; Goal: To promote adolescent autonomy and diabetes behavior management to improve glycaemic control; Topics: Physical exercise, emotional information, and how diabetes can complicate adolescent development.	Length of time: 12 months; Amount: Four clinic visits, lasting 45–60 min; Child and Parent: Separately; Delivery: Separate child and parent groups, F2F.	Control: TAU	Age: Child Int: 12.0 (0.9); Child Con: 11.6 (0.8); Caregiver: Not stated T1D duration: Child Int: 4.5 (0.5); Child TAU: 4.8 (0.5)	Gender: Child Int: 45%; Child Con: 57%; Caregiver: Not stated Ethnicity: Child Int Black or African American: 5%; Child Int White: 71%; Child Int American Indian or Alaskan Native: 2.3%; Child Int more than one race: 11.4%; Child Int Unknown: 11.4%; Child Con Black or African American: 10%; Child Int White: 79%; Child Int American Indian or Alaskan Native: 0%; Child Int more than one race: 2.4%; Child Int Unknown: 10% Caregiver: Not stated	Diabetes Family Conflict: DFCS; Psych Distress: CESD	Diabetes Family Conflict: DFCS; Psych Distress: CDI‐2; Blood Glucose: HbA1c (%; Clinic reported)^f^
Mayer‐Davis et al., 2018; USA; Outpatient; Post‐int: 241 (118/123)	Description: Motivational interviewing and problem‐solving skills training to enhance self‐management; Theory: Health belief model, transtheoretical model, and theory of reasoned action; Facilitator: Nurse educators trained in MI and PSST and dietician; Goal: To increase adherence to T1D self‐management; Topics: Family communications and teamwork; diabetes educations relevant to behavioral goal attainment; and participant defined reminders.	Length of time: 18 months; Amount: Four, 40–60 min sessions in the first 12 weeks. 3–4 sessions for each subsequent 6‐month period; Child and Parent: Child only, with parents involved in some sessions; Delivery: Individual, F2F.	Control: TAU	Age: Child Int: 14.8 (1.1); Child Con: 14.9 (1.1); Caregiver: Not stated T1D duration: Child Int: 6.5 (3.8); Child TAU: 6.4 (3.7)	Gender: Child Int: 45%; Child Con: 54%; Caregiver: 84% (Caregiver condition breakdown not stated). Ethnicity: Child Int Non‐Hispanic White: 77%; Child Int Black: 5%; Child Int Hispanic: 12%; Child Int other: 5%; Child Con Non‐Hispanic White: 78%; Child Con Black: 3%; Child Con Hispanic: 13%; Child Con other: 6%; Caregiver: Not stated	Diabetes Family Conflict: DFCS	Diabetes Family Conflict: DFCS; Psych Distress: CESD; Quality of Life: PedsQL‐Generic; Blood Glucose: HbA1c (% and mmol/mol; Clinic reported)
Mitchell et al., 2022; USA; Community; Post‐int: 42 (17/25); 6 month FU: 39 (15/24)	Description: Parent group sessions and discussion targeting managing child behavior; Theory: Social learning theory; Facilitator: Not stated; Goal: To examine the efficacy of brief group‐based parenting in improving outcomes for families of children with T1D; Topics: Session 1—strategies to prevent and manage problem behaviors and promote parent–child cooperation and management. Session 2—principles of positive parenting in the chronic illness context, increased risk of behavioral problems, common parenting traps, and effective parenting strategies for asserting discipline.	Length of time: 1 week; Amount: 2, 60‐min sessions; Child and Parent: Parent only; Delivery: Groups, F2F.	Control: TAU	Age: Child Int: 7.1 (2.1); Child Con: 6.4 (2.5); Caregiver Int: 39.6 (6.1); Caregiver Con: 40.3 (5.4) T1D duration: Child Int mean age of T1D onset: 4.8 (2.5) Child Con mean age of T1D onset: 4.6 (3.1) (mean duration of T1D not stated).	Gender: Child Int: 73%; Child Con: 72%; Caregiver Int: 96%; Caregiver Con: 86% Ethnicity: Australian: 58%; European: 22% (Child and Caregiver breakdown not stated; condition breakdown not stated).	N/A	Quality of Life: PedsQL‐Generic; Blood Glucose: HbA1c (%; Clinic reported)
Monaghan et al., 2011; USA; Outpatient; Post‐int: 19 (10/9)	Description: Sessions of supporting parents intervention involving skills training; Theory: Social cognitive theory; Facilitator: Not stated; Goal: To decrease parental anxiety, depression and stress, and increase social support; Topics: Skills training such as problem solving and encouraging use of cognitive behavioral coping strategies.	Length of time: Not stated; Amount: 5 sessions; Child and Parent: Parent only; Delivery: Not stated.	Control: WL with TAU; Description: Continued with standard care whilst on waitlist.	Age: Child: 4.1 (0.8); Caregiver: 34.8 (6.2) (Condition breakdown not stated). T1D duration: 2.1 (0.6) (Condition breakdown not stated)	Gender: Child: 50%; Caregiver: 88% (Condition breakdown not stated). Ethnicity: Child Caucasian: 75%; Caregiver: Not stated. (Condition breakdown not stated).	Psych Distress: CESD; Diabetes Distress: PIP	N/A
Murphy et al., 2007^h^; UK; Outpatient; Post‐int: 33 (condition breakdown not stated)	Description: Family group educational sessions; Theory: Educational and social learning theory; Facilitator: Dietician, pediatric nurse specialist, physician, and a diabetes nurse specialist with counseling experience. All further trained in delivery of group education; Goal: To enhance parental responsibility for self‐management and improve glycaemic control; Topics: two skills‐based sessions on carbohydrate counting and insulin dose adjustment. Two sessions on parental responsibility and communication.	Length of time: 12 months; Amount: Four, 60‐min sessions every 3 months; Child and Parent: Together; Delivery: F2F, with groups of families.	Control: WL with TAU; Description: Standard care for 12 months prior to joining intervention.	Age: Child Int: 12.6 (2.3); Child Con: 13.1 (2.0); Caregiver: Not stated T1D duration: Child Int: 5.1 (3.5); Child Con: 4.7 (3.0)	Gender: Child Int: 45%; Child Con: 44%; Caregiver: Not stated Ethnicity: Child: Not stated; Caregiver: Not stated	N/A	Diabetes Distress: PAID^f^; Diabetes QoL: PedsQL‐Diabetes^f^; Blood Glucose: HbA1c (%; Clinic reported)^f^
Murphy et al., 2012; UK; Outpatient; 3 month FU HbA1c: 205 (105/100); 6 month FU psychometrics: 205 (105/100); 12 month FU HbA1c: 295 (154/141)	Description: Family centred group education programme; Theory: Social learning theory; Facilitator: Multidisciplinary health professionals who had completed programme specific training; Goal: To enhance parental responsibility for self‐management and improve glycaemic control; Topics: Carbohydrate counting, family communication about food and blood glucose monitory, family problem solving, learning from mistakes, and interdependence.	Length of time: 6 months; Amount: Six, 90‐min sessions, every month. Child and Parent: Together; Delivery: F2F, with groups of families.	Control: TAU	Age: Child Int: 13.1 (1.9); Child Con: 13.2 (2.0); Caregiver: Not stated. T1D duration: Child Int: 5.5 (3.1); Child TAU: 5.6 (3.4)	Gender: Child Int: 53%; Child Con: 51%; Caregiver: Not stated. Ethnicity: Child Int White: 93%; Child Con White: 91%; Caregiver: Not stated.		Diabetes Qol: DQOL‐SF‐Impact; Diabetes Distress: DQOLY‐SF‐Worry; Blood Glucose: HbA1c (% and mmol/mol; Clinic reported)
Nansel et al., 2009; USA; Outpatient; Post‐int: 116 (58/58)	Description: “We can manage diabetes” intervention which included telephone calls and in person contact. Content structured by the WE‐CAN problem solving approach and families collaborating to identify a goal for management and behavior plan; Theory: Social cognitive theory, self‐regulation models, and system theory; Facilitator: Health advisors who were training college graduates in diabetes management; Goal: To help families improve diabetes management; Topics: Problem solving, goal setting, communication skills, and appropriate responsibility sharing.	Length of time: 11 months; Amount: Three clinic appointments every 3 months. Two follow up phone calls 2–8 weeks after each clinic; Child and Parent: Together; Delivery: Individual family F2F, with remote follow up telephone call.	Control: WL with TAU; Description: Standard care until completion of the study. Then families received intervention materials and a summary of the problem‐solving process.	Age: Child: 11.5; Caregiver: Not stated. (SD and condition breakdown not stated) T1D duration: Child mean age of T1D onset: 6.7 (Condition breakdown and SD not stated; mean duration of T1D not stated).	Gender: Child: Not stated. Caregiver: Not stated. Ethnicity: Child White: 71%; Child Black and other: 7%; Child Hispanic: 12%; Caregiver: Not stated (Condition breakdown not stated).	Diabetes Family Conflict: DFCS‐R	Diabetes Family Conflict: DFCS‐R; Diabetes QoL: PedsQL‐Diabetes; Quality of Life: PedsQL‐Generic; Blood Glucose: HbA1c (%; Clinic reported)
Nansel et al., 2012; USA; Outpatient; 3 Month FU: 360 (182/178)	Description: Family‐based behavioral intervention; Theory: Self‐regulation perspective, social cognitive theory, and self‐determination theory; Facilitator: Research assistants training in pediatric T1D, intervention procedures, and motivational interviewing; Goal: To help families improve diabetes management by facilitating problem‐solving skills, communication skills, and appropriate responsibility sharing; Topics: Interaction education, learning activities, goal setting, and application of the problem‐solving process to increase intake of healthy foods.	Length of time: 21 months; Amount: Three clinic appointments, every 3–4 months, with 2 follow up phone calls 2–8 weeks after each clinic; Child and Parent: Together; Delivery: F2F, with individual family, and follow up telephone calls.	Control: WL with TAU; Description: Standard care until completion of the study. Then families received a notebook with the intervention materials.	Age: Child Int: 12.5 (1.8); Child Con: 12.4 (1.7); Caregiver: Not stated. T1D duration: Child Int: 4.8 (3.3); Child TAU: 4.9 (3.2)	Gender: Child Int: 51%; Child Con: 51%; Caregiver: Not stated. Ethnicity: Child Int White: 76%; Child Int Black: 8%; Child Int Hispanic: 11%; Child Con White: 75%; Child Con Hispanic: 9%; Child Con Black: 11%; Caregiver: Not stated.	N/A	Blood Glucose: HbA1c (%; Clinic reported)
Nansel et al., 2015; USA; Outpatient; 11 Month FU: 125 (55/70)	Description: “We can manage diabetes” intervention which included telephone calls and in person contact. Content structured by the WE‐CAN problem solving approach and families collaborating to identify a goal for management and behavior plan; Theory: Social cognitive theory, self‐regulation models, and system theory; Facilitator: Health advisors who were training college graduates in diabetes management; Goal: To help families improve dietary intake and glycaemic control; Topics: Problem solving, goal setting, communication skills	Length of time: 7 months; Amount: Six sessions. Child and Parent: Together; Delivery: F2F with individual family, with follow up telephone call.	Control: Active; Description: Received equal frequency contacts with research staff focused on case management within the diabetes health care system. Equal frequency of 3‐day masker continuous glucose monitoring use. No dietary advice beyond standard care.	Age: Child Int: 12.6 (2.7); Child Con: 13.0 (2.5); Caregiver: Not stated. T1D duration: Child Int: 5.6 (2.5); Child TAU: 6.3 (3.6)	Gender: Child Int: 47%; Child Con: 56%; Caregiver: Not stated. Ethnicity: Child Int White: 88%; Child Int Black: 3%; Child Int Hispanic: 9%; Child Con White: 93%; Child Con Hispanic: 1%; Child Con Black: 4%; Caregiver: Not stated.	N/A	Blood Glucose: HbA1c (%; Clinic reported)
Patton et al., 2020; USA; Outpatient; Post‐int: 36 (18/18); 3 month FU^i^: 36	Description: A parental telehealth intervention; Theory: Cognitive behavioral therapy; Facilitator: Clinical psychology doctoral students and supervised by a licensed clinical psychologist and certified diabetes educator; Goal: To reduce hypoglycaemia fear, maladaptive avoidance behaviors, promote healthy coping, and reduce distress in parents; Topics: Personalized fear hierarchy of T1D‐related situations, coping strategies, imagined and in vivo exposures to challenge maladaptive thoughts/behaviors, support from other parents and group leaders, review T1D management skills, behavioral parenting strategies.	Length of time: 10 weeks; Amount: Ten 30–60‐min sessions, weekly; Child and Parent: Parent only; Delivery: Remote, mix of group and individual parent sessions.	Control: WL with TAU; Description: Standard care after 14 weeks completed the intervention.	Age: Child: 4.4 (1.4); Caregiver: 36.0 (4.9) (Condition breakdown not stated). T1D duration: Child mean age of T1D onset: 2.4 (1.2) (Condition breakdown not stated; mean duration of T1D not stated).	Gender: Child: 61%; Caregiver: 97% (Condition breakdown not stated). Ethnicity: Child White: 95%; Caregiver: Not stated. (Condition breakdown not stated)	Diabetes Distress: PAID‐PR	Blood Glucose: HbA1c (%; Clinic reported)
Saßmann et al., 2012; Germany; Outpatient; 3 Month FU: 57 (34/23)	Description: Structured parent group training in a group of up to 7 families; Theory: Behavioral theory; Facilitator: Experienced Psychologist; Goal: To strengthen parents' general and diabetes specific education competences; Topics: Processing of dysfunctional cognitions, communication skills, and handling conflict.	Length of time: 6 weeks; Amount: Weekly, two‐hour sessions, for 5 weeks. Then a follow up telephone session; Child and Parent: Parent only; Delivery: F2F with groups of parents.	Control: WL with TAU; Description: Standard care and then accessed intervention after 3‐month study period.	Age: Child Int: 6.4 (2.3); Child Con: 5.8 (1.9); Mothers Int: 39.1 (3.3); Mothers Con: 40.4 (3.8); Fathers Int: 43.3 (5.5); Fathers Con: 42.4 (6.5) T1D duration: Child Int: 2.6 (1.6); Child TAU: 2.6 (1.9)	Gender: Child: Not stated; Caregiver: 51% (Condition breakdown not stated) Ethnicity: Child: Not stated; Caregiver: Not stated	Psych Distress: DASS‐42	Blood Glucose: HbA1c (%)
Saghaei et al., 2017; Iran; Not stated; Post‐int: 50 (25/25)	Description: Education stress management sessions for mothers; Theory: Cognitive behavioral therapy; Facilitator: Psychotherapists; Goal: To improve mothers mental health; Topics: Effects of stress on blood glucose, self‐relaxation and diaphragmatic breathing; relationship between thinking and emotion; challenging negative cognitions; anger management; problem solving; communication; and self‐presentation.	Length of time: 2 months; Amount: Eight, 2‐h sessions, weekly; Child and Parent: Parent only; Delivery: F2F, individual.	Control: TAU	Age: Child Int: 9.3 (2.6); Child Con: 9.1 (1.9); Caregiver: Not stated. T1D duration: Not stated.	Gender: Child Int: 72%; Child Con: 64%; Caregiver: 100% (Caregiver condition breakdown not stated). Ethnicity: Child: Not stated Caregiver: Not stated	Psych Distress: DASS‐42	Blood Glucose: HbA1c (% assumed; Clinic Reported)
Sairanen et al., 2019; Sweden; Outpatient; Post‐int: 26 (14/12); 4 month FU: 24 (12/12)	Description: A web‐based acceptance and commitment therapy for parents; Theory: Acceptance and commitment therapy; Facilitator: A personal coach who was an undergraduate psychology student who had undergone training in ACT; Goal: To teach parents skills and strategies to prevent and handle stress and worries in everyday life; Topics: Life values, present moment, diffusion, acceptance, and self‐compassion.	Length of time: 10 weeks; Amount: Five online modules. Child and Parent: Parent only; Delivery: Remote, Individual parent.	Control: WL with TAU; Description: Standard care and after 4 months granted access to the online intervention.	Age: Caregiver Int: 42.3 (7.4); Caregiver Con: 43.0 (6.0); Child: Not stated T1D duration: Not stated	Gender: Child Int: 53%; Child Con: 35%; Caregiver Int: 90%; Caregiver Con: 77% Ethnicity: Child: Not stated; Caregiver: Not stated	Psych Distress: DASS‐21‐Depression	N/A
Sullivan‐Bolyai et al., 2004; USA; Outpatient; Post‐int: 41 (22/19)	Description: Social support intervention for mothers; Theory: Irey's social support model and the emotional support component of Brooten's quality‐cost model; Facilitator: Parent mentors trained in Irey's curriculum; Goal: To increase mothers' confidence in T1D management, reduce concerns and worry, increase mothers' perceived involvement in their child's day‐to‐day T1D management; Topics: Three types of social support: practical and information; affirmational; and emotional support.	Length of time: 6 months; Amount: A baseline home visit by the mentor and a negotiated number of home visits and phone calls; Child and Parent: Parent only; Delivery: F2F and remote (telephone calls, and emails), individual.	Control: WL with TAU; Description: Standard care, after the intervention mothers were offered access to the parent mentors.	Age: Caregiver 35.0 (6.0); Child: 5.0 (3.0) (Condition breakdown not stated) (SD not stated) T1D duration: Not stated.	Gender: Child: 33%; Caregiver: 100% (Condition breakdown not stated) Ethnicity: Caregiver White: 90%; Caregiver African American: 2%; Caregiver Hispanic: 5%; Child: Not stated (Condition breakdown not stated)	Psych Distress: BDMCQ	N/A
Sullivan‐Bolyai et al., 2010; USA; Outpatient; Post‐int: 51 (30/21)	Description: Social support intervention for parents; Theory: Irey's social support model; Facilitator: Parent mentors trained in Irey's curriculum; Goal: To reduce parents' concern and worry, increase confidence, perceive less negative impact of the illness on the family, and more readily identify resources and support within their community; Topics: Three types of social support: practical and information; affirmational; and emotional support.	Length of time: 12 months; Amount: Negotiated frequency by mother and mentor; Child and Parent: Parent only; Delivery: F2F and remote (telephone calls, and emails), individual.	Control: Active; Description: Access to a parent contact. This individual had no structured formal mentor training.	Age: Caregiver: 36.0 (6.0); Child: 6.0 (2.86) (Condition breakdown not stated) T1D duration: Not stated.	Gender: Child: 45% (Condition breakdown not stated); Caregiver: 100% Ethnicity: Caregiver Int White: 87%; Caregiver Int Black/Latino/Other: 14%; Caregiver Con White: 89%; Caregiver Con Black/Latino/Other: 12%	Diabetes Psych Distress: WS	N/A
Sullivan‐Bolyai et al., 2011; USA; Outpatient; Post‐int: 22 (16/6)	Description: Social support intervention for fathers; Theory: Irey's social support model; Facilitator: Parent mentors trained in Irey's curriculum; Goal: To increase fathers' confidence in T1D management, reduce concerns and worry, increase fathers' perceived involvement in their child's day‐to‐day T1D management; Topics: Three types of social support: practical and information; affirmational; and emotional support.	Length of time: 12 months; Amount: Negotiated frequency by father and mentor; Child and Parent: Parent only; Delivery: F2F and remote (telephone calls, and emails), individual.	Control: Active; Description: Access to a parent contact. This individual had no structured formal mentor training.	Age: Caregiver Int: 38.0; Caregiver Con: 39.0; Child Int: 5.0; Child Con: 6.0 (SD not stated) T1D duration: Not stated.	Gender: Child: 39%; Caregiver: 0% (Condition breakdown not stated) Ethnicity: Caregiver Int White: 95%; Caregiver Int Latino: 5%; Caregiver Con White: 100%; Child: Not stated	Diabetes Psych Distress: WS	N/A
Sullivan‐Bolyai et al., 2016; USA; Outpatient; 2‐week FU: 22 (11/11)	Description: Diabetes self‐management education and peer support; Theory: Revised Family management style framework; Facilitator: Teen mentors, parent mentors, and nurses; Goal: To increase family self‐efficacy, problem solving, and collaborative decision making to improve glucose control and family functioning, so it becomes more family, and less condition, focused; Topics: Education, problem solving, collaboration, decision making, social support, management of behaviors, communication and negotiation.	Length of time: 1 day; Amount: 60–90‐min session; Child and Parent: Parent and child together, and parent separately; Delivery: F2F, Individual families and parent separately.	Control: Active; Description: Diabetes education only. Parents and children discussed hypoglycaemia management with a nurse for 60–90 min.	Age: Caregiver Int: 43.0 (8.0); Caregiver Con: 44.0 (6.0); Child Int: 11.0 (1.0); Child Con: 11.0 (2.0) T1D duration: Child Int: 3.0 (2.0); Child TAU: 4.0 (3.0)	Gender: Child Int: 64%; Child Con: 36%; Caregiver Int: 100% Caregiver Con: 82% Ethnicity: Caregiver Int White: 73%; Caregiver Int Black: 9%; Caregiver Int Latino: 18%; Caregiver Con White: 82%; Caregiver Con Black: 18%; Child: Not stated.	Psych Distress: HFS	N/A
Tsiouli et al., 2014; Greece; Outpatient Post‐int: 44 (19/25)	Description: Stress management programme; Theory: Not stated, but intervention drew on psychological evidence and positive health behaviors; Facilitator: First author who was on postgraduate course stress management and health promotion course; Goal: To reduce perceived and parenting stress, increase internal locus of control, promote healthy lifestyle and normalizing cortisol levels in parents; Topics: progressive muscle relaxation, diaphragmatic breathing, information given on the connection between stress and lifestyle and instructed to adapt a healthy lifestyle.	Length of time: 8 weeks; Amount: One 37 min individual sessions followed by self‐practice twice a day; Child and Parent: Parent only; Delivery: Initial F2F individual sessions followed by self‐directed practice using CD.	Control: Active; Description: Healthy way of living training, instructed to adopt a healthy lifestyle, given educational pamphlets on healthy lifestyle practices.	Age: Child: Not stated; Caregiver Int: 44.0; Caregiver Con: 42.6 (SD not stated) T1D duration: Not stated	Gender: Child: Not sated; Caregiver Int: 79%; Caregiver Con: 80%	Psych Distress: PSS	N/A
Van Name et al., 2023 USA; Outpatient; Post‐int: 137 (48/46/43) 6 month FU: (45/44/42)	Description: Continuous glucose monitoring (CGM) with a family behavioral intervention (FBI); Theory: Not stated; Facilitator: Trained research assistant; Goal: To improve glycaemic outcomes and parent quality of life; Topics: Family feelings, attitudes, and behaviors that can be barriers to CGM; skills to manage behavioral barriers	Length of time: 6 months; Amount: nine sessions, four were 30 min of CGM training and five were 30 min of FBI training; Child and Parent: Together; Delivery: Individual family, F2F or telephone depending on participant preference.	Control 1: TAU for 6 months, then Active Control of standard care alongside CGM for 6 months; Control 2^c^: Active Con; Description: Standard care alongside CGM.	Age: Child Int: 5.7 (1.6); Child TAU: 6.1 (1.7); Child Active Con: 5.1 (1.8); Caregiver: Not stated. T1D duration: Child Int: 2.4 (1.9); Child Active Con: 1.8 (1.7); Child TAU: 2.7 (2.0)	Gender: Child Int: 58%; Child TAU: 50%; Child Active Con: 40%; Caregiver: Not stated. Ethnicity: Child Int Non‐Hispanic White: 68%; Child Int Non‐Hispanic Black: 16%; Child Int Hispanic or Latino: 11%; Child Int other: 5%; Child TAU then Active Con Non‐Hispanic White: 63%; Child TAU then Active Con Non‐Hispanic Black: 14%; Child TAU then Active Con Hispanic or Latino: 12%; Child TAU then Active Con Asian: 2%; Child TAU then Active Con other: 9% Child Active Con Non‐Hispanic White: 76%; Child Active Con Non‐Hispanic Black: 10%; Child Active Con Hispanic or Latino: 12%; Child Active Con other: 2% Caregiver: Not Stated.	N/A	Blood Glucose: HbA1c (mmol/L; Clinic reported)^b^ (Post‐int excluded as reported in Laffel et al., 2021)
Westrupp et al., 2015; Australia; Outpatient; Post‐int: 60 (29/31); 6 month FU^g^: 44 (24/20); 9 month FU: 57 (32/25)	Description: Triple P—Positive Parenting Programme; Theory: Social learning, cognitive behavioral, and developmental theory; Facilitator: Clinical Psychologist; Goal: To reduce parental mental health problems and family conflict. To improve glycaemic control, parenting skills, and family functioning; Topics: 17 Parenting strategies—10 to promote children's competence and development, and 7 to help parents manage misbehavior.	Length of time: 3‐months; Amount: Ten, 1‐h sessions, weekly; Child and Parent: Parent only; Delivery: F2F, Individual.	Control: TAU	Age: Child Int: 9.0; Child Con: 9.0; Caregiver: Not stated (SD not stated, condition breakdown not stated) T1D duration: Not stated	Gender: Child Int: 37%; Child Con: 50%; Caregiver: Not stated Ethnicity: Child: Not stated Caregiver: Not stated	Diabetes Family Conflict: DFCS‐R Psych Distress: DASS‐42‐Depression	Blood Glucose: HbA1c (% and mmol/mol; Clinic reported)
Whittemore et al., 2020^h^; USA; Outpatient; Post‐int: 133 (60/73); 3 month FU: 127 (55/72)	Description: eHealth program to reduce parenting stress around T1D management; Theory: Transactional model of stress and coping; Facilitator: Self‐directed; Goal: To reduce parenting stress and increase safe transfer of responsibility and positive communication; Topics: Information on the challenges of adolescence, developing a positive partnership between parents and adolescents, communicating positively and transferring responsibility to adolescents gradually, understanding parents' emotions and the importance of taking care of oneself.	Length of time: 3 months; Amount: 6 online sessions; Child and Parent: Parent only; Delivery: F2F, individual.	Control: WL with TAU; Description: Standard care and then given access to type 1 teamwork website once the study period ended.	Age: Child Int: 13.9 (1.7); Child Con: 13.9 (1.6); Caregiver Int: 45.4 (5.1); Caregiver Con: 35.0 (6.0); T1D duration: 5.1 (3.6) (Condition breakdown not stated)	Gender: Child Int: 51%; Child Con: 56%; Caregiver Int: 99%; Caregiver Con: 96% Ethnicity: Child Int White: 91%; Child Int Asian: 1%; Child Con White: 90%; Child Con Black 2.5%; Caregiver White: 5%; (Caregiver condition breakdown not stated)	Psych Distress: PSS^f^; Diabetes Family Conflict: DFCS^f^	Blood Glucose: HbA1c (% and mmol/mol; parent‐reported)^f^
Wysocki et al., 2000 USA; Outpatient; Post‐int: 115 (35/41/39)	Description: Behavioral family systems therapy; Theory: Not stated; Facilitator: Two licensed Psychologists; Goal: To reduce diabetes‐related family conflict, and improvement treatment adherence and metabolic control; Topics: Problem‐solving and communication skills training, cognitive restructuring, and functional and structural family therapy.	Length of time: 3 months; Amount: 10 sessions; Child and Parent: Together; Delivery: F2F, individual families.	Control 1: TAU; Description: Standard care. Control 2^c^: Active; Description: Standard care and families attended 10 group meetings within 3 months for education and social support. This included a 45‐min educational presentation and 45‐min family interaction. Facilitator: Master's level social worker and master's level health educator.	Age: Child Int: 14.5 (1.2); Child TAU: 14.3 (1.4); Child Active Con: 14.1 (1.4); Caregiver: Not stated T1D duration: Child Int: 5.4 (3.8); Child TAU: 5.2 (3.8); Child Active Con: 4.5 (3.7)	Gender: Child Int: 61%; Child TAU: 51%; Child Active Con: 62%; Caregiver: Not stated; Ethnicity: Child Int Caucasian: 79%; Child Int African American: 21%; Child TAU Caucasian: 78%; Child TAU African American: 22%; Child Active Con Caucasian: 80%; Child Active Con African American: 17%; Child Active Con Hispanic: 3%; Caregiver: Not stated	Family Conflict: IC^e^, ^f^	Family Conflict: IC^e^, ^f^; Diabetes Family Conflict: DFCS^f^; Blood Glucose: HbA1C (%; Clinic reported)
Wysocki et al., 2001^h^; USA; Unclear; Post‐int: 115 (37/40/38); 6 month FU: 113 (36/40/37); 12 month FU: 108 (34/38/36)	Description: Behavioral‐family systems therapy; Theory: Not stated; Facilitator: A woman and man trained in behavioral‐family systems therapy; Goal: To improve family relationships, increase treatment adherence, and improve adjustment to diabetes; Topics: Problem‐solving and communication skills training, cognitive restructuring, and functional and structural family therapy.	Length of time: Not stated; Amount: 10 sessions; Child and Parent: Together; Delivery: Not stated.	Control 1: TAU; Description: Standard care. Control 2^c^: Active; Description: 10 group meetings with parents and children. This included a 45‐min educational presentation and 45‐min family interaction. Facilitator: Social worker or health educator.	Age: Child: 14.3 (1.3); Caregiver: Not stated (Condition breakdown not stated) T1D duration: 5.2 (3.7) (Condition breakdown not stated)	Gender: Child: 57%; Caregiver: Not stated (Condition breakdown not stated) Ethnicity: Child Caucasian: 79%; Child African American: 20%; Child Hispanic: 1%; Caregiver: Not stated (Condition breakdown not stated)	Family Conflict: PARQ‐Global Distress^e^, ^f^; Diabetes Family Conflict: DFCS‐conflict^f^	Family Conflict: PARQ‐Global distress^e^, ^f^; Diabetes Family Conflict: DFCS‐ conflict^f^; Blood Glucose: HbA1c (%; Clinic reported)^f^
Wysocki et al., 2006^h^; USA; Outpatient; Post‐int: 92 (28/29/35)	Description: Behavioral family systems therapy; Theory: Not stated; Facilitator: Psychologist or licensed clinical social worker; Goal: To reduce diabetes‐related family conflict, and improvement treatment adherence and metabolic control; Topics: Problem‐solving and communication skills training, cognitive restructuring, and functional and structural family therapy.	Length of time: 6 months; Amount: 12 sessions; Child and Parent: Together; Delivery: Not stated.	Control 1: TAU; Description: Standard care. Control 2^c^: Active; Description: Standard care and families attended 12 multifamily meetings within 6 months for education and social support. This included a 45‐min educational presentation and 45‐min family interaction. Facilitator: Experienced diabetes nurses.	Age: Child Int: 13.9 (1.9); Child TAU: 14.2 (1.9); Child Active Con: 14.4 (1.9); Caregiver: Not stated T1D duration: Child Int: 5.1 (3.0); Child TAU: 5.9 (4.0); Child Active Con: 5.5 (3.2)	Gender: Child Int: 42%; Child TAU: 50%; Child Active Con: 44%; Caregiver: Not stated; Ethnicity: Child Int Caucasian: 61%; Child Int African American: 33%; Child Int Hispanic: 3%; Child Int Other: 3%; Child TAU Caucasian: 53%; Child TAU African American: 34%; Child TAU Hispanic: 6%; Child TAU Other: 6%; Child Active Con Caucasian: 75%; Child Active Con African American: 25%; Caregiver: Not stated	Family Conflict: PARQ‐Global Distress^e^, ^f^; Diabetes Family Conflict: DFCS^f^	Family Conflict: PARQ‐Global distress^e^, ^f^; Diabetes Family Conflict: DFCS^f^; Blood Glucose: HbA1C (%; Clinic reported)^f^
Wysocki et al., 2007; USA; Outpatient Post‐int: 92 (36/32/36); 3 month FU^g^: Not stated; 6 month FU^g^: Not stated; 9 month FU^g^: Not stated; 12 month FU^g^: 85 (28/26/31)	Description: Behavioral family systems therapy; Theory: Not stated; Facilitator: Psychologist or licensed clinical social worker; Goal: To reduce diabetes‐related family conflict, and improvement treatment adherence and metabolic control; Topics: Problem‐solving and communication skills training, cognitive restructuring, and functional and structural family therapy.	Length of time: 6 months; Amount: Not stated; Child and Parent: Together; Delivery: Not stated.	Control 1: TAU; Description: Standard care. Control 2^c^: Active; Description: Standard care and families attended 12 multifamily meetings within 6 months for education and social support. This included a 45‐min educational presentation and 45‐min family interaction. Facilitator: Experienced diabetes nurses.	Age: Child Int: 13.9 (1.9); Child TAU: 14.2 (1.9); Child Active Con: 14.4 (1.9); Caregiver: Not stated T1D duration: Child Int: 5.1 (3.0); Child TAU: 5.9 (4.0); Child Active Con: 5.5 (3.2)	Gender: Child Int: 42%; Child TAU: 50%; Child Active Con: 44%; Caregiver: Not stated; Ethnicity: Child Int Caucasian: 61%; Child Int African American: 33%; Child Int Hispanic: 1%; Child Int Other: 1%; Child TAU Caucasian: 53%; Child TAU African American: 34%; Child TAU Hispanic: 6%; Child TAU Other: 6%; Child Active Con Caucasian: 75%; Child Active Con African American: 25%; Caregiver: Not stated	Diabetes Family Conflict: DFCS^f^	Diabetes Family Conflict: DFCS^f^; Blood Glucose: HbA1c (%; Clinic reported)
Wysocki et al., 2008^h^; USA; Outpatient; Post‐int: 92 (28/29/35); 6 month FU: Not reported; 12 month FU: 85 (28/26/31)	Description: Behavioral family systems therapy; Theory: Not stated; Facilitator: Psychologist or supervised postdoctoral fellow; Goal: To reduce diabetes‐related family conflict, and improvement treatment adherence and metabolic control; Topics: Problem‐solving and communication skills training, cognitive restructuring, and functional and structural family therapy.	Length of time: 6 months; Amount: 12 sessions; Child and Parent: Together; Delivery: F2F, Individual families.	Control 1: TAU; Description: Standard care. Control 2^c^: Active; Description: Standard care and families attended 12 multifamily meetings within 6 months for education and social support. This included a 45‐min educational presentation and 45‐min family interaction. Facilitator: Experienced diabetes nurses.	Age: Child Int: 13.9 (1.9); Child TAU: 14.2 (1.9); Child Active Con: 14.4 (1.9); Caregiver: Not stated T1D duration: Child Int: 5.1 (3.0); Child TAU: 5.9 (4.0); Child Active Con: 5.5 (3.2)	Gender: Child Int: 42%; Child TAU: 50%; Child Active Con: 44%; Caregiver: Not stated; Ethnicity: Child Int Caucasian: 61%; Child Int African American: 33%; Child Int Hispanic: 3%; Child Int Other: 3%; Child TAU Caucasian: 53%; Child TAU African American: 34%; Child TAU Hispanic: 6%; Child TAU Other: 6%; Child Active Con Caucasian: 75%; Child Active Con African American: 25%; Caregiver: Not stated	Family Conflict: DFCS^e^, ^f^	Diabetes Family Conflict: DFCS^f^; Blood Glucose: HbA1C (%; Clinic reported)^f^

*Note:* All interventions and active controls were delivered in addition to routine medical care for T1D (treatment as usual).

Abbreviations: BDMCQ, Banion Diabetes Management Concern Questionnaire (Ireys et al., 1999); BSI‐18, The Brief Symptom Inventory (Derogatis, 1993); CDI, Child Depression Inventory (Kovacs, 1985); CDI‐2, Child Depression Inventory 2 (Bae, 2012); CESD, The Center for Epidemiologic Studies Depression Scale (Radloff, 1977); Con, Control group; DASS‐21‐Depression, Depression Anxiety Stress Scales (21 items, Depression subscale) (Lovibond, 1995); DASS‐42, Depression Anxiety Stress Scales (42 items) (Lovibond, 1995); DASS‐42‐Depression, Depression Anxiety Stress Scales (42 items, Depression subscale) (Lovibond, 1995); DDS‐P, Diabetes Distress Scale (Polonsky et al., 2005); DFCS, Diabetes Family Conflict Scale (Rubin et al., 1989); DFCS‐Conflict, Diabetes Family Conflict Scale (Conflict subscale) (Rubin et al., 1989); DFCS‐R, Diabetes Family Conflict Scale Revised (Hood et al., 2007); DQOL‐Y, Diabetes Quality of Life Scale for Youth (Ingersoll et al., 1991); DQOL‐Y‐Satisfaction, Diabetes Quality of Life Scale for Youth (Satisfaction Subscale) (Ingersoll et al., 1991); DQOLY‐SF‐Impact, Diabetes Quality of Life Scale for Youth (Short Form Impact Subscale) (Skinner et al., 2006); DQOLY‐SF‐Worry, Diabetes Quality of Life Scale for Youth (Short Form Worry Subscale) (Skinner et al., 2006); DSQ, Diabetes Stress Questionnaire (Boardway et al., 1993); F2f, Face to face; FES‐Conflict, Family Environment Scale (Conflict Subscale) (Moos et al., 2002); FU, Follow up; HbA1c, hemoglobin A1C; HbA1c%, hemoglobin A1c percentage; HbA1c mmol/mol, hemoglobin A1C millimole/molemol; HFS, Parents Fear Hypoglycaemia Fear Scale (Adaption of worry subscale) (Clarke et al., 1998); IC, Issues Checklist (Prinz et al., 1979); Int, Intervention group; Issues Coping IDDM, Issues in Coping with insulin‐dependent diabetes mellitus (Kovacs et al., 1986); Issues Coping IDDM‐Upset, Issues in Coping with insulin‐dependent diabetes mellitus (Upset subscale) (Kovacs et al., 1986); MY‐Q, Monitoring Individual Needs in Diabetes Youth Questionnaire (de Wit et al., 2012); PAID, Problem Areas in Diabetes Scale (Polonsky et al., 1995); PAID‐Modified, Problem Areas in Diabetes Scale (Modified Version) (Polonsky et al., 1995); PAID‐PR, Problem Areas in Diabetes Scale (Parent Revised Version) (Markowitz et al., 2012); PAID‐T, Problem Areas in Diabetes Scale (Teen Version) (Weissberg‐Benchell et al., 2011); PARQ‐Global Distress, Parent‐Adolescent Relationship Questionnaire (Global Distress subscale) (Robin et al., 1990); PedsQL‐Diabetes, Paediatric Quality of Life Inventory 3.0 (Diabetes module) (Varni et al., 2003); PedsQL‐Diabetes‐R, Paediatric Quality of Life Inventory 3.2 (Diabetes module revised) (Varni et al., 2018); PedsQL‐Diabetes‐R‐Worry, Paediatric Quality of Life Inventory (Diabetes module revised, Worry sub score) (Varni et al., 2018); PedsQL‐Diabetes‐Worry, Paediatric Quality of Life Inventory (Diabetes module, Worry sub score) (Varni et al.,2003); PedsQL‐Generic, Paediatric Quality of Life Inventory 4.0 (General) (Varni et al. 2001, 2003); PHQ‐9, Patient Health Questionnaire‐9 (Kroenke et al., 2001); PIP, Paediatric Inventory for Parents (Streisand, et al., 2001); PSS, Perceived Stress Scale (Cohen et al., 1983); QoL, Quality of life; SCL‐90‐R‐GSI, Symptom Checklist‐90 (Revised, Global Severity Index sub score) (Derogatis, 1993); TAU, Treatment as usual; WEMWBS, Warwick Edinburg Mental Wellbeing Scale (Tennant et al., 2007); WHO‐5, World Health Organization‐Five Well‐Being Index (World Health Organization, 1998); W/L, Waitlist; WS, Worry Scale (Ireys et al., 1997).

^a^ Timepoint unable to be included in meta‐analysis due to categorizingcategorising studies into immediate post, short term FU (≤ 6 months) and long term FU (> 6 months) and needing three or more studies in each outcome timepoint category to run analysis.

^b^ Data excluded from meta‐analysis to avoid duplication (Ambrosino et al.'s 2008 data already reported in Grey et al. 2011 and Commissoriat et al.'s 2023 data already reported in Laffel et al. 2021).

^c^ Control group excluded from meta‐analysis as a non‐superiority trial approach taken for studies with three arms.

^d^ Active control chosen over TAU for meta‐analysis as TAU not present for all study variables across all study timepoints.

^e^ Insufficient outcome data across studies to include outcome in meta‐analysis.

^f^ Data collected but not reported in a format to allow it to be included in the meta‐analysis.

^g^ Timepoint unable to be included in meta‐analysis due to insufficient data reporting.

^h^ Studies (*k* = 9) unable to be included in meta‐analysis due to insufficient data reporting.

^i^ Timepoint unable to be included in meta‐analysis due to absence of control group.

Caregiver mean ages ranged from 33.6 to 47.8 years. However, most caregivers were predominantly or exclusively female (*k* = 30) or their gender was not stated (*k* = 27). Only one study was exclusively for male caregivers [25]. However, most child samples were evenly mixed across genders. The mean age of children ranged from 4.1 to 15.3 years, with a mean duration since Type 1 Diabetes onset spanning 0.2–9.2 years.

Most interventions were delivered to caregivers and children together (*k* = 37) or solely to caregivers (*k* = 19). A minority were delivered to caregivers and children separately (*n* = 5). There was a large range of intervention durations (1 day‐12 months). Around two thirds of interventions were delivered face to face (*k* = 43) and a third online (*k* = 21). Most interventions were delivered individually (*k* = 42) rather than in groups (*k* = 14) or self‐led (*k* = 5).

The intervention content was diverse, including interventions drawing on or including psychoeducation and coping skills (*k* = 36), family system theory or family therapy models (*k* = 11), social cognitive therapy or social learning theory (*k* = 11), social support (*k* = 9), behavior change (*k* = 9), CBT (*k* = 8), motivational interviewing (*k* = 6), acceptance and commitment therapy (*k* = 1), solution focused therapy (*k* = 1), strengths based intervention (*k* = 1) and positive psychology (*k* = 1). Twenty‐six studies covered communication or parent–child teamwork in their intervention. Detailed information about sample and study characteristics can be found in Table 1. Due to interventions falling into multiple categories, the study numbers frequently sum to over 58.

### 3.3 Risk of Bias

**FIGURE 2:**
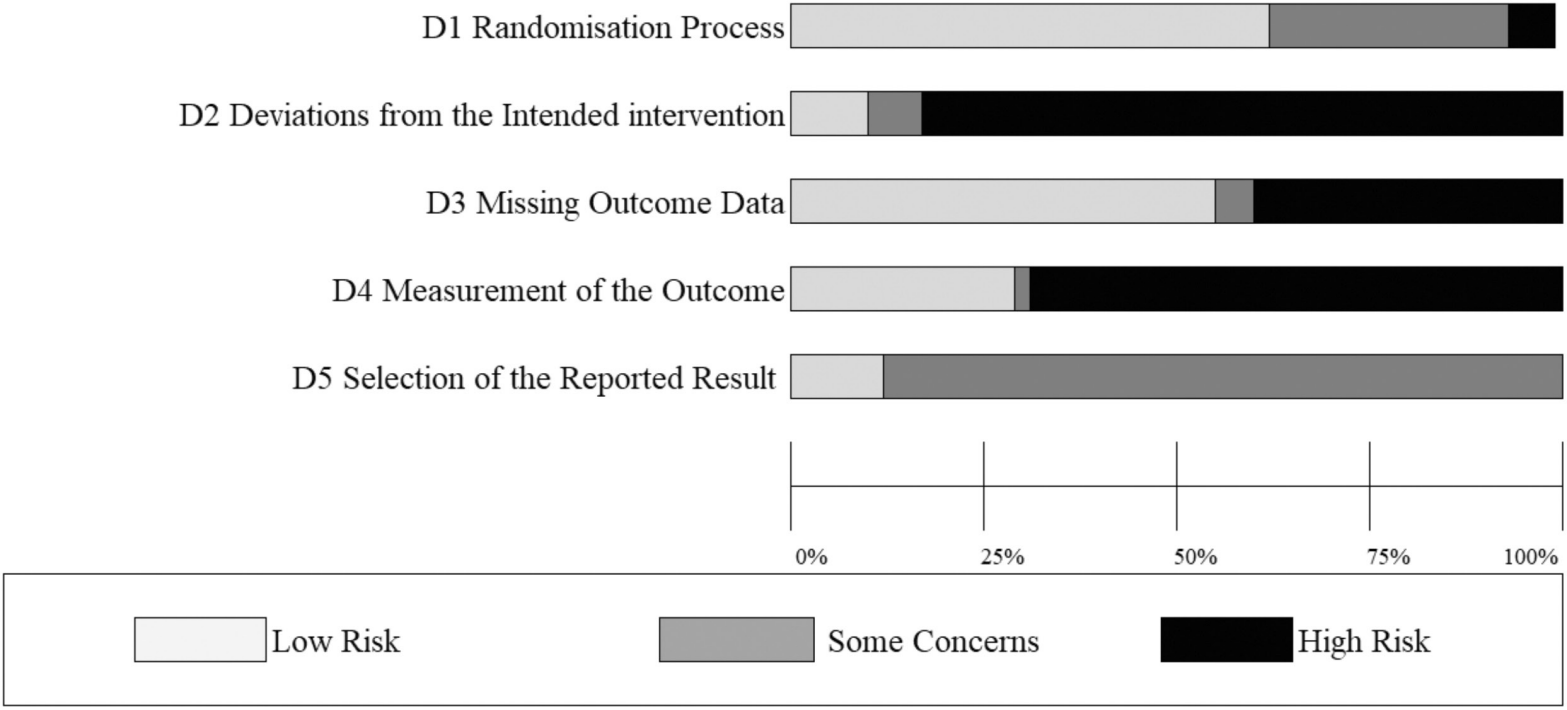
Summary of bias domains by percentage using Risk of Bias 2 Tool for systematic review.

Overall, the methods of the 58 studies in the review were of poor quality, with 94.8% being classified as high risk of bias overall. Studies used self‐report outcome measures for all outcomes other than blood glucose and were unable to double‐blind participants due to the nature of undertaking a psychological intervention. This led to ratings of high bias in deviations from the intended intervention and measurement of outcome by the RoB2, which informed the overall rating [26]. Bias due to missing outcome data was common due to studies not conducting sensitivity analysis and/or not using intention‐to‐treat analyses. Most studies were also rated as having “some concerns” with selective reporting. This was because authors did not explicitly state that the statistical analysis plan was confirmed before unblinded data was released. Although most studies (62%) provided adequate information to evidence true randomization, it is noted that 38% of studies still presented some concerns or high risk of bias. The details of risk of bias assessment for each study are presented in Figures 2 and S2.

### 3.4 Meta‐Analysis Findings

See Table 2 for full statistical findings of the 18 meta‐analyses conducted. There was sufficient data to analyze all outcomes immediately post‐intervention, two thirds at short‐term follow‐up, and one third at long‐term follow‐up.

**TABLE 2:** Forest plots for caregiver and child outcomes across post‐intervention, short‐term follow up, and long‐term follow‐up timepoints.

Outcome	Immediately post‐intervention	Short term follow‐up (≤ 6 months)	Long term follow‐up (> 6 months)
Caregiver psycho‐logical distress	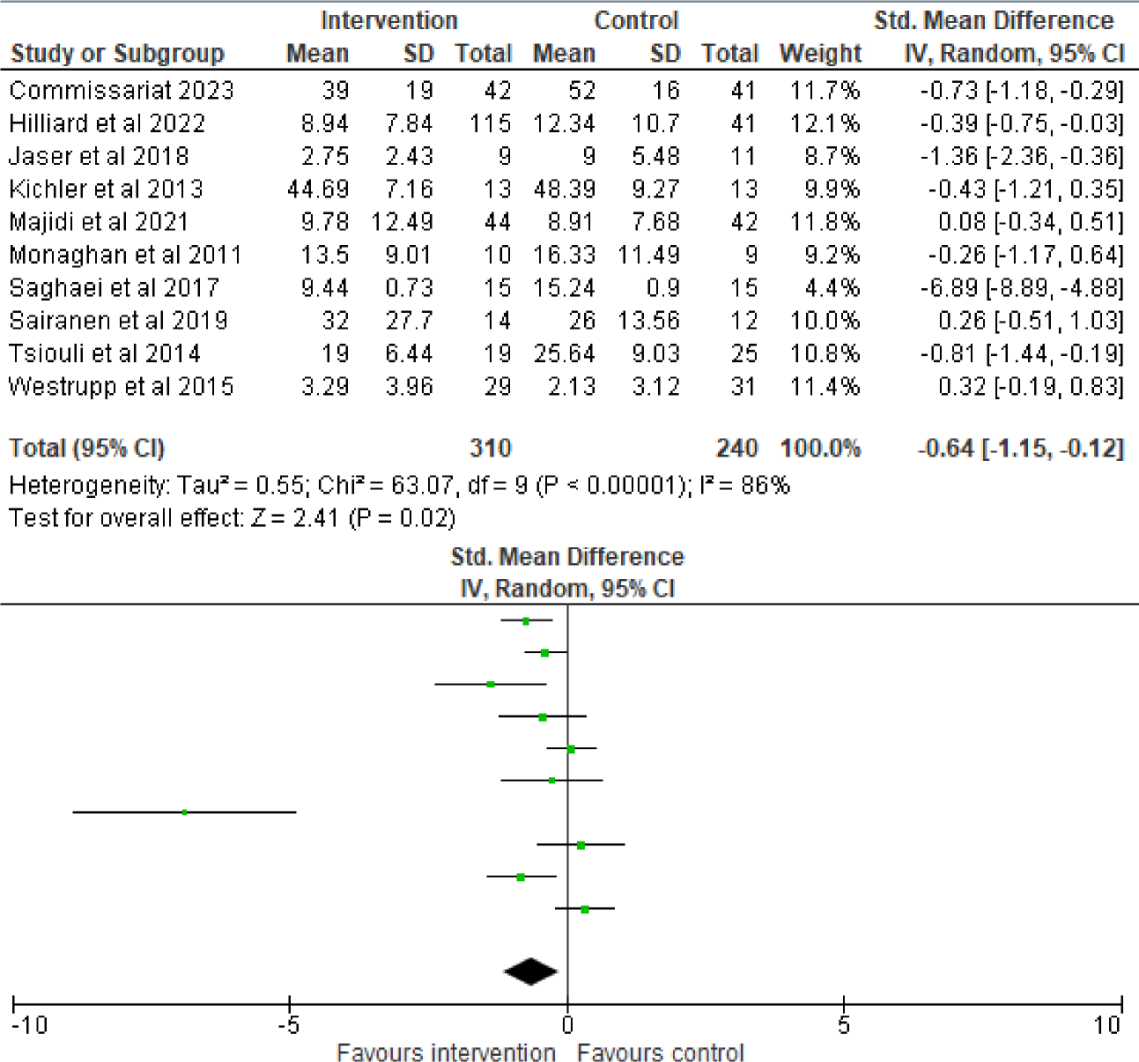	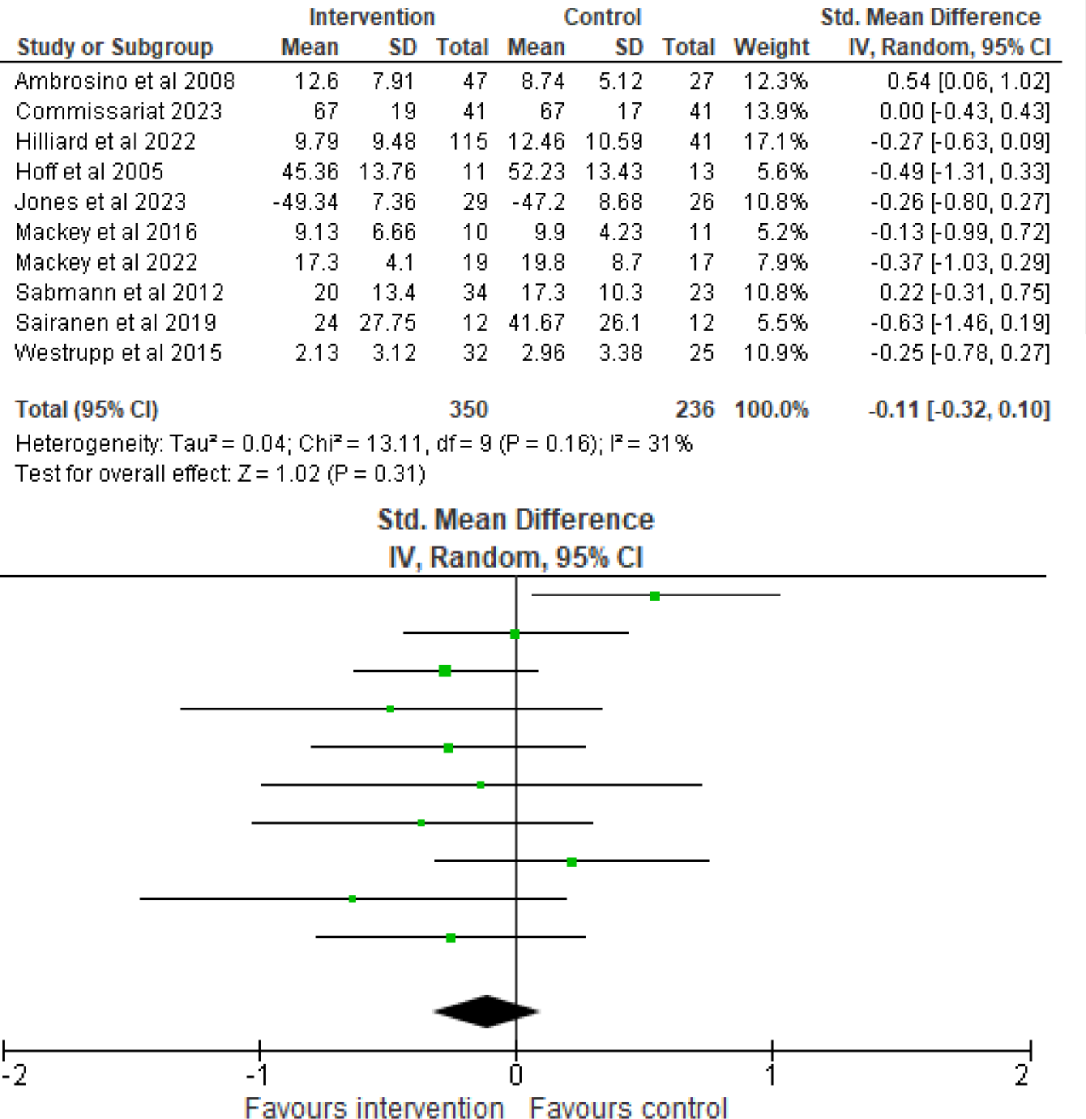	< 3 studies so meta‐analysis unable to be conducted.
Child psycho‐logical distress	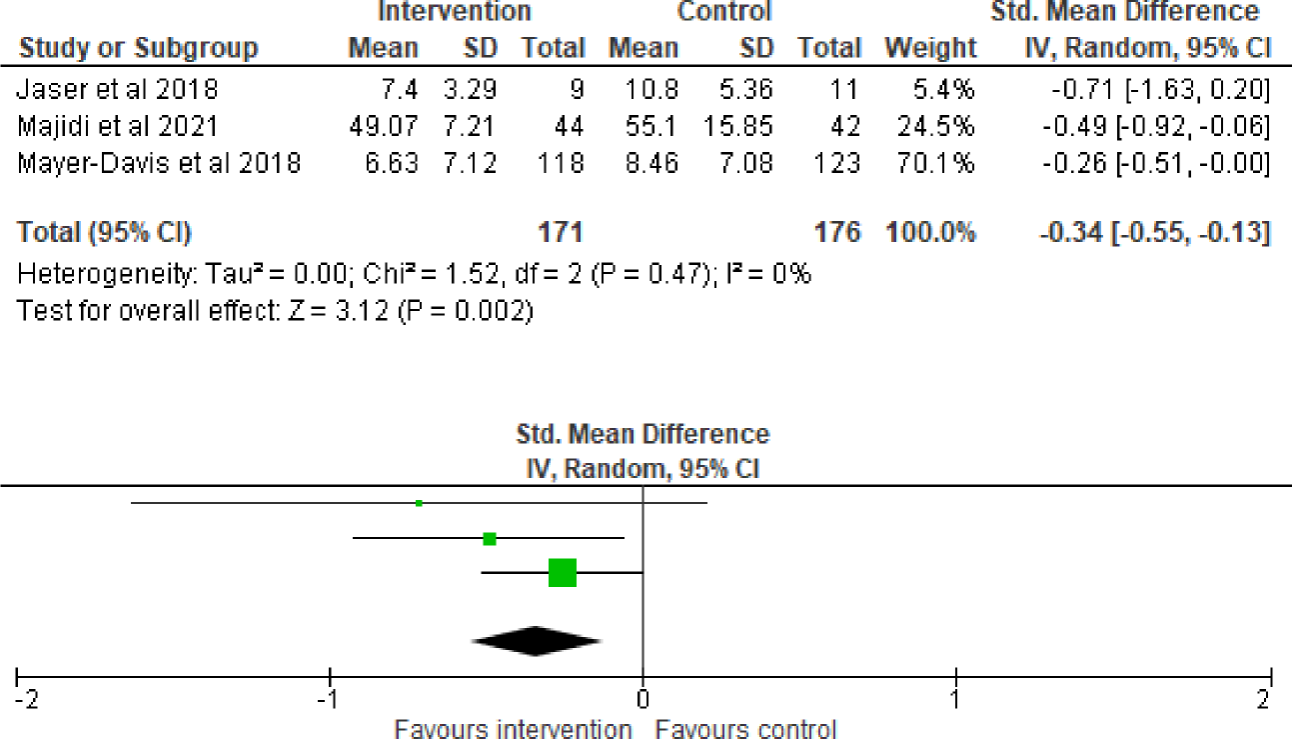	< 3 studies so meta‐analysis unable to be conducted.	< 3 studies so meta‐analysis unable to be conducted.
Caregiver diabetes distress	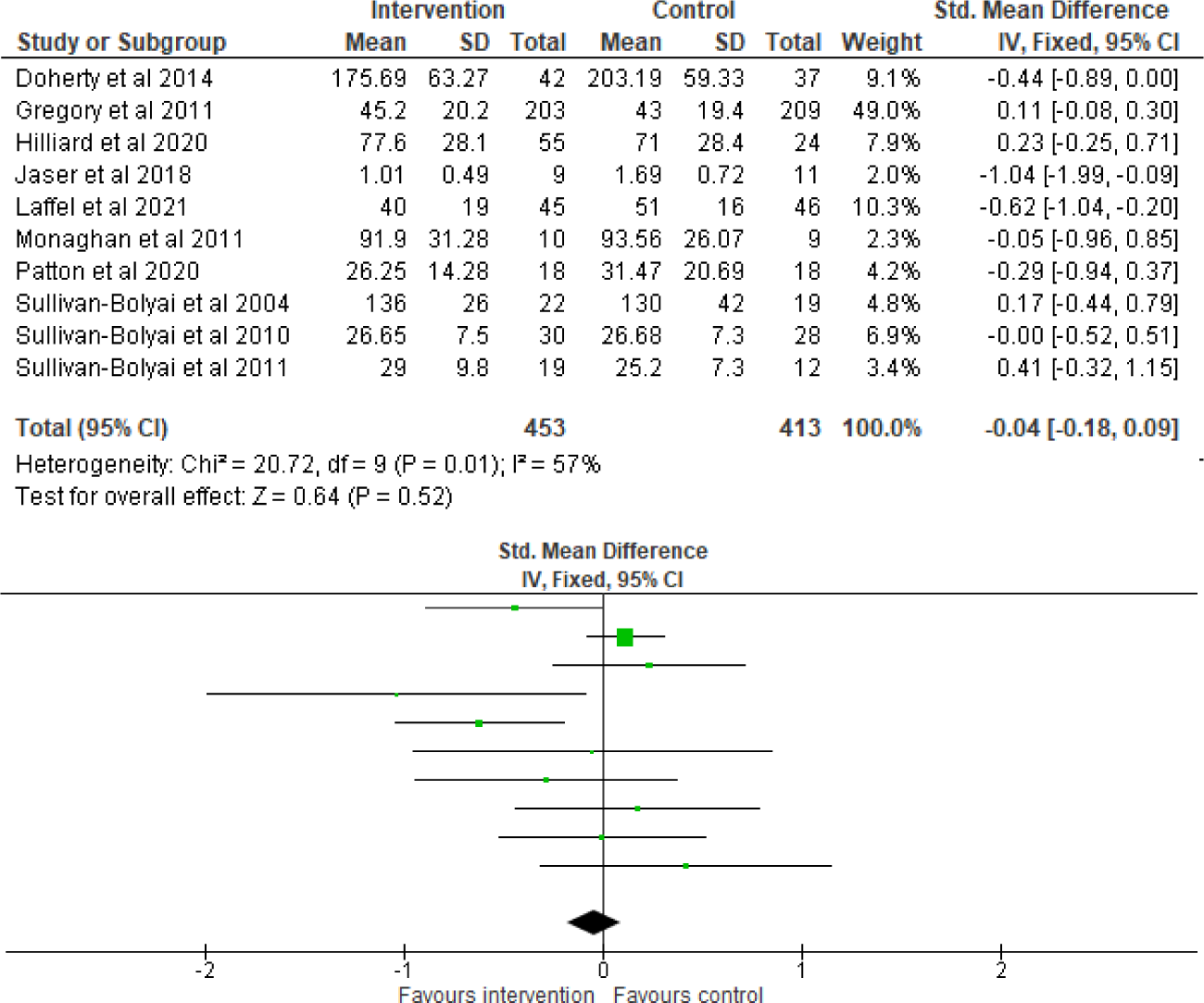	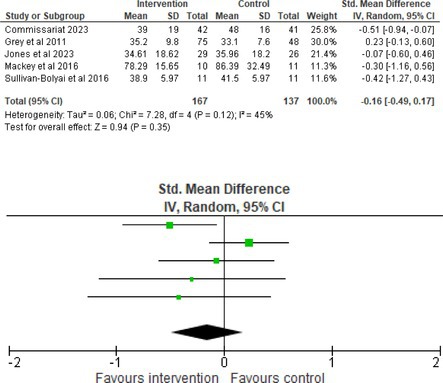	< 3 studies so meta‐analysis unable to be conducted.
Child diabetes distress	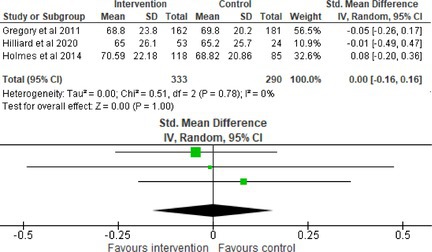	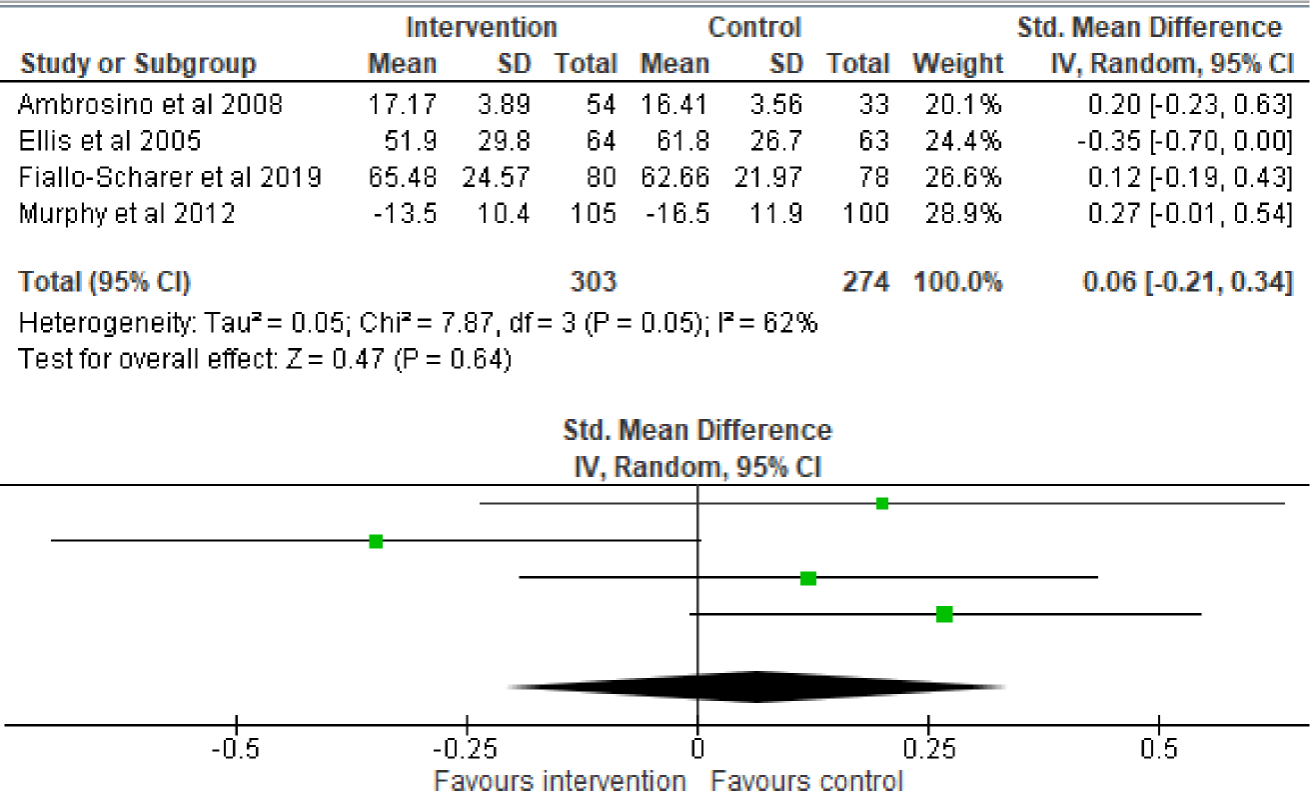	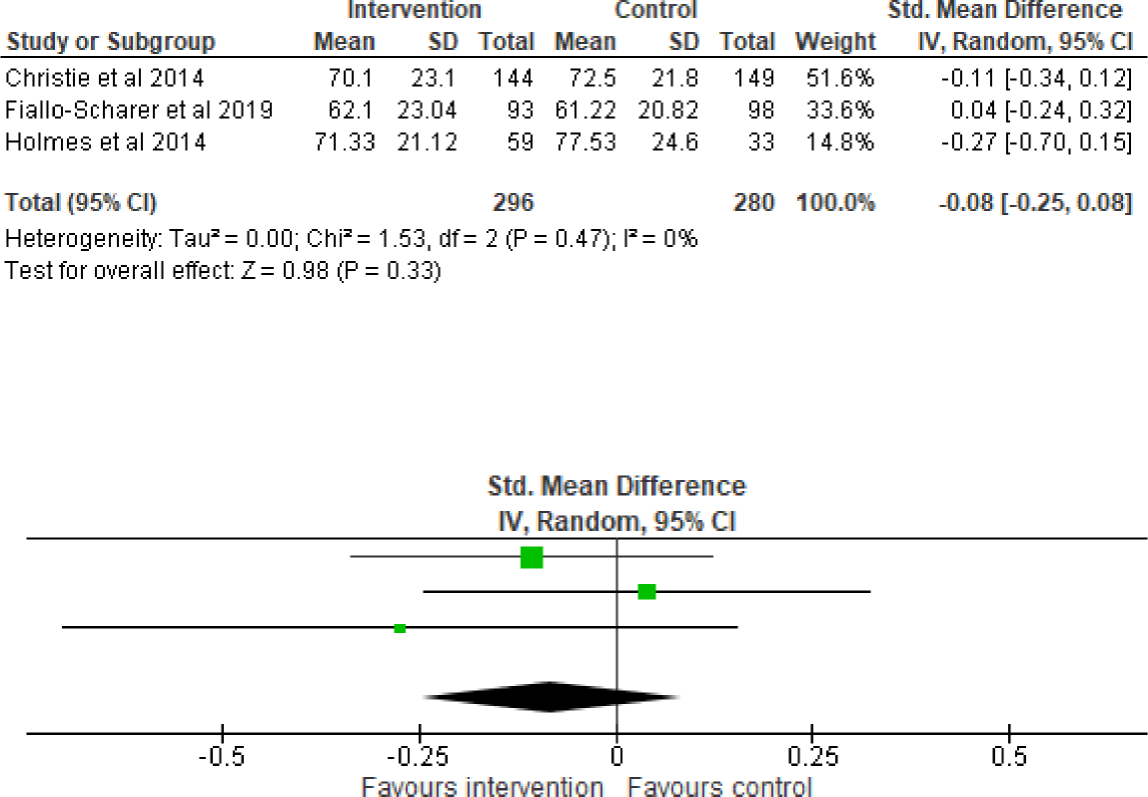
Caregiver diabetes family conflict	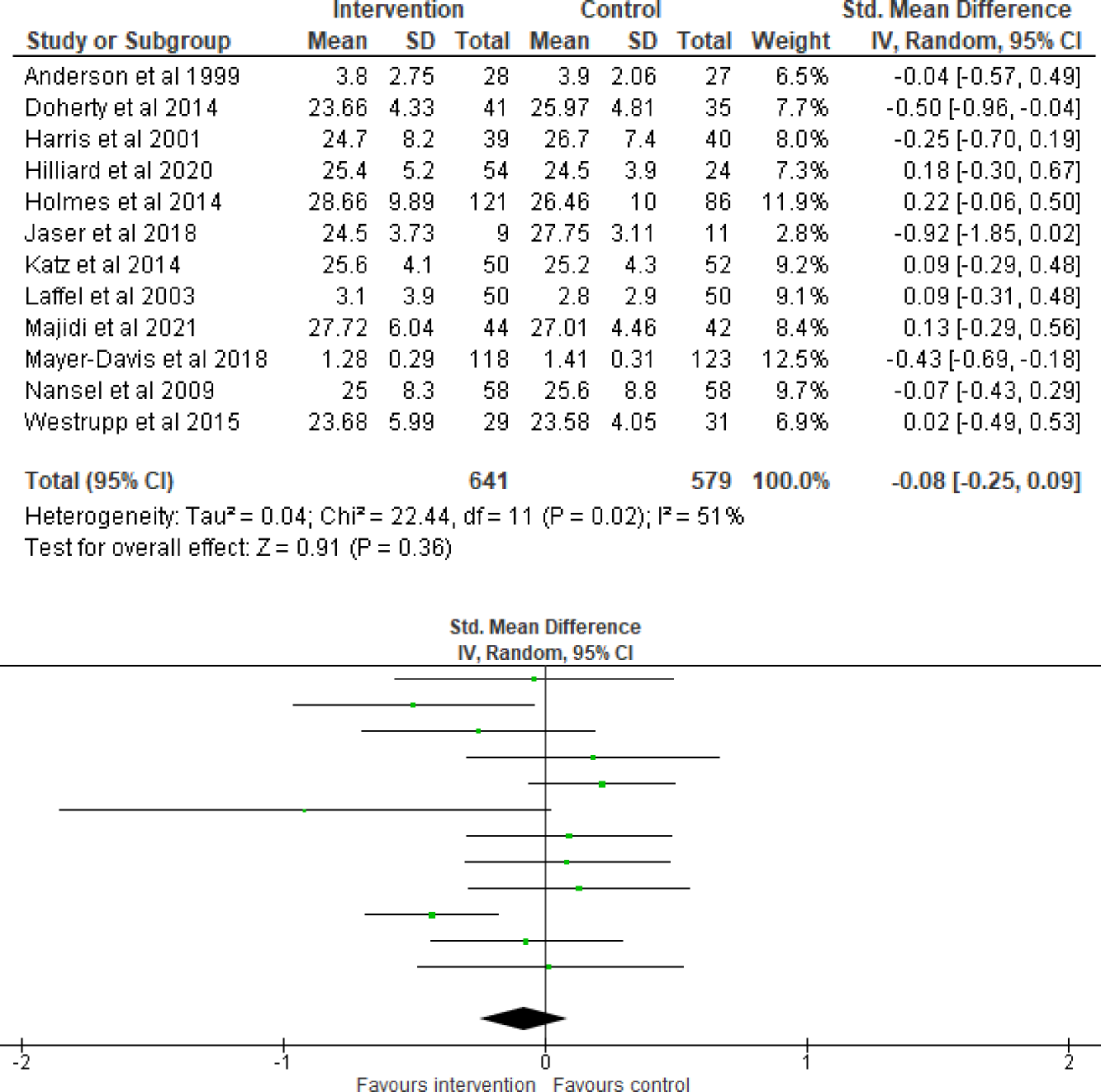	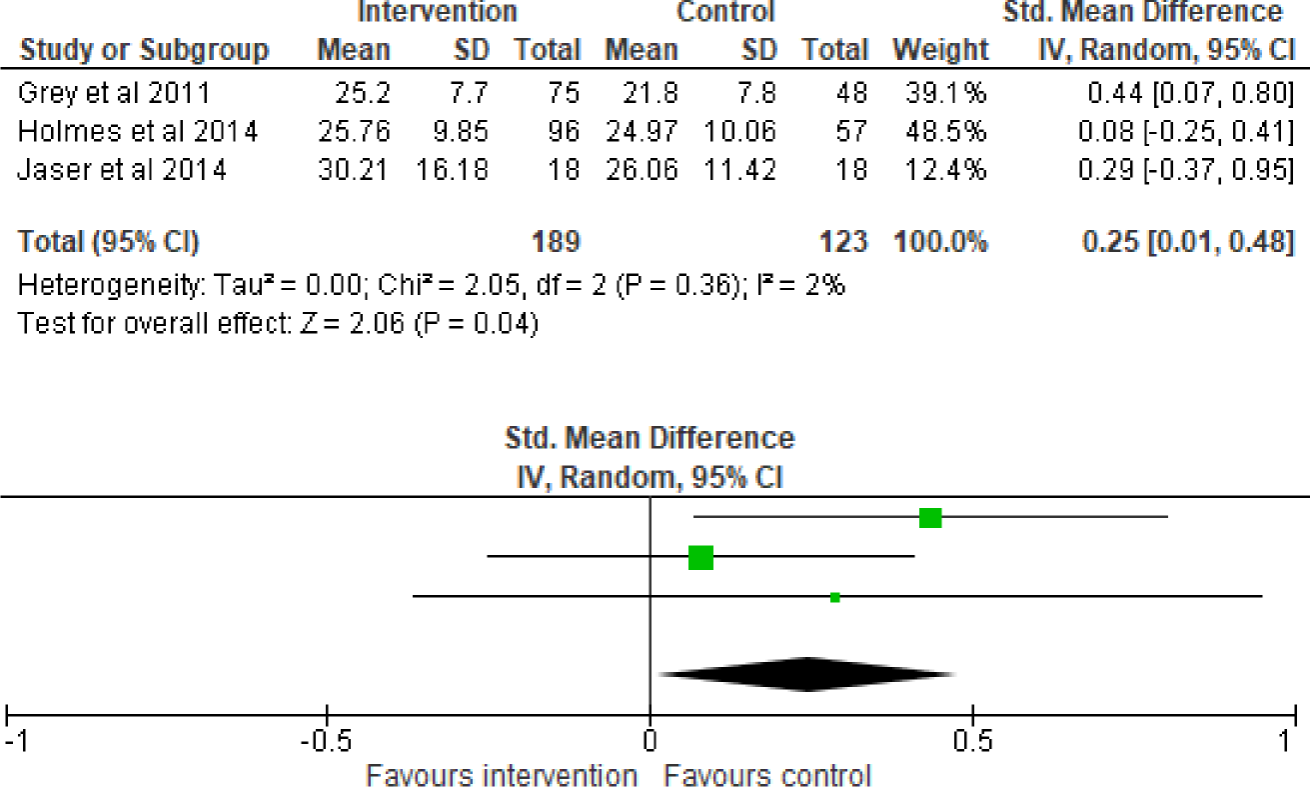	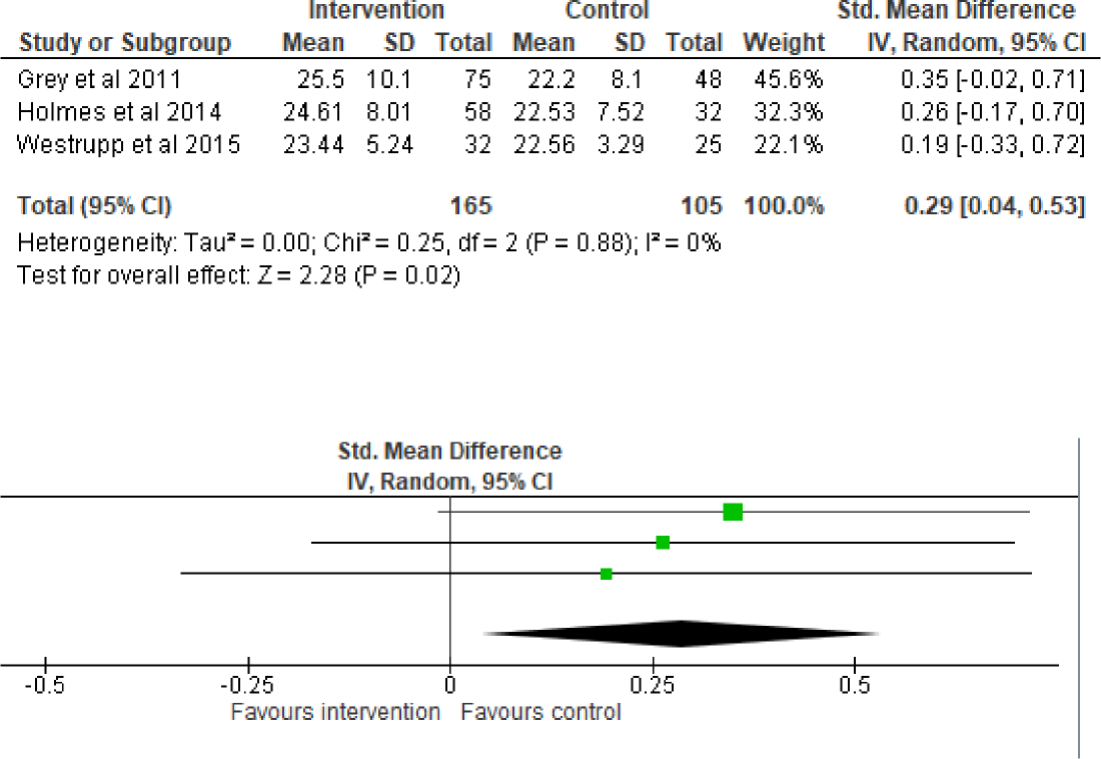
Child diabetes family conflict	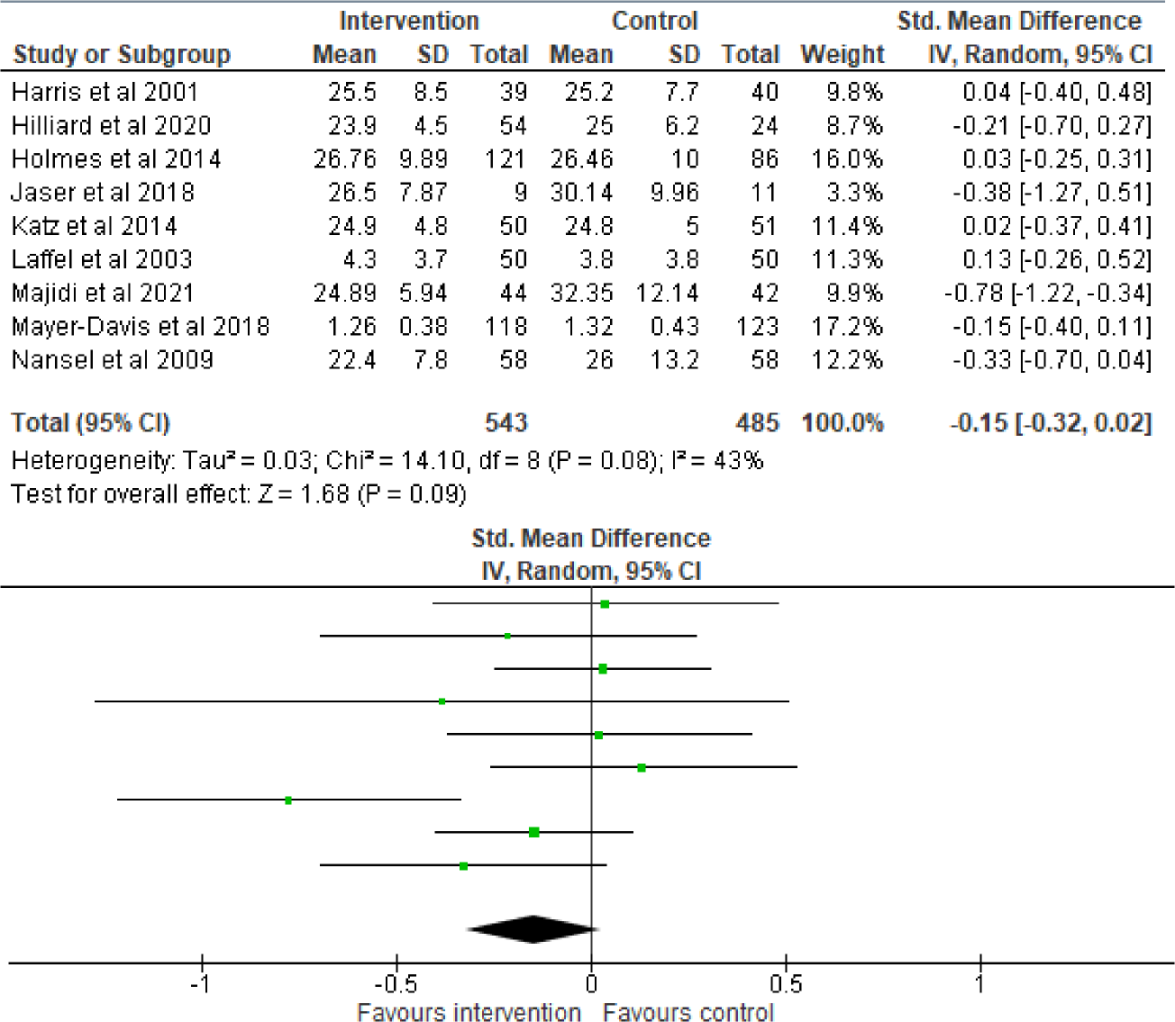	< 3 studies so meta‐analysis unable to be conducted.	< 3 studies so meta‐analysis unable to be conducted.
Child quality of life	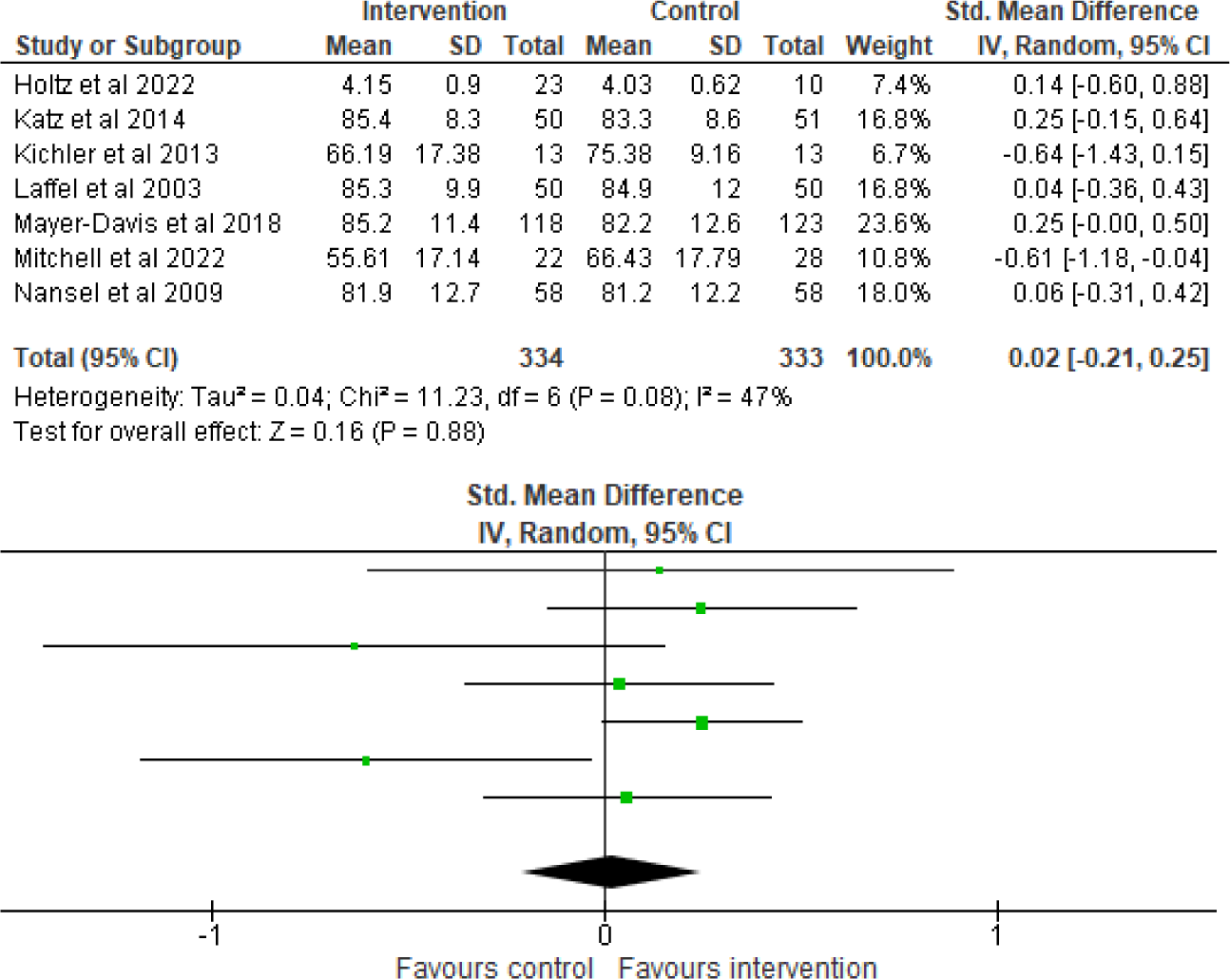	< 3 studies so meta‐analysis unable to be conducted.	< 3 studies so meta‐analysis unable to be conducted.
Child diabetes quality of life	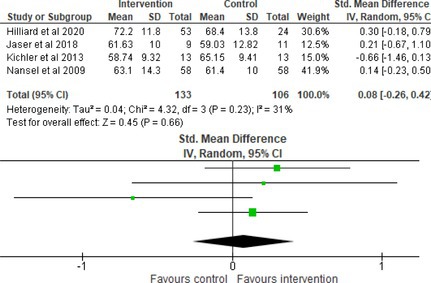	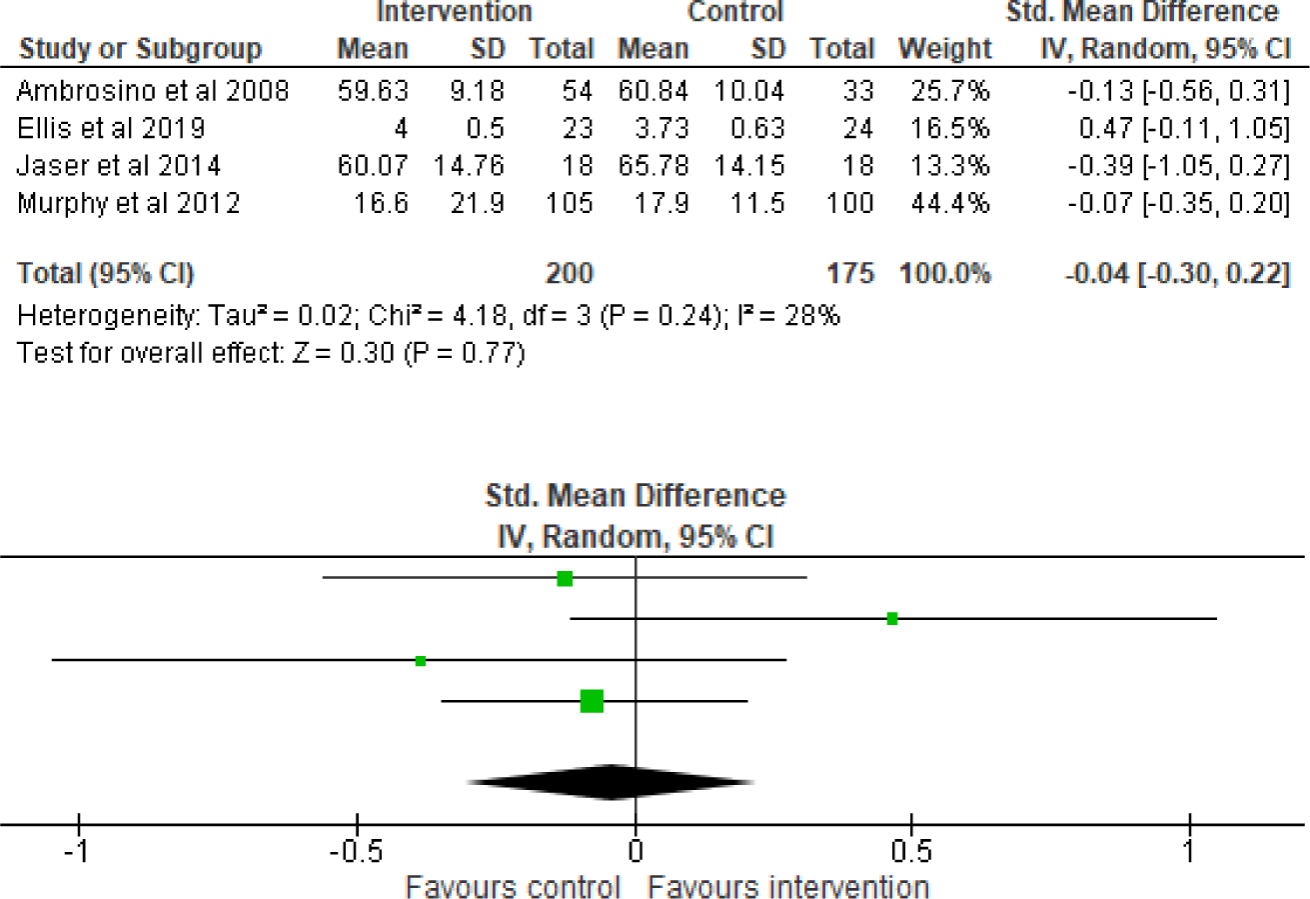	< 3 studies so meta‐analysis unable to be conducted.
Child blood glucose	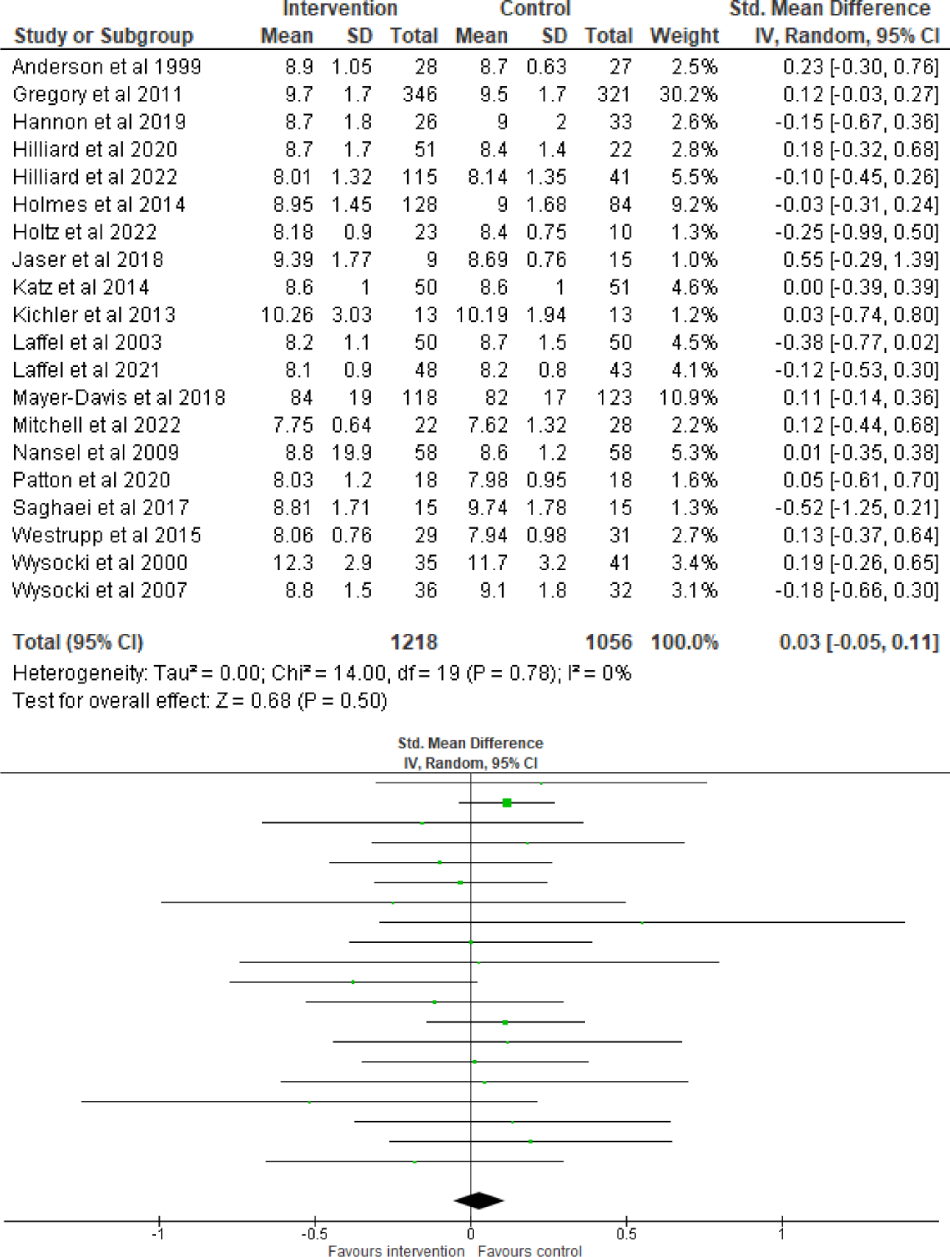	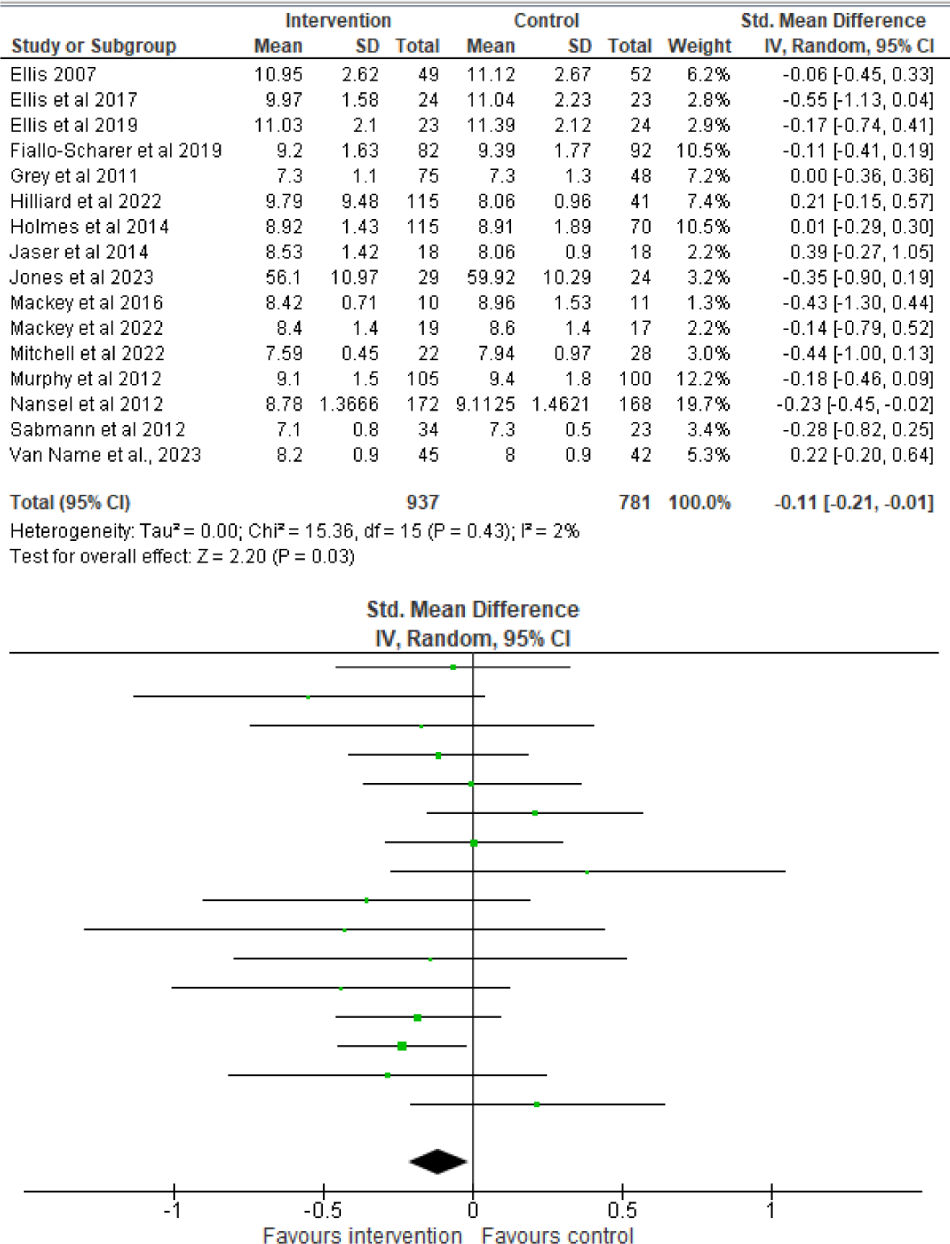	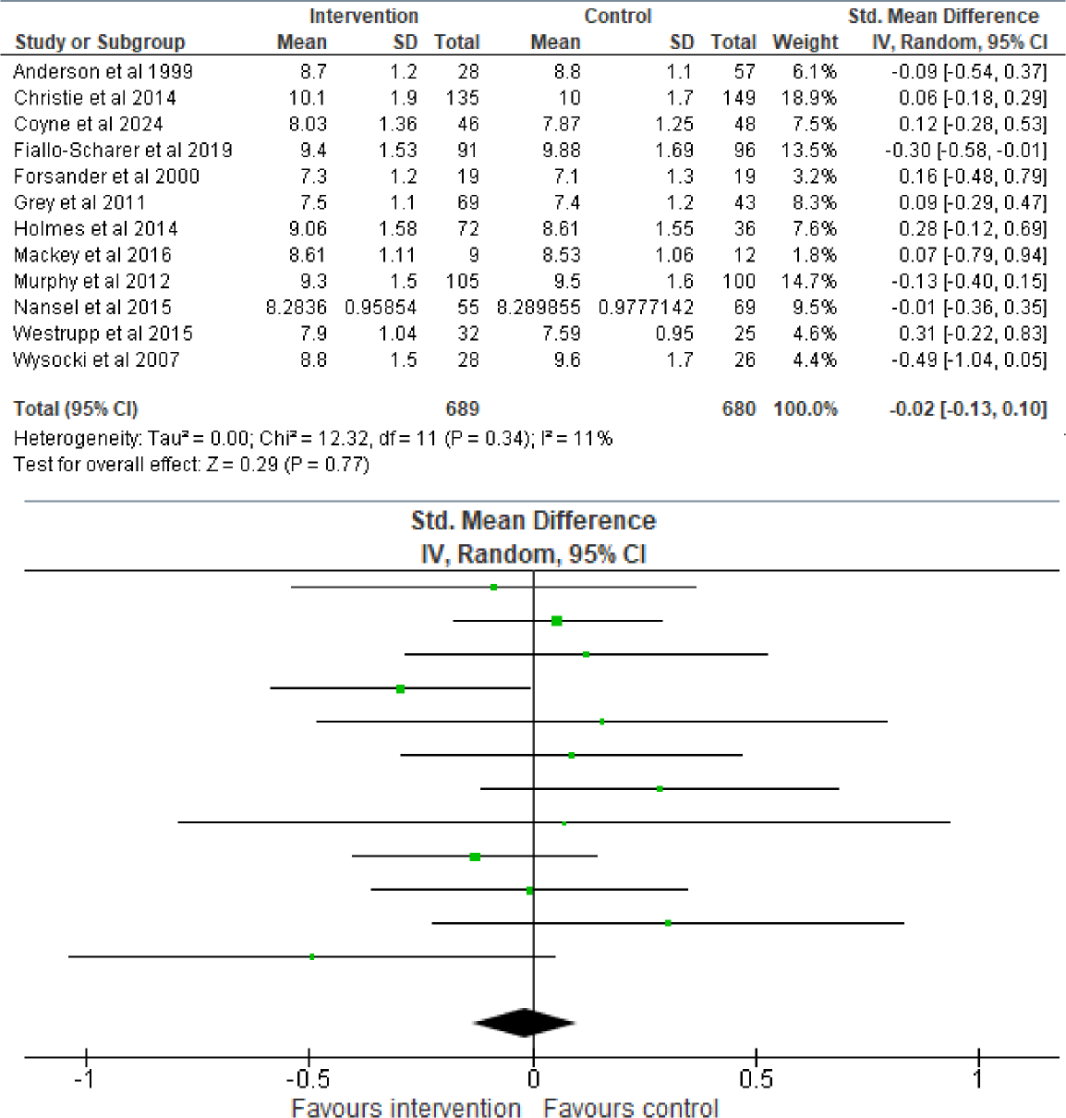

### 3.5 Psychological Distress

Data from 10 studies (*n* = 550) showed that psychological interventions significantly reduced caregiver psychological distress relative to controls post‐intervention with a medium effect size (SMD = −0.64, 95% CI = −1.15, −0.12, *p* = 0.02) and high heterogeneity (*I* ^2^ = 86%, *p* < 0.0001). However, data from 10 studies (*n* = 586) showed no significant effect of psychological interventions on caregiver psychological distress relative to controls at short‐term follow‐up.

Data from three studies (*n* = 347) showed that psychological interventions significantly reduced child psychological distress relative to controls post‐intervention (SMD = −0.34, 95% CI = −0.55, −0.31, *p* = 0.002) with a small effect size and low heterogeneity (*I* ^2^ = 0%, *p* = 0.47).

### 3.6 Diabetes Distress

Data from 10 studies (*n* = 866) and five studies (*n* = 304) respectively showed no significant effect of psychological interventions on caregiver diabetes distress relative to a control post‐intervention, nor at short‐term follow‐up.

Data from three (*n* = 623), four (*n* = 577) and three studies (*n* = 576) respectively showed no significant effect of psychological interventions on child diabetes distress relative to a control post‐intervention, at short‐term follow‐up, nor at long‐term follow‐up.

### 3.7 Diabetes Family Conflict

Data from 12 studies (*n* = 1220) showed no significant effect of psychological interventions on caregiver diabetes family conflict relative to a control post‐intervention. However, data from three studies (*n* = 312 and *n* = 270, respectively) showed that controls significantly reduced caregiver diabetes family conflict relative to psychological interventions at short‐term follow‐up (SMD = 0.25, 95% CI = 0.01, 0.48, *p* = 0.04) and at long‐term follow‐up (SMD = 0.29, 95% CI = 0.04, 0.53, *p* = 0.02) with small effect sizes and low heterogeneity (*I* ^2^ = 2%, *p* = 0.36 and *I* ^2^ = 0%, *p* = 0.88, respectively).

Data from nine studies (*n* = 1028) showed no significant effect of psychological interventions on child diabetes family conflict relative to a control post‐intervention.

### 3.8 Child Quality of Life

Data for seven studies (*n* = 667) showed no significant effect of psychological interventions on child quality of life relative to a control post‐intervention.

### 3.9 Child Diabetes Quality of Life

Data for four studies (*n* = 239) and four studies (*n* = 375) respectively showed no significant effect of psychological interventions on child diabetes quality of life relative to a control post‐intervention nor at short‐term follow‐up.

### 3.10 Child Blood Glucose

Hemoglobin A1C (HbA1c) was used as the blood glucose indicator across all studies. Data for 20 studies (*n* = 2274) and 12 studies (*n* = 1369) respectively showed no significant effect of a psychological intervention on child blood glucose relative to a control post‐intervention nor at long‐term follow‐up. However, data from 16 studies (*n* = 1718) showed that psychological interventions significantly improved child blood glucose relative to controls at short‐term follow‐up with a small effect size (SMD = −0.11, 95% CI = −0.21, −0.01, *p* = 0.03) and low heterogeneity (*I* ^2^ = 2%, *p* = 0.43).

### 3.11 Publication Bias

Funnel plots were examined for the seven meta‐analyses which had 10 or more studies [26] and indicated relatively low asymmetry (Figure S3). However, there was a trend for studies to cluster towards the top of the funnel, indicating a possible publication bias towards larger sample studies. This was particularly noticeable for caregiver psychological and diabetes distress post‐intervention, as well as caregiver diabetes family conflict post‐intervention.

## 4 Discussion

The aim was to conduct a systematic review and meta‐analysis to estimate the effect of psychological interventions for caregivers and families of children with Type 1 Diabetes on caregiver and child functioning. The systematic search identified 58 RCTs, and meta‐analyses were conducted on the 51 studies that had sufficient data to be included.

Meta‐analysis demonstrated that psychological interventions produce a small–medium reduction in child and caregiver psychological distress post‐intervention in comparison to control groups. This is consistent with previous research which found parenting interventions significantly reduce parents' depression and distress and help them ask for positive social support [21]. It appears logical that an intervention reducing caregiver psychological distress would also reduce child psychological distress, as child mental health difficulties are more likely when a parent is distressed [27]. However, the significant effect for caregiver psychological distress was not maintained at short‐term follow‐up, similar to previous reviews [20].

Meta‐analysis found psychological interventions also produced a significant, small improvement in child blood glucose at short‐term follow‐up in comparison to control groups, consistent with previous systematic reviews and meta‐analysis [18, 22]. However, this effect was not observed post intervention nor long‐term follow‐up. Previous research suggests that blood glucose outcomes may be sensitive to the intervention type delivered, with coping skills training and some forms of CBT showing promise [28, 29]. However, it was not possible to look at intervention type as a moderator due to high heterogeneity and low numbers of studies utilizing each type. Interventions frequently integrated multiple theories and techniques, meaning they could not be clearly categorized. Future trials should more clearly detail their intervention components to allow future reviews to conduct sub‐group analysis by intervention type.

Contrary to expectations, participants allocated to control conditions showed significantly greater improvements in caregiver diabetes family conflict at short and long‐term follow‐up than those assigned to psychological interventions, with small effect sizes. However, these two meta‐analyses only sampled a small number of studies (k = 3), and so their significance is influenced by the presence of Grey et al.'s [30] RCT. They reported parent diabetes family conflict at baseline to be significantly higher in the intervention group. They controlled for this difference whilst looking at significance across time, concluding no significant changes in diabetes‐related conflict from baseline to follow‐up. However, this is not reflected in the meta‐analyses where Grey et al.'s [30] end point scores favor the control, as only significance at a specific timepoint is captured in a meta‐analysis. Therefore, the significant parent diabetes family conflict findings are likely skewed by significant baseline differences in a single study [30]. More robust randomization in future RCTs is recommended to prevent significant baseline differences occurring. To establish the robustness of the findings, the meta‐analyses on parent diabetes family conflict should be replicated when there is more research in this area.

### 4.1 Strengths and Limitations

This is the largest review to date of psychological interventions for caregivers and families of children with Type 1 Diabetes, with previous systematic reviews ranging from 7 to 25 studies [19, 20] and meta‐analyses of 17 studies [21]. Therefore, this review provides an up‐to‐date synthesis of the research. Unlike previous reviews [21], both child and caregiver functioning outcomes were included, providing the field with a more holistic assessment of family functioning. A strength of the broad inclusion criterion is that it allowed a comprehensive overview of the literature. However, this simultaneously introduced significant variability in intervention content and delivery, control groups, samples, and outcome measures, reflecting the complexity of real‐world clinical practices. This makes identifying mechanisms of change more complex. However, exploring these variations can help inform the design of more standardized approaches. Therefore, intervention type, duration, and delivery should be considered in future sub‐group analyses if there are sufficient trials available. Additionally, a more consistent approach to measuring caregiver psychological distress and child and caregiver diabetes‐specific distress would increase comparability of outcomes across studies for future review, as these were noticeably varied. Continuing forward, future RCTs are recommended to keep using the Diabetes Family Conflict Scale [31] and Pediatric Quality of Life Inventory 4.0 (General) [32, 33] to measure family conflict and quality of life, as these have been used fairly consistently.

A strength of this review was that it assessed the maintenance of studies, unlike previous reviews. Nonetheless, due to such variability in follow‐up periods, for analytical purposes data points were categorized into post‐intervention, short‐term follow‐up, and long‐term follow‐up groupings. There were sufficient studies to conduct a meta‐analysis on all nine outcomes immediately post‐intervention, but only six at short‐term follow‐up and three at long‐term follow‐up. This illustrates the need for longer assessment to determine maintenance of interventions, which may allow adequately powered sub‐group analysis in future meta‐analyses.

The review is limited by the majority of studies demonstrating high concern for risk of bias, particularly in the domains of deviations from intended interventions and measurement of outcomes. This article was conducted within recognized practice. However, it is acknowledged that with variable study quality and small study numbers for many outcome categories, results should be interpreted with caution. Publication bias is also a common limitation of meta‐analyses. Funnel plots of the seven analyses with sufficient study numbers indicated findings to be robust. Therefore, publication bias was unlikely to have heavily influenced the findings for caregiver diabetes psychological distress and family conflict post‐intervention and child blood glucose. However, the remaining 11 analyses were unable to be assessed, a limitation of small study numbers in a meta‐analysis.

Furthermore, the majority of studies sampled mothers. This is a concern because growing research emphasizes the role of fathers in learning and adjusting to their child's chronic condition [34]. However, mothers and fathers frequently differ in parenting styles and practices [35]. Therefore, future research should include male caregivers, exploring how they might respond differently to interventions than their female counterparts. Greater involvement of fathers in diabetes management is associated with improved psychological wellbeing in mothers and improved family functioning [36]. Therefore, intervention effect may be increased by involving fathers. In addition, the majority of studies sampled western cultures, predominately America. This is a limitation because parent interventions need to be adapted to respect cultural values, traditions, and needs of different ethnic groups [37]. For example, in collectivist cultures greater value is placed on extended family and interdependence [38]. Therefore, working with the whole family system to identify a family's personal goals, rather than imposing professional views around independent Type 1 Diabetes management, may be important in collectivist cultures. In order to improve the generalizability of the findings to impact a wider population, underrepresented demographics should be taken into consideration in future research.

### 4.2 Implications for Practice and Research

The review found support for the beneficial role of psychological interventions in reducing caregiver and child psychological distress and improving blood glucose. Therefore, evidence suggests that it would be beneficial for psychological interventions to be more readily available to families with a child with Type 1 Diabetes. Although difficulties with high heterogeneity limit conclusions about intervention type and delivery, Table 2 illustrates particularly large effects for caregiver psychological distress reduction post‐intervention using an eight‐week individual CBT intervention for caregivers relative to TAU [39]. Similarly, large effects were also observed for child diabetes family conflict using a 12‐month caregiver and child group intervention relative to TAU [40]. However, in the current economic climate, interventions must also undergo health economic analysis to ensure they are practical for service delivery. Therefore, further RCTs should prioritize investigating the content of these two interventions using larger samples. This may involve condensing the intervention material to compare different lengths to assess cost effectiveness.

To rectify the methodological quality of the field, future RCTs need to select validated and previously used outcome measures to aid meta‐analyses, ensure adequate power, and publish study protocols in advance to reduce the risk of bias when reporting outcomes. Publication of protocols will also allow more in‐depth intervention detail to be shared, supporting future understandings of change mechanisms. More longitudinal follow‐ups and consideration of culturally and gender diverse samples have also been highlighted.

### 4.3 Conclusion

Overall, this is the largest scale meta‐analyses reviewing the effects of psychological interventions for caregivers and families in the Type 1 Diabetes field to date. Findings suggest promising outcomes, but highlight the importance of robust randomization in RCTs to prevent significant baseline differences. The two interventions showing distinctly large effect sizes (39; 40) should be investigated in further RCTs with consideration given to dose response to move the field forward more strategically, whilst balancing cost‐efficiency. In conclusion, the meta‐analysis provides evidence that psychological interventions may successfully reduce caregiver and child psychological distress and improve blood glucose.

## Author Contributions

All authors contributed significantly and in keeping with the latest guidelines of the International Committee of Medical Journal Editors and are in agreement with the content of the manuscript. Jones, Harrington, John and Satherley conceived the project. Read, O'Donnell and Wakelin completed the literature searches. Wakelin and Williams completed the data extraction and Francois‐Walcott checked the accuracy. Wakelin led on the analysis with Jones' support. Wakelin and Francois‐Walcott created the tables and figures and Francois‐Walcott completed the references. Wakelin led on writing the manuscript, with support from Harrington and Read for the introduction. All authors edited the manuscript.

## Ethics Statement

Ethics approval was not required because the article was a review paper. No patient consent was required because no participants were recruited and so no new patient data was collected. The article summarizes previously published data.

## Conflicts of Interest

The authors declare no conflicts of interest.

## Supporting information


**Appendix S1.** Supporting Information.

## Data Availability

All available data is publicly available within the article.
