# Supplementary material for: The Effectiveness of Psychological Interventions for Families of Children With Type 1 Diabetes on Caregiver and Child Functioning: A Systematic Review and Meta‐Analysis
Source: J Diabetes. 2025 Jun 17;17(6):e70112. doi: 10.1111/1753-0407.70112 (PMC12171233; doi:10.1111/1753-0407.70112)
Supplement: Supplementary file 1 — Appendix S1. Supporting Information. [file JDB-17-e70112-s001.docx]

**Supplementary Tables and Figure Contents**

**Tables**

Supplementary Table S1. PRISMA Statement

Supplementary Table S2. Outcome measures of the data extracted in the meta-analyses

Supplementary Table S3. References for the papers included in the systematic review in alphabetical order

**Figures**

Supplementary Figure S1. Search Terms

Supplementary Figure S2. Full summary of bias domains using Risk of Bias 2 Tool

Supplementary- Figure S3. Funnel plots for meta-analyses with more than ten studies.

**Supplementary Tables**

Supplementary Table S1. PRISMA Statement

| **Section and Topic** | **Item #** | **Checklist item** | **Location where item is reported** |
| --- | --- | --- | --- |
| **TITLE** | | |  |
| Title | 1 | Identify the report as a systematic review. | Yes- Title |
| **ABSTRACT** | | |  |
| Abstract | 2 | See the PRISMA 2020 for Abstracts checklist. | Yes- Abstract |
| **INTRODUCTION** | | |  |
| Rationale | 3 | Describe the rationale for the review in the context of existing knowledge. | Yes- Intro |
| Objectives | 4 | Provide an explicit statement of the objective(s) or question(s) the review addresses. | Yes- Intro |
| **METHODS** | | |  |
| Eligibility criteria | 5 | Specify the inclusion and exclusion criteria for the review and how studies were grouped for the syntheses. | Yes- Methods: Eligibility criteria & Data analysis.  Note- Studies were grouped for meta-analysis by outcome and follow-up duration. |
| Information sources | 6 | Specify all databases, registers, websites, organisations, reference lists and other sources searched or consulted to identify studies. Specify the date when each source was last searched or consulted. | Yes- Methods: Search strategy |
| Search strategy | 7 | Present the full search strategies for all databases, registers and websites, including any filters and limits used. | Yes- Methods: Search strategy & Appendix A |
| Selection process | 8 | Specify the methods used to decide whether a study met the inclusion criteria of the review, including how many reviewers screened each record and each report retrieved, whether they worked independently, and if applicable, details of automation tools used in the process. | Yes- Methods: Search strategy & Data extraction |
| Data collection process | 9 | Specify the methods used to collect data from reports, including how many reviewers collected data from each report, whether they worked independently, any processes for obtaining or confirming data from study investigators, and if applicable, details of automation tools used in the process. | Yes- Methods: Search strategy & Data extraction |
| Data items | 10a | List and define all outcomes for which data were sought. Specify whether all results that were compatible with each outcome domain in each study were sought (e.g. for all measures, time points, analyses), and if not, the methods used to decide which results to collect. | Yes- Methods: Eligibility criteria, Search strategy & Data extraction |
|  | 10b | List and define all other variables for which data were sought (e.g. participant and intervention characteristics, funding sources). Describe any assumptions made about any missing or unclear information. | Yes- Methods: Data extraction, Quality assessment of studies, Data analysis |
| Study risk of bias assessment | 11 | Specify the methods used to assess risk of bias in the included studies, including details of the tool(s) used, how many reviewers assessed each study and whether they worked independently, and if applicable, details of automation tools used in the process. | Yes- Methods: Quality assessment of studies |
| Effect measures | 12 | Specify for each outcome the effect measure(s) (e.g. risk ratio, mean difference) used in the synthesis or presentation of results. | Yes- Methods: Data extraction |
| Synthesis methods | 13a | Describe the processes used to decide which studies were eligible for each synthesis (e.g. tabulating the study intervention characteristics and comparing against the planned groups for each synthesis (item #5)). | Yes- Methods: Eligibility criteria & Data extraction |
|  | 13b | Describe any methods required to prepare the data for presentation or synthesis, such as handling of missing summary statistics, or data conversions. | Yes- Methods: Data extraction |
|  | 13c | Describe any methods used to tabulate or visually display results of individual studies and syntheses. | Yes- Methods: Data extraction |
|  | 13d | Describe any methods used to synthesize results and provide a rationale for the choice(s). If meta-analysis was performed, describe the model(s), method(s) to identify the presence and extent of statistical heterogeneity, and software package(s) used. | Yes- Methods: Data analysis |
|  | 13e | Describe any methods used to explore possible causes of heterogeneity among study results (e.g. subgroup analysis, meta-regression). | n/a due too high heterogeneity |
|  | 13f | Describe any sensitivity analyses conducted to assess robustness of the synthesized results. | n/a |
| Reporting bias assessment | 14 | Describe any methods used to assess risk of bias due to missing results in a synthesis (arising from reporting biases). | Yes- Methods: Quality assessment of studies;  Results: Risk of bias |
| Certainty assessment | 15 | Describe any methods used to assess certainty (or confidence) in the body of evidence for an outcome. | Yes- confidence intervals given in Results: Meta-analysis findings and quality appraisal in Results: Risk of bias. |
| **RESULTS** | | |  |
| Study selection | 16a | Describe the results of the search and selection process, from the number of records identified in the search to the number of studies included in the review, ideally using a flow diagram. | Yes- Results: Study selection (Figure 1) |
|  | 16b | Cite studies that might appear to meet the inclusion criteria, but which were excluded, and explain why they were excluded. | Yes- Results: Study selection (Figure 1) |
| Study characteristics | 17 | Cite each included study and present its characteristics. | Yes- Table 1 |
| Risk of bias in studies | 18 | Present assessments of risk of bias for each included study. | Yes- Figure 2 and in Supplementary Figure S1. |
| Results of individual studies | 19 | For all outcomes, present, for each study: (a) summary statistics for each group (where appropriate) and (b) an effect estimate and its precision (e.g. confidence/credible interval), ideally using structured tables or plots. | Yes- Table 2 |
| Results of syntheses | 20a | For each synthesis, briefly summarise the characteristics and risk of bias among contributing studies. | Yes- Results: Study and sample characteristics& Risk of bias |
|  | 20b | Present results of all statistical syntheses conducted. If meta-analysis was done, present for each the summary estimate and its precision (e.g. confidence/credible interval) and measures of statistical heterogeneity. If comparing groups, describe the direction of the effect. | Yes- Results: Meta-analysis findings & Table 2. |
|  | 20c | Present results of all investigations of possible causes of heterogeneity among study results. | n/a |
|  | 20d | Present results of all sensitivity analyses conducted to assess the robustness of the synthesized results. | n/a |
| Reporting biases | 21 | Present assessments of risk of bias due to missing results (arising from reporting biases) for each synthesis assessed. | Yes- Results: Risk of bias |
| Certainty of evidence | 22 | Present assessments of certainty (or confidence) in the body of evidence for each outcome assessed. | Yes- Results: Meta-analysis findings |
| **DISCUSSION** | | |  |
| Discussion | 23a | Provide a general interpretation of the results in the context of other evidence. | Yes- Discussion |
|  | 23b | Discuss any limitations of the evidence included in the review. | Yes- Discussion: Strengths and limitations |
|  | 23c | Discuss any limitations of the review processes used. | Yes- Discussion: Strengths and limitations |
|  | 23d | Discuss implications of the results for practice, policy, and future research. | Yes- Discussion: Implications for practice and research |
| **OTHER INFORMATION** | | |  |
| Registration and protocol | 24a | Provide registration information for the review, including register name and registration number, or state that the review was not registered. | Yes- Methods |
|  | 24b | Indicate where the review protocol can be accessed, or state that a protocol was not prepared. | Yes- Methods |
|  | 24c | Describe and explain any amendments to information provided at registration or in the protocol. | Yes- Methods |
| Support | 25 | Describe sources of financial or non-financial support for the review, and the role of the funders or sponsors in the review. | N/A |
| Competing interests | 26 | Declare any competing interests of review authors. | The authors have no competing interests to report. |
| Availability of data, code and other materials | 27 | Report which of the following are publicly available and where they can be found: template data collection forms; data extracted from included studies; data used for all analyses; analytic code; any other materials used in the review. | Prospero entry publicly available, and all data extracted is presented in Table 1, Table 2, Figure 2 and Figure S1. |

*From:*  Page MJ, McKenzie JE, Bossuyt PM, Boutron I, Hoffmann TC, Mulrow CD, et al. The PRISMA 2020 statement: an updated guideline for reporting systematic reviews. BMJ 2021;372:n71. doi: 10.1136/bmj.n71

For more information, visit: <http://www.prisma-statement.org/>

Supplementary Table S2. Outcome measures of the data extracted in the meta-analyses

| **Domain** | **Outcome measure** | **Outcome Measure Acronyms** | **Study** |
| --- | --- | --- | --- |
| Caregiver Psychological Distress | The Brief Symptom Inventory (Derogatis, 2001) | BSI-18 | Kichler et al., 2013 |
|  | The Center for Epidemiologic Studies Depression Scale (Radloff, 1977) | CESD | Ambrosino et al., 2008; Hillard et al., 2022; Mackey et al., 2016; Mackey et al., 2022; Majidi et al, 2021; Monaghan et al., 2011 |
|  | Depression Anxiety Stress Scales (Depression subscale, 21 items) (Lovibond, 1995) | DASS-21-Depression | Sairanen et al., 2019 |
|  | Depression Anxiety Stress Scales (42 items) (Lovibond, 1993) | DASS-42 | Saghaei et al., 2017; Sabmann et al., 2012; |
|  | Depression Anxiety Stress Scales (42 items, Depression subscale) (Lovibond, 1993) | DASS-42- Depression | Westrupp et al., 2015 |
|  | Patient Health Questionnaire-9 (Kroenke et al., 2001) | PHQ-9 | Jaser et al., 2018 |
|  | Perceived Stress Scale (Cohen et al., 1983) | PSS | Tsiouli et al., 2014 |
|  | Symptom Checklist-90 (Revised, Global Severity Index sub score) (Derogatis, 1993) | SCL-90-R-GSI | Hoff et al., 2005 |
|  | Warwick Edinburg Mental Wellbeing Scale (Tennant et al., 2007)  The World Health Organization-Five Well-Being Index (World Health Organization, 1998). | WEMWBS  WHO-5 | Jones et al., 2024  Commissoriat et al., 2023 |
|  |  |  |  |
| Caregiver Diabetes Psychological Distress | Banion Diabetes Management Concern Questionnaire (Banionet al., 1983) | BDMCQ | Sullivan-Bolyai 2004 |
|  | Diabetes Distress Scale (Polonsky et al., 2005) | DDS-P | Jaser et al., 2018 |
|  | Parents Fear Hypoglycaemia Fear Scale (Adaption of worry subscale) (Clarke et al., 1998) | HFS | Sullivan-Bolyai et al., 2016 |
|  |  |  |  |
|  | Issues in Coping with insulin-dependent diabetes mellitus (Kovacs et al., 1986) | Issues Coping IDDM | Grey et al., 2011 |
|  |  |  |  |
|  |  |  |  |
|  | Problem Areas in Diabetes Scale (Modified Version) (Original: Polonsky et al., 1995) | PAID-Modified | Gregory et al., 2011 |
|  |  |  |  |
|  | Problem Areas in Diabetes Scale (Parent Revised Version) (Markowitz et al., 2012) | PAID-PR | Commissoriat et al., 2023; Hilliard et al., 2020, Patton et al., 2020; Laffel et al., 2021; Jones et al., 2024 |
|  | Paediatric Inventory for Parents (Streisand, et al., 2001) | PIP | Doherty et al., 2014; Mackey et al., 2016; Monaghan et al., 2011 |
|  | Worry Scale (Ireys et al., 1997) | Worry Scale | Sullivan-Bolyai et al., 2010; Sullivan-Bolyai et al., 2011 |
| Caregiver  Diabetes Family Conflict | Diabetes Family Conflict Scale (Rubin et al., 1989) | DFCS | Anderson et al., 1999; Harris et al., 2001; Jaser et al., 2014; Laffel et al., 2003; Majidi et al., 2021; Mayer-Davis et al., 2018 |
|  | Diabetes Family Conflict Scale (Conflict subscale) (Rubin et al., 1989) | DFCS-Conflict | Grey et al., 2011 |
|  | Diabetes Family Conflict Scale Revised (Hood et al., 2007) | DFCS-R | Doherty et al., 2014; Hillard et al., 2020, Holmes et al., 2014; Jaser et al., 2018; Katz et al. 2014; Nansel et al., 2009; Westrupp et al., 2015; Whittemore et al., 2020 |
| Child Psychological Distress | Child Depression Inventory (Kovacs, 1985) | CDI | Jaser et al., 2014 |
|  | Child Depression Inventory 2 (Bae, 2012) | CDI-2 | Majidi et al., 2021 |
|  |  |  |  |
|  | The Center for Epidemiologic Studies Depression Scale (Radloff, 1977) | CESD | Mayer-Davis et al., 2018 |
|  |  |  |  |
| Child Diabetes Psychological Distress | Diabetes Stress Questionnaire (Boardway et al., 1993) | DSQ | Ellis et al., 2005 |
|  | Diabetes Quality of Life Scale for Youth (Short Form Worry Subscale) (Skinner et al., 2006) | DQOLY-SF-Worry | Murphy et al., 2012 |
|  |  |  |  |
|  | Issues in Coping with insulin-dependent diabetes mellitus (Upset subscale) (Kovacs et al., 1986) | IDDM-Upset | Ambrosino et al., 2008 |
|  | Problem Areas in Diabetes Scale (Teen Version) (Weissberg-Benchell et al., 2011) | PAID-T | Hillard et al., 2020 |
|  | Paediatric Quality of Life Inventory (Diabetes module revised, Worry sub score) | PedsQL-Diabetes-R-Worry | Fiallo-Scharer et al., 2019 |
|  | Paediatric Quality of Life Inventory (Diabetes module, Worry sub score) (Varni et al.,2003) | PedsQL-Diabetes-Worry | Christie et al., 2014; Gregory et al., 2011; Holmes et al., 2014 |
| Child Diabetes Family Conflict | Diabetes Family Conflict Scale (Rubin et al., 1989) | DFCS | Harris et al., 2001; Laffel et al., 2003; Majidi et al., 2021; Mayer-Davis et al., 2018 |
|  | Diabetes Family Conflict Scale Revised (Hood et al., 2007), | DFCS-R | Hilliard et al., 2020; Holmes et al., 2014; Jaser et al., 2018; Katz et al., 2014; Nansel et al., 2009 |
| Child Quality of Life | Paediatric Quality of Life Inventory 4.0 (General) (Varni et al., 2001; Varni et al., 2003) | PedsQL-Generic | Holtz et al., 2024; Katz et al., 2014; Kichler et al., 2013; Laffel et al., 2003; Mayer-Davis et al., 2018; Mitchell et al., 2022 ; Nansel et al., 2009 |
| Child Diabetes Quality of Life | Diabetes Quality of Life Scale for Youth (Ingersoll et al., 1991) | DQOL-Y | Ellis et al., 2019 |
|  | Diabetes Quality of Life Scale for Youth (Satisfaction Subscale) (Ingersoll et al., 1991) | DQOL-Y-Satisfaction | Ambrosino et al., 2008 |
|  | Diabetes Quality of Life Scale for Youth (Short Form Worry Subscale) (Skinner et al., 2006) | DQOLY-SF-Worry | Murphy et al., 2012 |
|  |  |  |  |
|  |  |  |  |
|  | Monitoring Individual Needs in Diabetes Youth Questionnaire (de Wit et al., 2012) | MY-Q | Hilliard et al., 2020 |
|  | Paediatric Quality of Life Inventory 3.0 (Diabetes module) (Varni et al., 2003) | Peds QL-Diabetes | Nansel et al., 2009; Kichler et al., 2013; Jaser et al., 2014; Jaser et al., 2018 |
|  |  |  |  |
| Child Blood Glucose | Haemoglobin A1C (HbA1c) | HbA1c (%; Clinic reported) | Anderson et al., 1999; Commissoriat et al., 2023; Coyne et al., 2024; Ellis et al., 2007; Ellis et al., 2017; Ellis et al., 2019; Fiallo-Scharer et al., 2019; Gregory et al., 2011; Grey et al., 2011; Hannon et al,. 2019; Hilliard et al., 2022; Holmes et al., 2014; Holtz et al., 2024; Jaser et al., 2018; Katz et al., 2014; Kichler et al., 2013; Laffel et al., 2003; Laffel et al., 2021; Mackey et al., 2016; Mackey et al., 2022; Mitchell et al., 2022; Murphy et al., 2012; Nansel et al., 2009; Nansel et al., 2012; Patton et al., 2020; Saghaei et al., 2017 (% - assumed); Westrupp et al., 2015; Wysocki et al., 2000; Wysocki et al., 2007 |
|  |  | HbA1c (%; Not stated how it was reported) | Christie et al., 2014; Forsander et al., 2011; Hilliard et al., 2020; Jaser et al., 2014; Saßmann et al., 2012 |
|  |  | HbA1c (Mmol/mol; Clinic reported) | Mayer-Davis et al., 2018 |
|  |  | HbA1c (Mmol/mol; Parent-reported) | Jones et al., 2024 |

**Outcome measure references from Table S1 in alphabetical order:**

Bae Y. Test Review: Children’s Depression Inventory 2 (CDI 2). Journal of Psychoeducational Assessment 2012;30(3):304-8.

Banion CR, Miles MS, Carter MC. Problems of Mothers in Management of Children with Diabetes. Diabetes Care 1983;6(6):548-51.

Boardway RH, Delamater AM, Tomakowsky J, Gutai JP. Stress management training for adolescents with diabetes. Journal of Pediatric Psychology 1993;18(1):29-45.

Clarke WL, Gonder-Frederick LA, Snyder AL, Cox, Daniel J. Maternal fear of hypoglycemia in their children with insulin dependent diabetes mellitus. Journal of Pediatric Endocrinology and Metabolism 1998;11(Supplement):189-94.

Cohen, S., Kamarch, T., & Mermelstein, R. A global measure of perceived stress. Journal of Health and Social Behavior 1983;24, 385.

Derogatis, L. R.. SCL-90-R: Administration, scoring, and procedure manual. Minneapolis, MN: National Computer Systems 1993

Derogatis LR. BSI 18, Brief Symptom Inventory 18: Administration, scoring and procedures manual. NCS Pearson, Incorporated 2001.

Hood KK, Butler DA, Anderson BJ, Laffel LM. Updated and revised Diabetes Family Conflict Scale. Diabetes Care 2007;30(7):1764-9.

Ingersoll GM, Marrero DG. A modified quality-of-life measure for youths: psychometric properties. Diabetes Educ 1991;17(2):114-8.

Ireys, H., Sills, E. M., Kolodner, K. B., & Walsh, B. B.. Family project parent questionnaire manual 1997.

Kovacs M. The Children's Depression, Inventory (CDI). Psychopharmacol Bull. 1985;21(4):995-8.

Lovibond, S. H., & Lovibond, P. F. Manual for the Depression, Anxiety and Stress Scales. Sydney: Psychology Foundation of Australia (1993).

Lovibond, S. H., & Lovibond, P. F. Manual for the Depression, Anxiety and Stress Scales (2nd ed.). Sydney: Psychology Foundation of Australia (1995).

Moos, R. H., & Moos, B. S. Family Environment Scale manual: Development, applications, research. Palo Alto, CA: Mind Garden, Inc (2002).

Polonsky WH, Fisher L, Earles J, Dudl RJ, Lees J, Mullan J, Jackson RA. Assessing psychosocial distress in diabetes: development of the diabetes distress scale. Diabetes Care 2005;28(3):626-31.

Radloff LS. The CES-D Scale. Applied Psychological Measurement 1977;1(3):385-401.

Recklitis CJ, Blackmon JE, Chang G. Validity of the Brief Symptom Inventory-18 (BSI-18) for identifying depression and anxiety in young adult cancer survivors: Comparison with a Structured Clinical Diagnostic Interview. Psychological Assessment 2017;29(10):1189-200.

Rubin R, Young-Hyman D, Peyrot M. Parent-child responsibility and conflict in diabetes care. Diabetes 1989;38(Suppl 2):28A.

Skinner TC, Hoey H, McGee HM, Skovlund SE. A short form of the Diabetes Quality of Life for Youth questionnaire: exploratory and confirmatory analysis in a sample of 2,077 young people with type 1 diabetes mellitus. Diabetologia 2006;49(4):621-8.

Staehr Johansen K: The use of well-being measures in primary health care - the DepCare project; in World Health Organization, Regional Office for Europe: Well-Being Measures in Primary Health Care - the DepCare Project. Geneva, World Health Organization, 1998, target 12, E60246.

de Wit M, Winterdijk P, Aanstoot HJ, Anderson B, Danne T, Deeb L, et al. Assessing diabetes-related quality of life of youth with type 1 diabetes in routine clinical care: the MIND Youth Questionnaire (MY-Q). Pediatr Diabetes 2012;13(8):638-46.

Kovacs M, Brent D, Steinberg T, Paulauskas S, Reid J. Children's self-reports of psychologic adjustment and coping strategies during first year of insulin-dependent diabetes mellitus. Diabetes care 1986;9(5):472-9.

Kroenke K, Spitzer RL, Williams JB. The PHQ-9: validity of a brief depression severity measure. J Gen Intern Med 2001;16(9):606-13.

Markowitz JT, Volkening LK, Butler DA, Antisdel-Lomaglio J, Anderson BJ, Laffel LM. Re-examining a measure of diabetes-related burden in parents of young people with Type 1 diabetes: the Problem Areas in Diabetes Survey - Parent Revised version (PAID-PR). Diabet Med 2012;29(4):526-30.

Polonsky WH, Anderson BJ, Lohrer PA, Welch G, Jacobson AM, Aponte JE, Schwartz CE. Assessment of diabetes-related distress. Diabetes Care 1995;18(6):754-60.

Prinz RJ, Foster S, Kent RN, O'Leary KD. Multivariate assessment of conflict in distressed and nondistressed mother‐adolescent dyads. Journal of applied behavior analysis 1979;12(4):691-700.

Robin AL, Koepke T, Moye A. Multidimensional assessment of parent-adolescent relations. Psychological Assessment: A Journal of Consulting and Clinical Psychology 1990;2(4):451-9.

Streisand R, Braniecki S, Tercyak KP, Kazak AE. Childhood illness-related parenting stress: the pediatric inventory for parents. J Pediatr Psychol 2001;26(3):155-62.

Tennant R, Hiller L, Fishwick R, Platt S, Joseph S, Weich S, et al. The Warwick-Edinburgh Mental Well-being Scale (WEMWBS): development and UK validation. Health and Quality of Life Outcomes 2007;5(1):63.

Varni JW, Burwinkle TM, Jacobs JR, Gottschalk M, Kaufman F, Jones KL. The PedsQL in type 1 and type 2 diabetes: reliability and validity of the Pediatric Quality of Life Inventory Generic Core Scales and type 1 Diabetes Module. Diabetes Care 2003;26(3):631-7.

Varni JW, Delamater AM, Hood KK, Raymond JK, Chang NT, Driscoll KA, et al. PedsQL 3.2 Diabetes Module for Children, Adolescents, and Young Adults: Reliability and Validity in Type 1 Diabetes. Diabetes Care 2018;41(10):2064-71.

Varni JW, Seid M, Kurtin PS. PedsQL 4.0: reliability and validity of the Pediatric Quality of Life Inventory version 4.0 generic core scales in healthy and patient populations. Med Care 2001;39(8):800-12.

Weissberg-Benchell J, Antisdel-Lomaglio J. Diabetes-specific emotional distress among adolescents: feasibility, reliability, and validity of the problem areas in diabetes-teen version. Pediatric diabetes 2011;12.

World Health Organization. (1998). Wellbeing measures in primary health care/the DepCare Project: report on a WHO meeting: Stockholm, Sweden, 12–13 February 1998 (No. WHO/EURO: 1998-4234-43993-62027). World Health Organization. Regional Office for Europe.

Supplementary Table S3. References for the papers included in the systematic review in alphabetical order

| **Alphabetical order** | **Reference** |
| --- | --- |
| 1 | Ambrosino JM, Fennie K, Whittemore R, Jaser S, Dowd MF, Grey M. Short‐term effects of coping skills training in school‐age children with type 1 diabetes. Pediatric diabetes 2008;9(Part 2):74-82. |
| 2 | Anderson BJ, Brackett J, Ho J, Laffel LMB. An office-based intervention to maintain parent-adolescent teamwork in diabetes management - Impact on parent involvement, family conflict, and subsequent glycemic control. Diabetes Care 1999;22(5):713-21. |
| 3 | Christie D, Thompson R, Sawtell M, Allen E, Cairns J, Smith F, et al. Structured, intensive education maximising engagement, motivation and long-term change for children and young people with diabetes: A cluster randomised controlled trial with integral process and economic evaluation - the CASCADE study. Health Technology Assessment 2014;18(8):1-202. |
| 4 | Commissariat PV, DiMeglio LA, Kanapka LG, Laffel LM, Miller KM, Anderson BJ ... & Deuser A. Twelve‐month psychosocial outcomes of continuous glucose monitoring with behavioural support in parents of young children with type 1 diabetes. Diabetic Medicine 2023, 40(8), e15120. |
| 5 | Coyne I, Sleath B, Surdey J, Pembroke S, Hilliard C, Chechalk K & Roche E. Intervention to promote adolescents’ communication and engagement in diabetes clinic encounters: A pilot randomized controlled trial. Patient Education and Counselling 2024; 126, 108322. |
| 6 | Doherty FM, Calam R, Sanders MR. 'Positive Parenting Program (Triple P) for families of adolescents with Type 1 diabetes: A randomized controlled trial of self-directed teen Triple P': Erratum. Journal of Pediatric Psychology 2014;39(10):1175. Erratum for: Journal of Pediatric Psychology 38(8): 846-858. |
| 7 | Ellis DA, Frey MA, Naar-King S, Templin T, Cunningham PB, Cakan N. The effects of multisystemic therapy on diabetes stress among adolescents with chronically poorly controlled type 1 diabetes: findings from a randomized, controlled trial. Pediatrics 2005;116(6):e826-e832. |
| 8 | Ellis DA, Templin T, Naar-King S, Frey MA, Cunningham PB, Podolski CL, Cakan N. Multisystemic therapy for adolescents with poorly controlled type 1 diabetes: Stability of treatment effects in a randomized controlled trial. Journal of Consulting and Clinical Psychology 2007;75(1):168‐174. |
| 9 | Ellis DA, Carcone AI, Ondersma SJ, Naar-King S, Dekelbab B, Moltz K. Brief computer-delivered intervention to increase parental monitoring in families of African American adolescents with type 1 Diabetes: A randomized controlled trial. Telemedicine and E-Health 2017;23(6):493-502. |
| 10 | Ellis DA, Carcone AI, Naar-King S, Rajkumar D, Palmisano G, Moltz K. Adaptation of an evidence-based diabetes management intervention for delivery in community settings: Findings from a pilot randomized effectiveness trial. Journal of Pediatric Psychology 2019;44(1):110-125. |
| 11 | Fiallo-Scharer R, Palta M, Chewning BA, Rajamanickam V, Wysocki T, Wetterneck TB, Cox ED. Impact of family-centered tailoring of pediatric diabetes self-management resources. Pediatric Diabetes 2019;20(7):1016-1024. |
| 12 | Forsander G, Sundelin J, Persson B. Influence of the initial management regimen and family social situation on glycemic control and medical care in children with type 1 diabetes mellitus. Acta Paediatrica 2000;89(12):1462-1468. |
| 13 | Gregory JW, Robling M, Bennert K, Channon S, Cohen D, Crowne E, et al. Development and evaluation by a cluster randomised trial of a psychosocial intervention in children and teenagers experiencing diabetes: the DEPICTED study. Health Technology Assessment 2011;15(29):3‐202. |
| 14 | Grey M, Jaser SS, Whittemore R, Jeon S, Lindemann E. Coping skills training for parents of children with type 1 diabetes: 12-month outcomes. Nursing Research 2011;60(3):173-181. |
| 15 | Hannon TS, Yazel‐Smith LG, Hatton AS, Stanton JL, Moser EA, Li X, Carroll AE. Advancing diabetes management in adolescents: comparative effectiveness of mobile self‐monitoring blood glucose technology and family‐centered goal setting. Pediatric Diabetes 2018;19(4):776-781. |
| 16 | Harris MA, Greco P, Wysocki T, White NH. Family therapy with adolescents with diabetes: A litmus test for clinically meaningful change. Families, Systems, & Health 2001;19(2):159-168. |
| 17 | Hilliard ME, Cao VT, Eshtehardi SS, Minard CG, Saber R, Thompson D, et al. Type 1 doing well: Pilot feasibility and acceptability study of a strengths-based mhealth app for parents of adolescents with type 1 diabetes. Diabetes Technology & Therapeutics 2020;22(11):835-845. |
| 18 | Hilliard ME, Tully C, Monaghan M, Hildebrandt T, Wang CH, Barber JR, et al. First steps: Primary outcomes of a randomized, stepped-care behavioral clinical trial for parents of young children with new-onset type 1 diabetes. Diabetes Care 2022;45(10):2238-2246. |
| 19 | Hoff AL, Mullins LL, Gillaspy SR, Page MC, Van Pelt JC, Chaney JM. An intervention to decrease uncertainly and distress among parents of children newly diagnosed with diabetes: a pilot study. Families, systems and health 2005;23(3):329‐342. |
| 20 | Holmes CS, Chen R, Mackey E, Grey M, Streisand R. Randomized clinical trial of clinic-integrated, low-intensity treatment to prevent deterioration of disease care in adolescents with type 1 diabetes. Diabetes Care 2014;37(6):1535-1543. |
| 21 | Holtz BE, Mitchell KM, Holmstrom AJ, Hershey DS, Cotten SR, Dunneback JK, et al. The effect of an mHealth intervention for adolescents with type 1 diabetes and their parents. Journal of Telemedicine and Telecare 2024;30(7): 1155-1162. |
| 22 | *Husted GR, Thorsteinsson B, Esbensen BA, Gluud C, Winkel P, Hommel E, Zoffmann V. Effect of guided self-determination youth intervention integrated into outpatient visits versus treatment as usual on glycemic control and life skills: A randomized clinical trial in adolescents with type 1 diabetes. Trials 2014;15(321):1-12. |
| 23 | Jaser SS, Patel N, Rothman RL, Choi L, Whittemore R. A randomized pilot of a positive psychology intervention to improve adherence in adolescents with type 1 diabetes. Diabetes Educator 2014;40(5):659-667. |
| 24 | Jaser SS, Lord JH, Savin K, Gruhn M, Rumburg T. Developing and testing an intervention to reduce distress in mothers of adolescents with type 1 diabetes. Clinical Practice in Pediatric Psychology 2018;6(1):19-30. |
| 25 | Jones CJ, Read R, O'Donnell N, Wakelin K, John M, Skene SS, et al. PRIORITY Trial: Results from a feasibility randomised controlled trial of a psychoeducational intervention for parents to prevent disordered eating in children and young people with type 1 diabetes. Diabetic Medicine 2024 ; doi; 41(4), e15263. |
| 26 | Katz ML, Volkening LK, Butler DA, Anderson BJ, Laffel LM. Family‐based psychoeducation and care ambassador intervention to improve glycemic control in youth with type 1 diabetes: a randomized trial. Pediatric diabetes 2014;15(2):142-50. |
| 27 | Kichler JC, Marik P, Kaugars AS, Nabors L, Alemzadeh R. Effectiveness of groups for adolescents with type 1 diabetes mellitus and their parents. Families, Systems & Health: The Journal of Collaborative Family HealthCare 2013;31(3):280-293. |
| 28 | Laffel LMB, Vangsness L, Connell A, Goebel-Fabbri A, Butler D, Anderson BJ. Impact of ambulatory, family-focused teamwork intervention on glycemic control in youth with type 1 diabetes. The Journal of Pediatrics 2003;142(4):409-16. |
| 29 | Laffel L, Harrington K, Hanono A, Naik N, Ambler-Osborn L, Schultz A, et al. A randomized clinical trial assessing continuous glucose monitoring (cgm) use with standardized education with or without a family behavioral intervention compared with fingerstick blood glucose monitoring in very young children with type 1 diabetes. Diabetes Care 2021;44(2):464-472. |
| 30 | *Lehmkuhl HD, Storch EA, Cammarata C, Meyer K, Rahman O, Silverstein J, et al. Telehealth behavior therapy for the management of type 1 diabetes in adolescents. Journal of Diabetes Science And Technology 2010;4(1):199-208. |
| 31 | Mackey ER, Herbert L, Monaghan M, Cogen F, Wang J, Streisand R. The feasibility of a pilot intervention for parents of young children newly diagnosed with type 1 diabetes. Clinical Practice in Pediatric Psychology 2016;4(1):35-50. |
| 32 | Mackey ER, Tully C, Rose M, Hamburger S, Wang J, Herrera N, et al. Promoting glycemic control in young children with type 1 diabetes: Results from a pilot intervention for parents. Families, Systems, & Health 2022;40(2):239. |
| 33 | Majidi S, Reid MW, Fogel J, Anderson B, Klingensmith GJ, Cain C, et al. Psychosocial outcomes in young adolescents with type 1 diabetes participating in shared medical appointments. Pediatric Diabetes 2021;22(5):787-95. |
| 34 | Mayer-Davis EJ, Maahs DM, Seid M, Crandell J, Bishop FK, Driscoll KA, et al. Efficacy of the flexible lifestyles empowering change intervention on metabolic and psychosocial outcomes in adolescents with type 1 diabetes (FLEX): A randomised controlled trial. Lancet Child & Adolescent Health 2018;2(9):635-46. |
| 35 | Mitchell AE, Morawska A, Lohan A, Filus A, Batch J. Randomised controlled trial of the Healthy Living Triple P–Positive Parenting Program for families of children with type 1 diabetes. Journal of Child Health Care 2022; 0(0):1-21. |
| 36 | Monaghan M, Hilliard ME, Cogen FR, Streisand R. Supporting parents of very young children with type 1 diabetes: Results from a pilot study. Patient Education and Counseling 2011;82(2):271-274. |
| 37 | *Murphy HR, Wadham C, Rayman G, Skinner TC. Approaches to integrating paediatric diabetes care and structured education: experiences from the Families, Adolescents, and Children's Teamwork Study (FACTS). Diabetic Medicine 2007;24(11):1261-1268. |
| 38 | Murphy HR, Wadham C, Hassler-Hurst J, Rayman G, Skinner TC. Randomized trial of a diabetes self-management education and family teamwork intervention in adolescents with type 1 diabetes. Diabetic Medicine 2012;29(8):e249-e254. |
| 39 | Nansel TR, Anderson BJ, Laffel LMB, Simons-Morton BG, Weissberg-Benchell J, Wysocki T, et al. A multisite trial of a clinic-integrated intervention for promoting family management of pediatric type 1 diabetes: Feasibility and design. Pediatric Diabetes 2009;10(2):105-15. |
| 40 | Nansel TR, Iannotti RJ, Liu AY. Clinic-integrated behavioral intervention for families of youth with type 1 diabetes: Randomized clinical trial. Pediatrics 2012;129(4):e866-e873. |
| 41 | Nansel TR, Laffel LMB, Haynie DL, Mehta SN, Lipsky LM, Volkening LK, et al. Improving dietary quality in youth with type 1 diabetes: Randomized clinical trial of a family-based behavioral intervention. International Journal of Behavioral Nutrition and Physical Activity 2015;12(58):1-12. |
| 42 | Patton SR, Clements MA, Marker AM, Nelson EL. Intervention to reduce hypoglycemia fear in parents of young kids using video-based telehealth (REDCHiP). Pediatric Diabetes 2020;21(1):112-119. |
| 43 | Saghaei M, Omidi P, Dehkordi EH, Safavi P. The effectiveness of cognitive-function stress management training in glycemic control in children and in mental health of mother caring for child with type 1 diabetes mellitus. Diabetes & Metabolic Syndrome 2017;11(S2):S925-S928. |
| 44 | Sairanen E, Lappalainen R, Lappalainen P, Kaipainen K, Carlstedt F, Anclair M, Hiltunen A. Effectiveness of a web-based Acceptance and Commitment Therapy intervention for wellbeing of parents whose children have chronic conditions: A randomized controlled trial. Journal of Contextual Behavioral Science 2019;13:94-102. |
| 45 | Saßmann H, de Hair M, Danne T, Lange K, Sassmann H. Reducing stress and supporting positive relations in families of young children with type 1 diabetes: A randomized controlled study for evaluating the effects of the DELFIN parenting program. BMC Pediatrics 2012;12(1):1-11. |
| 46 | Sullivan-Bolyai S, Grey M, Deatrick J, Gruppuso P, Giraitis P, Tamborlane W. Helping other mothers effectively work at raising young children with Type 1 diabetes. The Diabetes Educator 2004;30(3):476-84. |
| 47 | Sullivan-Bolyai S, Bova C, Leung K, Trudeau A, Lee M, Gruppuso P. Social Support to Empower Parents (STEP): an intervention for parents of young children newly diagnosed with type 1 diabetes. The Diabetes Educator 2010;36(1):88-97. |
| 48 | Sullivan-Bolyai S, Bova C, Lee M, Gruppuso PA. Mentoring fathers of children newly diagnosed with T1DM. MCN The American Journal of Maternal Child Nursing 2011;36(4):224-231. |
| 49 | Sullivan-Bolyai S, Crawford S, Johnson K, Ramchandani N, Quinn D, D’Alesandro B, et al. PREP-T1 (preteen re-education with parents–Type 1 diabetes) feasibility intervention results. Journal of Family Nursing 2016;22(4):579-605. |
| 50 | Tsiouli E, Pavlopoulos V, Alexopoulos EC, Chrousos G, Darviri C. Short-term impact of a stress management and health promotion program on perceived stress, parental stress, health locus of control, and cortisol levels in parents of children and adolescents with diabetes type 1: A pilot randomized controlled trial. Explore 2014;10(2):88-98. |
| 51 | Van Name MA, Kanapka LG, DiMeglio LA, Miller KM, Albanese-O’Neill A, Commissariat P ... & DeSalvo DJ. (2023). Long-term continuous glucose monitor use in very young children with type 1 diabetes: one-year results from the SENCE study. Journal of Diabetes Science and Technology, 17(4), 976-987. |
| 52 | Westrupp EM, Northam E, Lee KJ, Scratch SE, Cameron F. Reducing and preventing internalizing and externalizing behavior problems in children with Type 1 diabetes: A randomized controlled trial of the Triple P-Positive Parenting Program. Pediatric diabetes 2015;16(7):554-563. |
| 53 | *Whittemore R, Coleman J, Delvy R, Zincavage R, Ambrosoli JA, Shi L, et al. An eHealth program for parents of adolescents with t1dm improves parenting stress: A Randomized control trial. The Diabetes Educator 2020;46(1):62-72. |
| 54 | Wysocki T, Harris MA, Greco P, Bubb J, Danda CE, Harvey LM, et al. Randomized, controlled trial of behavior therapy for families of adolescents with insulin-dependent diabetes mellitus. Journal of Pediatric Psychology 2000;25(1):23-33. |
| 55 | *Wysocki T, Greco P, Harris MA, Bubb J, White NH. Behavior therapy for families of adolescents with diabetes - Maintenance of treatment effects. Diabetes Care 2001;24(3):441-6. |
| 56 | *Wysocki T, Harris MA, Buckloh LM, Mertlich D, Lochrie AS, Taylor A, et al. Effects of Behavioral Family Systems Therapy for diabetes on adolescents' family relationships, treatment adherence and metabolic control. Journal of Pediatric Psychology 2006;31(9):928-44. |
| 57 | Wysocki T, Harris MA, Buckloh LM, Mertlich D, Lochrie AS, Mauras N, White NH. Randomized trial of behavioral family systems therapy for diabetes - Maintenance of effects on diabetes outcomes in adolescents. Diabetes Care 2007;30(3):555-560. |
| 58 | *Wysocki T, Harris MA, Buckloh LM, Mertlich D, Lochrie AS, Taylor A, et al. Randomized, controlled trial of Behavioral Family Systems Therapy for Diabetes: maintenance and generalization of effects on parent-adolescent communication. Behavior Therapy 2008;39(1):33-46. |

*=Studies unable to be included in meta-analysis due to insufficient data reporting/ data in incorrect format to be included.

**Supplementary Figures**

Supplementary Figure S1. Search Terms

Search Terms

parents OR caregivers OR mother OR mum OR mom OR father OR dad OR guardian OR famil*

AND

diabetes OR “type 1 diabetes” OR T1D OR “type one diabetes” OR T1DM

AND

counselling OR “cognitive behavio* therapy” OR “family systems therapy” OR “family therapy” OR “systemic therapy” OR “psychodynamic therapy” OR therapy OR “psychological intervention*” OR “parenting intervention*” OR “behavio* therapy” OR “behavio* approach*” OR therap* OR treatment OR experiment* OR psychology* OR support* OR psycho-education* OR OR psychoeducation* OR psych* OR “psychological education” OR psychosocial OR psycho-social OR self-help OR “problem solving” OR problem-solving OR cogniti* OR family OR “interpersonal psychotherapy” OR IPT OR psychotherapy

AND

Intervention* OR Random* OR Trial OR RCT

Supplementary Figure S2. Full summary of bias domains using Risk of Bias 2 Tool


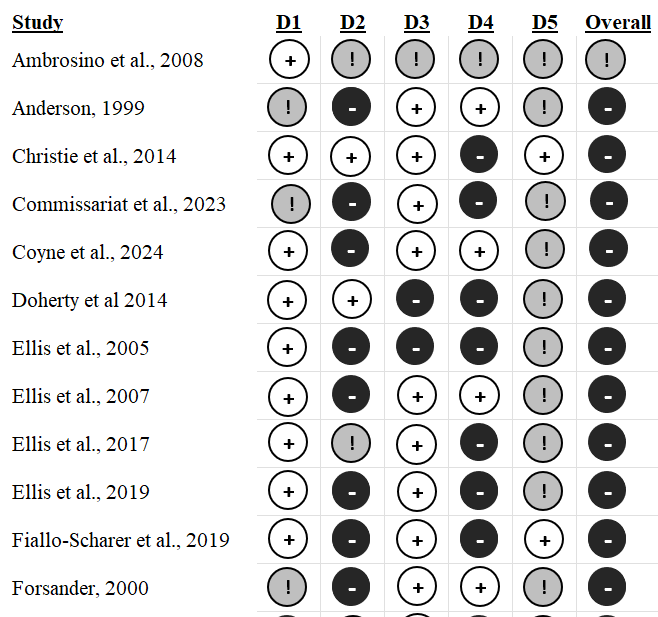

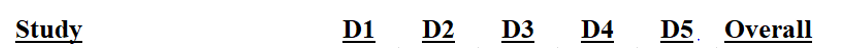

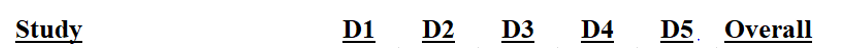


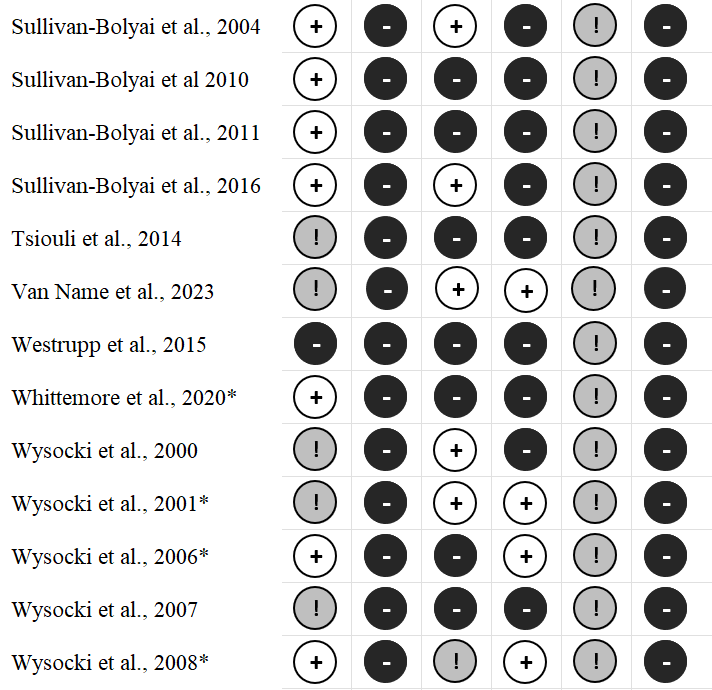

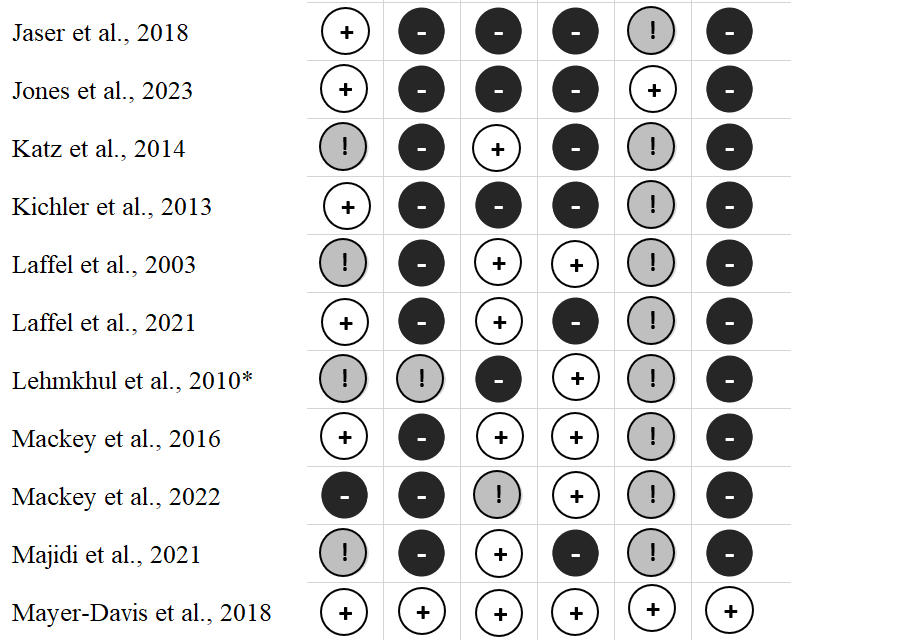

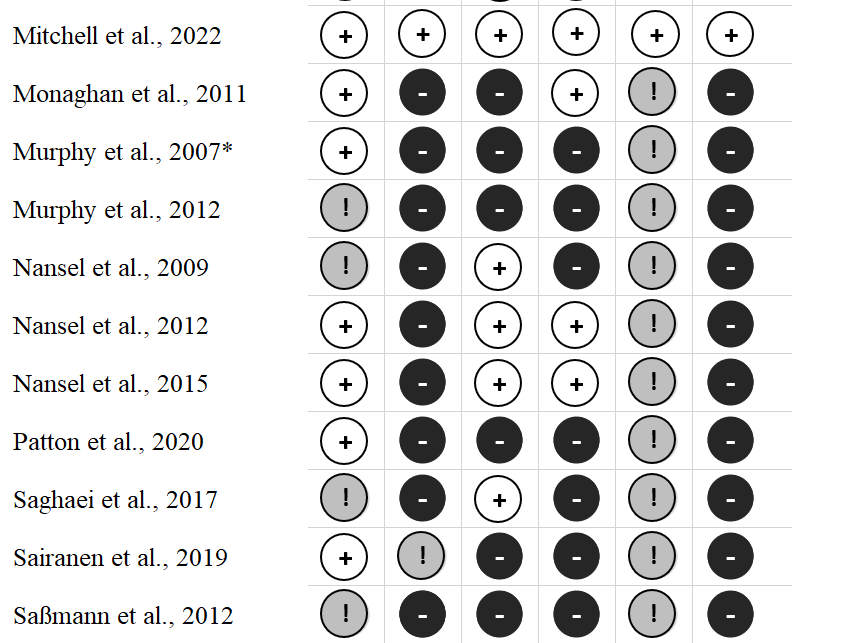

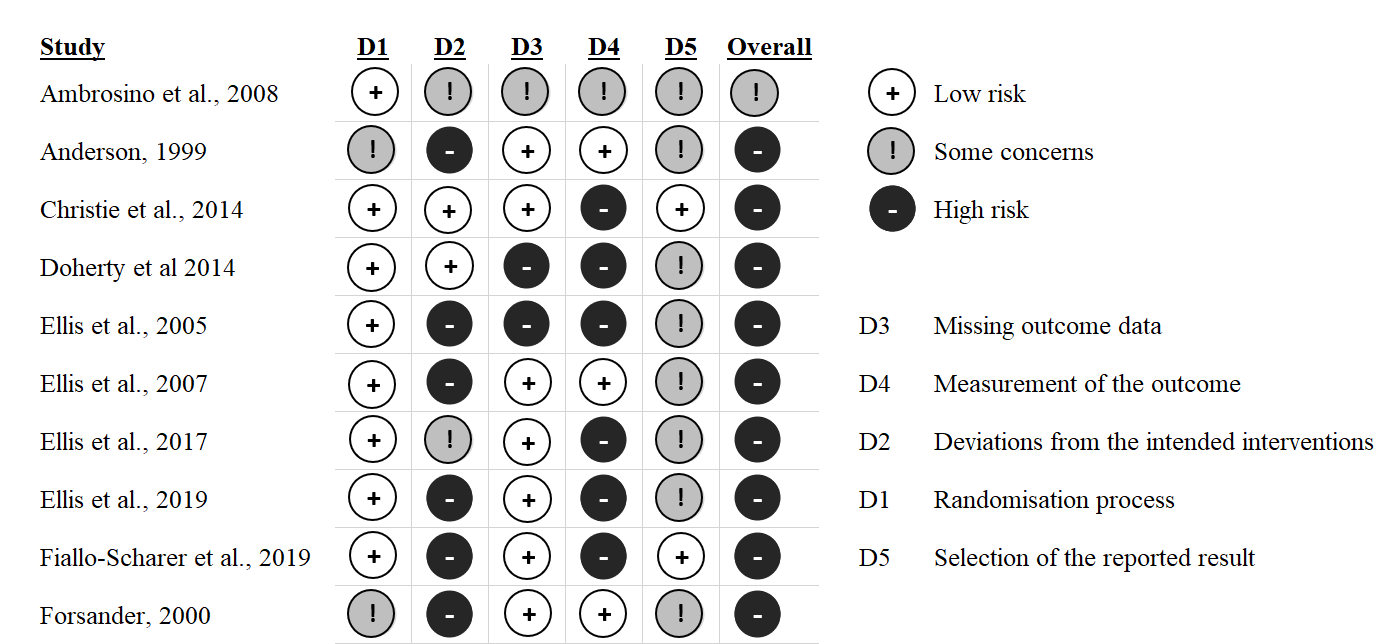


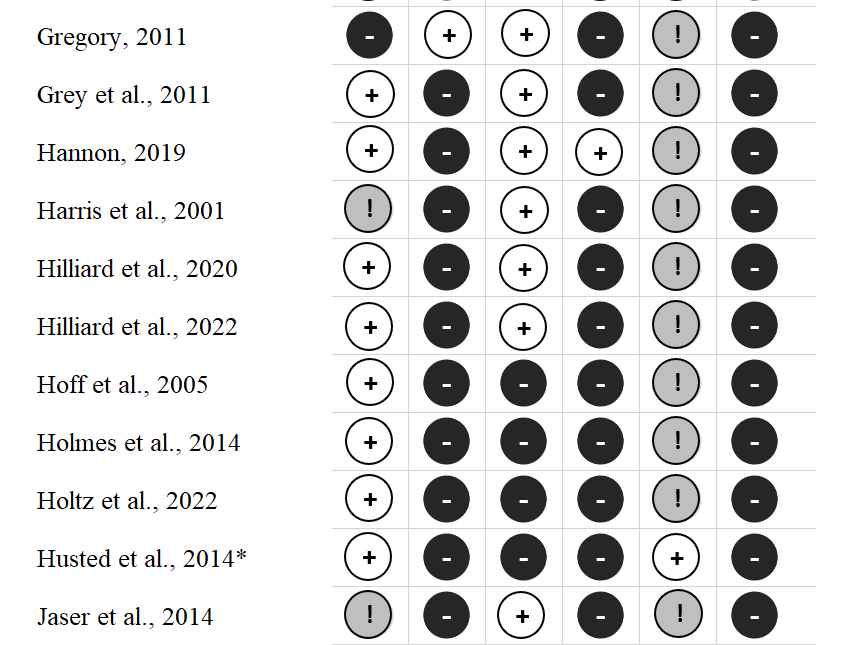

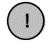


**Key**


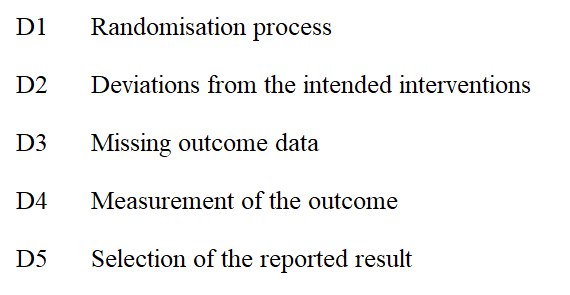


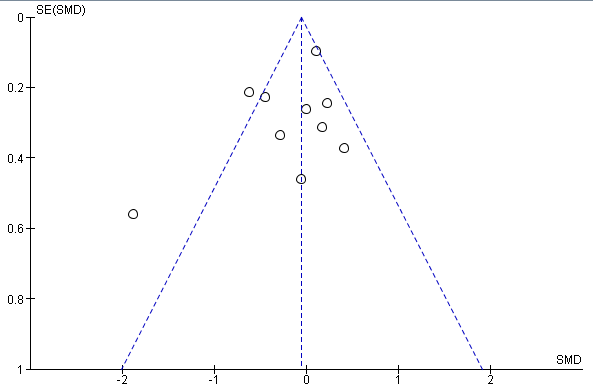

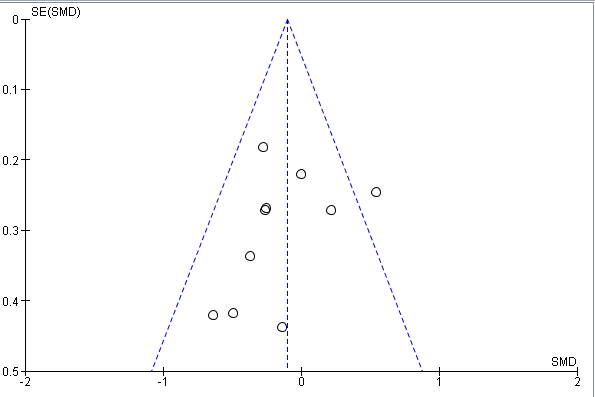

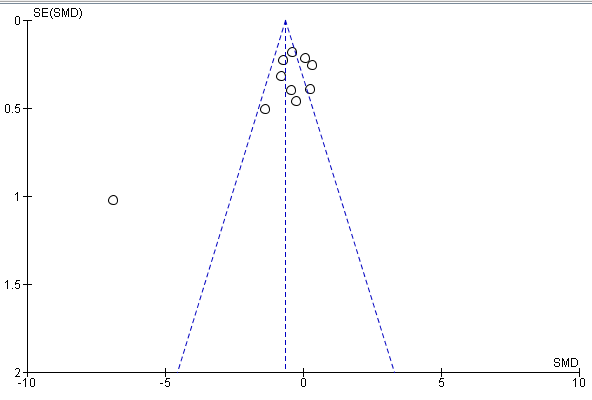
Supplementary Figure S3. Funnel plots for meta-analyses with ten or more studies

Caregiver diabetes psychological distress immediately post-intervention (n=10)

Caregiver psychological distress short term follow-up (n=10)

Caregiver psychological distress immediately post-intervention (n=10)


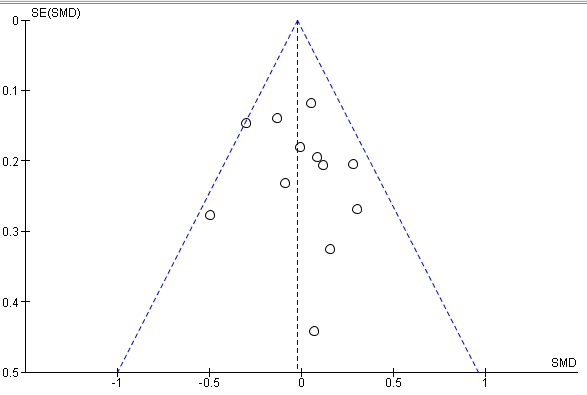

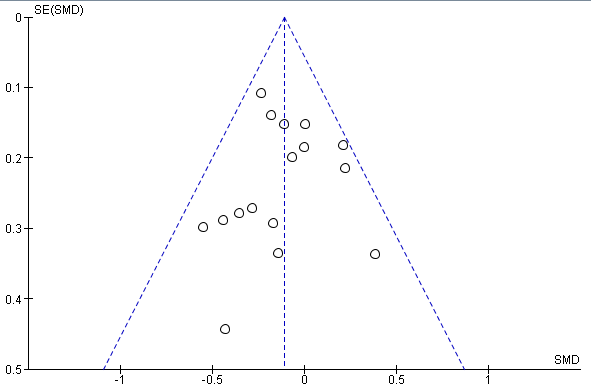

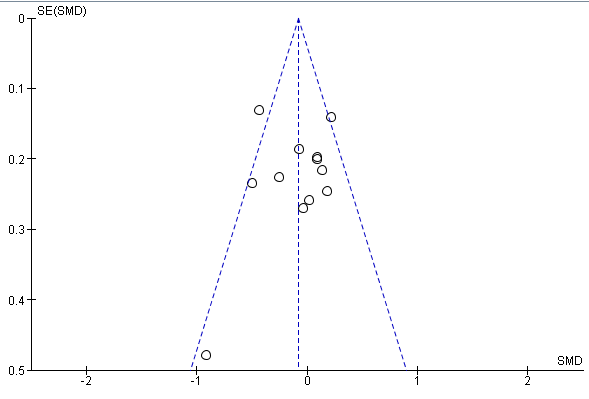


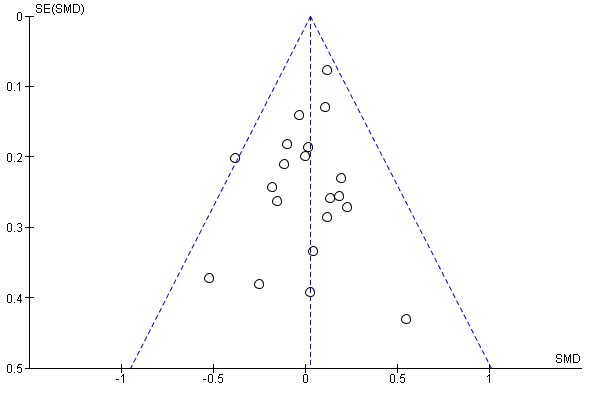
Z

Child blood glucose immediately post-intervention (n=20)

Child blood glucose long term follow-up (n=12)

Child blood glucose short term follow up (n=16)

Caregiver diabetes family conflict immediately post-intervention (n=12)

Immediate
